# ZAKα/P38 kinase signaling pathway regulates hematopoiesis by activating the NLRP1 inflammasome

**DOI:** 10.15252/emmm.202318142

**Published:** 2023-09-07

**Authors:** Lola Rodríguez‐Ruiz, Juan M Lozano‐Gil, Elena Naranjo‐Sánchez, Elena Martínez‐Balsalobre, Alicia Martínez‐López, Christophe Lachaud, Miguel Blanquer, Toan K Phung, Diana García‐Moreno, María L Cayuela, Sylwia D Tyrkalska, Ana B Pérez‐Oliva, Victoriano Mulero

**Affiliations:** ^1^ Departmento de Biología Celular e Histología, Facultad de Biología Universidad de Murcia Murcia Spain; ^2^ Instituto Murciano de Investigación Biosanitaria (IMIB)‐Pascual Parrilla Murcia Spain; ^3^ Centro de Investigación Biomédica en Red de Enfermedades Raras (CIBERER) Instituto de Salud Carlos III Madrid Spain; ^4^ Hospital Clínico Universitario Virgen de la Arrixaca Murcia Spain; ^5^ Aix‐Marseille University, Inserm, CNRS, Institut Paoli‐Calmettes, CRCM Marseille France; ^6^ Departamento de Medicina y Unidad de Terapia Celular y Trasplante Hematopoyético, Facultad de Medicina Universidad de Murcia Murcia Spain; ^7^ MRC PPU, Sir James Black Centre, School of Life Sciences University of Dundee Dundee UK

**Keywords:** anemia, HSPCs, inflammation, neutrophilia, zebrafish, Haematology, Immunology

## Abstract

Chronic inflammatory diseases are associated with hematopoietic lineage bias, including neutrophilia and anemia. We have recently identified that the canonical inflammasome mediates the cleavage of the master erythroid transcription factor GATA1 in hematopoietic stem and progenitor cells (HSPCs). We report here that genetic inhibition of Nlrp1 resulted in reduced number of neutrophils and increased erythrocyte counts in zebrafish larvae. We also found that the NLRP1 inflammasome in human cells was inhibited by LRRFIP1 and FLII, independently of DPP9, and both inhibitors regulated hematopoiesis. Mechanistically, erythroid differentiation resulted in ribosomal stress‐induced activation of the ZAKα/P38 kinase axis which, in turn, phosphorylated and promoted the assembly of NLRP1 in both zebrafish and human. Finally, inhibition of Zaka with the FDA/EMA‐approved drug Nilotinib alleviated neutrophilia in a zebrafish model of neutrophilic inflammation and promoted erythroid differentiation and GATA1 accumulation in K562 cells. In conclusion, our results reveal that the NLRP1 inflammasome regulates hematopoiesis and pave the way to develop novel therapeutic strategies for the treatment of hematopoietic alterations associated with chronic inflammatory and rare diseases.

## Graphical abstract

Deregulation of inflammasome activity contributes to hematopoietic alterations associated to chronic inflammatory diseases, including neutrophilia and anemia. Zebrafish models and human blood cells were used to identify the critical role of the NLRP1 inflammasome on the regulation of hematopoiesis and possible therapeutic targets for clinical intervention.

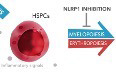

## Introduction

Inflammasomes are cytosolic pattern recognition receptors (PRRs) that detect pathogen‐ and danger‐associated molecular patterns (PAMPs and DAMPs) (Guo *et al*, 2015). The relevance of inflammasomes in human biology is highlighted by their involvement in numerous inflammatory disorders (Broderick *et al*, 2015; Guo *et al*, 2015). Inflammasome assembly is a complex process and involves the activation of a sensor, which belongs to the NOD‐like receptor (NLR) or absent of melanoma 2‐like receptor (AIM2) family, recruitment and polymerization of the adaptor Apoptosis‐associated speck‐like protein containing a CARD (ASC), and activation of the effector caspase‐1 (CASP1) or CASP4 (CASP11 in mice; Broderick *et al*, 2015). The NLR family pyrin domain containing 1 (NLRP1) inflammasome was the first inflammasome identified more than 20 years ago (Martinon *et al*, 2002). However, its activation mechanism has remained obscure, probably due to the presence of different NLPR1 paralogs in mice, and their obvious differences with human NLRP1 in structure, ASC requirement and activation mechanisms (Bauernfried & Hornung, 2022). NLPR1 is highly expressed in the skin and, in fact, gain‐of‐function mutations result in inflammatory skin diseases and cancer susceptibility syndromes primarily driven by hyperproduction of interleukin‐1β (IL‐1β) (Zhong *et al*, 2016). Similarly, loss‐of‐function mutations of its direct inhibitor Dipeptidyl Peptidase 9 (DPP9) lead to a lethal autoinflammatory disorder characterized by hyperproduction of IL‐1β (Harapas *et al*, 2022). Recent studies revealed two physiological activators of human NLRP1: (i) viral 3C proteases that cleave its N terminus and their mode of action was in line with the functional degradation reported for mice NLRP1B (Robinson *et al*, 2020; Tsu *et al*, 2021), and (ii) double‐stranded RNA (dsRNA) (Bauernfried *et al*, 2021). Notably, dsRNA did not activate mouse NLRP1B (Bauernfried *et al*, 2021). More recently, two independent laboratories demonstrated that human, but not mouse, NLRP1 is activated by direct phosphorylation of its linker region by P38 mitogen‐activated protein kinase (MAPK), which is activated by ZAKα (Mitogen‐Activated Protein Kinase Kinase Kinase 20, MAP3K20) in response to either ribotoxic stress response to UV or alphavirus (Harapas *et al*, 2022; Jenster *et al*, 2023). This activation model also involved ubiquitination and N terminal degradation of NLRP1 but is independent of DPP9.

We have recently shown that the canonical inflammasome is activated in hematopoietic stem and progenitor cells (HSPCs) and regulates erythroid‐myeloid lineage decision by cleaving the erythroid transcription factor GATA binding protein 1 (GATA1; Tyrkalska *et al*, 2019). Although this mechanism is conserved in zebrafish and human, the specific NLR sensor involved and its activation mechanism is unknown. Here we report that the NLRP1 inflammasome regulates erythroid‐myeloid lineage decision in HSPCs upon its phosphorylation and activation by the ZAKα/P38 axis. We also found that its activity is negatively regulated by LRR Binding FLII Interacting Protein 1 (LRRFIP1) and Flightless‐1 homolog (FLII), independently of DPP9. Furthermore, we show the potential clinical relevance of these findings to treating neutrophilic inflammation and congenital anemia.

## Results

### The NLRP1 inflammasome regulates hematopoiesis

**Figure 1:**
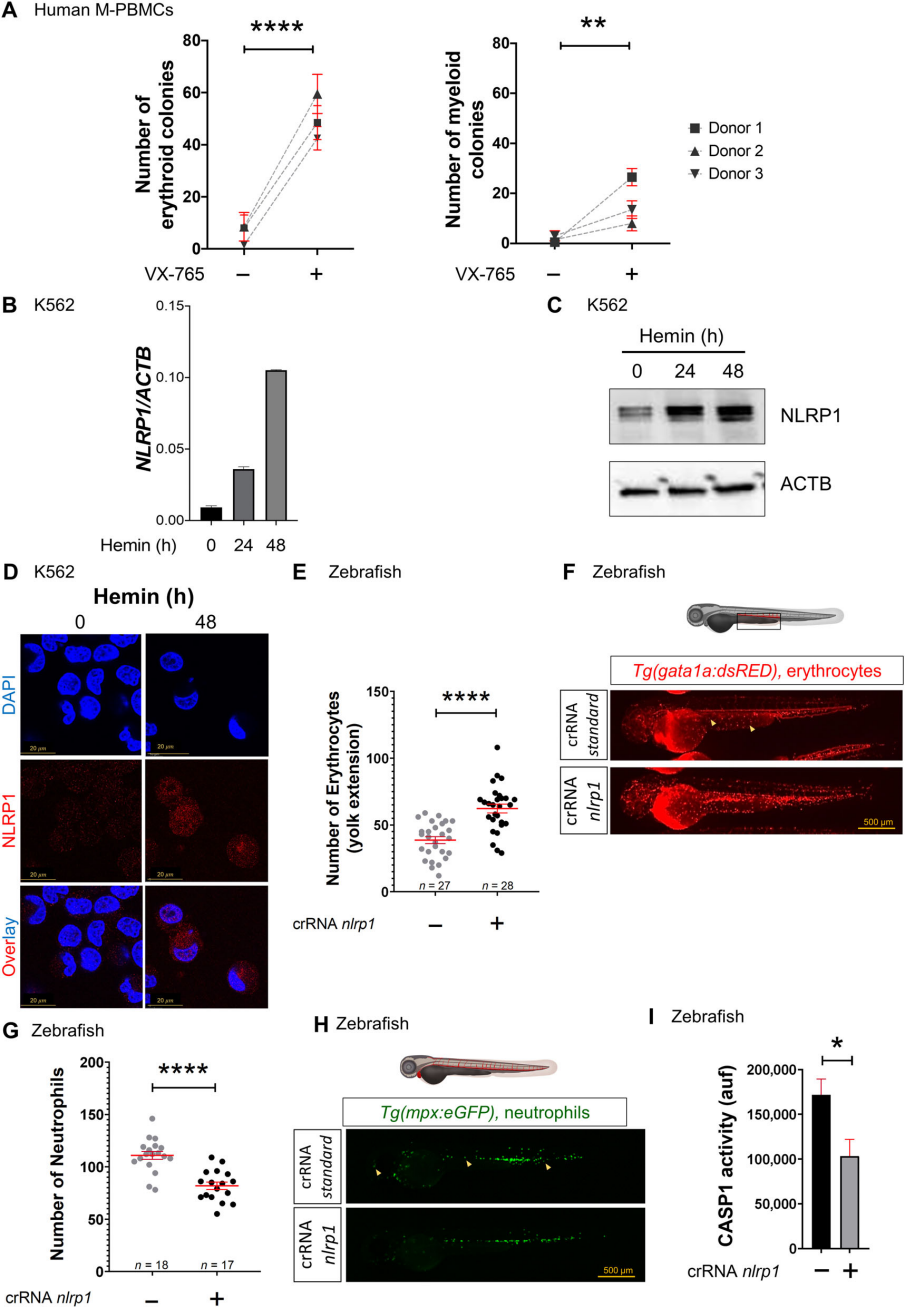
NLRP1 inflammasome regulates hematopoiesis A
Number of erythroid and myeloid colonies derived from M‐PBMCs obtained from healthy donors in the presence of DMSO or 100 μM VX‐765. Each symbol represents a different donor.B–D
Transcript (B) and protein (C, D) amounts of NLRP1 in K562 cells after differentiation with 50 μM hemin, assayed by RT–qPCR (B), Western blot (C) and immunofluorescence (D). Nuclei were counterstained with DAPI in D. Data are shown as the means ± SEM of three technical replicates in B.E–I
Number of erythrocytes (E, F) and neutrophils (G, H), and caspase‐1 activity (I) in *nlrp1* crispant larvae of 2 dpf obtained by injecting one‐cell stage embryos with standard or *nlrp1* crRNAs/Cas9 complexes. Representative images are shown in (F) and (H). Each dot represents one individual and the mean ± SEM for each group is also shown in (E) and (G). Data are shown as the means ± SEM of four technical replicates in I. *P* values were calculated using Student's *t*‐test. *P < 0.05; **P < 0.01; ****P < 0.0001. Source data are available online for this figure.

First, we extended our previous results on the impact of pharmacological inhibition of caspase‐1 in zebrafish and human K562 cell line using mobilized human peripheral blood mononuclear cells (M‐PBMCs). Thus, pharmacological inhibition of CASP1 with VX‐765 (Belnacasan) led to a robust increase in the number of erythroid colonies in M‐PBMCs (Fig 1A). To identify the NLR sensor involved in the regulation of hematopoiesis, we performed a transcriptomic analysis in K562 cells after erythroid differentiation and found that the *NLRP1* gene was strongly upregulated (Appendix Fig S1). This result was confirmed by RT–qPCR, Western blotting and immunofluorescence (Fig 1B–D). Interestingly, NLPR1 was found not only in the nucleus, but also in the cytoplasm of erythroid differentiated K562 cells (Fig 1D), as we had previously found with CASP1 (Tyrkalska *et al*, 2019). In addition, single cell analysis by immunofluorescence revealed a correlation between low amounts of GATA1 and high amounts of CASP1 and NLRP1 after erythroid differentiation (Appendix Fig S2).

These results prompted us to study the impact of the Nlrp1 inflammasome on hematopoiesis using zebrafish transgenic lines *Tg*(*gata1a:dsRed*), *Tg*(*mpx:eGFP*) and *Tg*(*mfap4.1:Tomato*), which have labeled erythrocytes, neutrophils and macrophages, respectively. Although *nlrp1* crispant larvae obtained by CRISPR‐Cas9 technology showed normal development (Appendix Fig S3A and D), they exhibited a significant increase in the number of erythrocytes (Fig 1E and F), a decrease in neutrophil and macrophage counts (Fig 1G and H; Appendix Fig S3E and F), and a reduction in caspase‐1 activity (Fig 1I). Furthermore, *nlrp1* deficiency also alleviated neutrophilia of the Spint1a mutant model of neutrophilic inflammation without affecting skin inflammation or neutrophil skin infiltration (Appendix Fig S3G and H) and thus phenocopying the effects of pharmacological caspase‐1 inhibition in this model (Tyrkalska *et al*, 2019).

### LRRFIP1 and FLII negatively regulate human NLRP1 inflammasome activation

DPP9 has been found to restrain NLRP1 activation in most biological systems (Bauernfried & Hornung, 2022). However, although DPP9 was expressed in K562 cells (Appendix Fig S4A and B), it did not interact with NLRP1 (Appendix Fig S4C), and talabostat, a DPP9 inhibitor commonly used to activate NLRP1 (Zhong *et al*, 2018), did not affect either erythroid differentiation (Appendix Fig S4D) or caspase‐1 activity (Appendix Fig S4E). In addition, we were unable to detect DPP9 or its interaction with NLRP1 in M‐PBMCs (Appendix Fig S4F). Therefore, we immunoprecipitated endogenous NLRP1 in K562 cells after erythroid differentiation and then performed mass spectrometry analysis to identify new interactors of NLRP1 (Appendix Fig S5A–C). We focused our attention on LRRFIP1 and FLII because they were consistently identified in all three biological replicates analyzed (Appendix Fig S5D and E) and were previously reported to interact with each other (Fong & de Couet, 1999; Lee & Stallcup, 2006).

**Figure 2:**
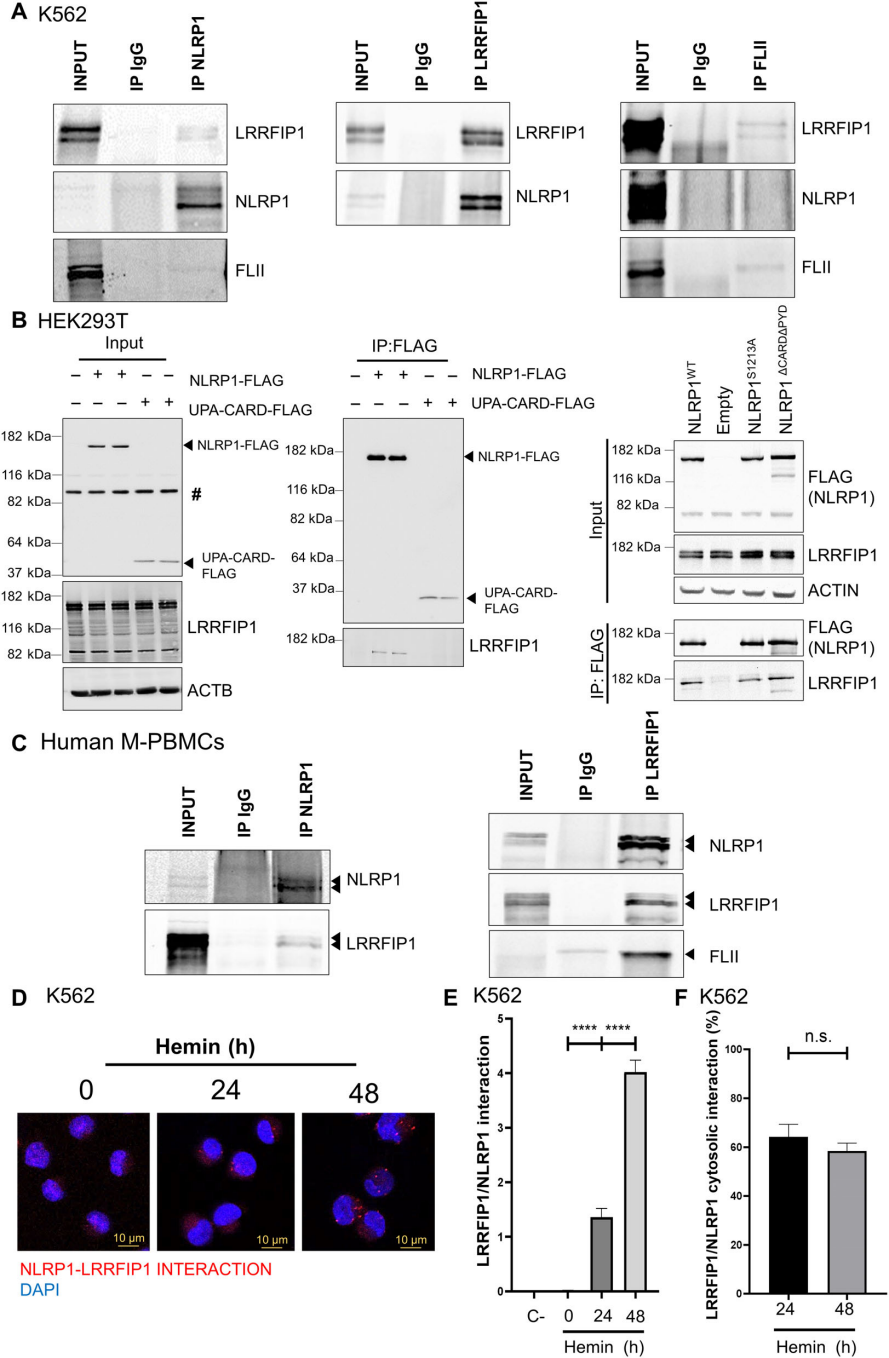
LRRFIP1 and FLII interact with human NLRP1 inflammasome A–F
Interaction of NLRP1 with LRRFIP1 and FLII in K562 cells (A, D–F), HEK293 cells (B) and M‐PBMCs (C) assayed by co‐immunoprecipitation assays and PLA (D–F). Endogenous proteins (A, C–F) and transfected FLAG‐tagged full‐length NLRP1, NLRP1ΔCARDΔPYD, NLRP1_S1213A and UPA‐CARD (B) were analyzed. The number of interactions per cell (E) and the percentage of cytosolic interactions (F) are shown (*n* = 100 cells). Data are shown as the means ± SEM. *P* values were calculated using one‐way ANOVA and Tukey's multiple range test. n.s., non‐significant; ****P < 0.0001. #, non‐specific band. Source data are available online for this figure.

First, we observed that *LRRFIP1* and *FLII* transcript levels increased during erythroid differentiation (Appendix Fig S6A) and were strongly expressed at the protein level, as assayed by Western blot and immunofluorescence (Appendix Fig S6B and C). LRRFIP1 was mainly localized in the cytosol, whereas FLII was mainly found in the nucleus (Appendix Fig S6C). Second, we confirmed a robust interaction between NLRP1 and LRRFIP1 in K562 cells after erythroid differentiation, assayed by immunoprecipitation of either protein (Fig 2A). However, a weaker, but consistent, interaction between endogenous NLRP1 and FLII was observed (Fig 2A). As expected, LRRFIP1 and FLII interacted strongly with each other (Fig 2A). LRRFIP1 was able to interact with full‐length NLRP1 (wild type and autoproteolysis deficient S1213A) and NLRP1‐ΔPYDΔ‐CARD, but not with active UPA‐CARD (Fig 2B and C; Appendix Fig S7A). The interaction of NLRP1 with LRRFIP1 and FLII was also confirmed in M‐PBMCs, where a strong interaction of NLRP1 with both LRRFIP1 and FLII was observed (Fig 2C). More importantly, a proximity ligation assay (PLA) revealed *in vivo* interaction between NLRP1 and LRRFIP1 in the cytosol and nucleus of K562 cells after erythroid differentiation (Fig 2D–F).

**Figure 3:**
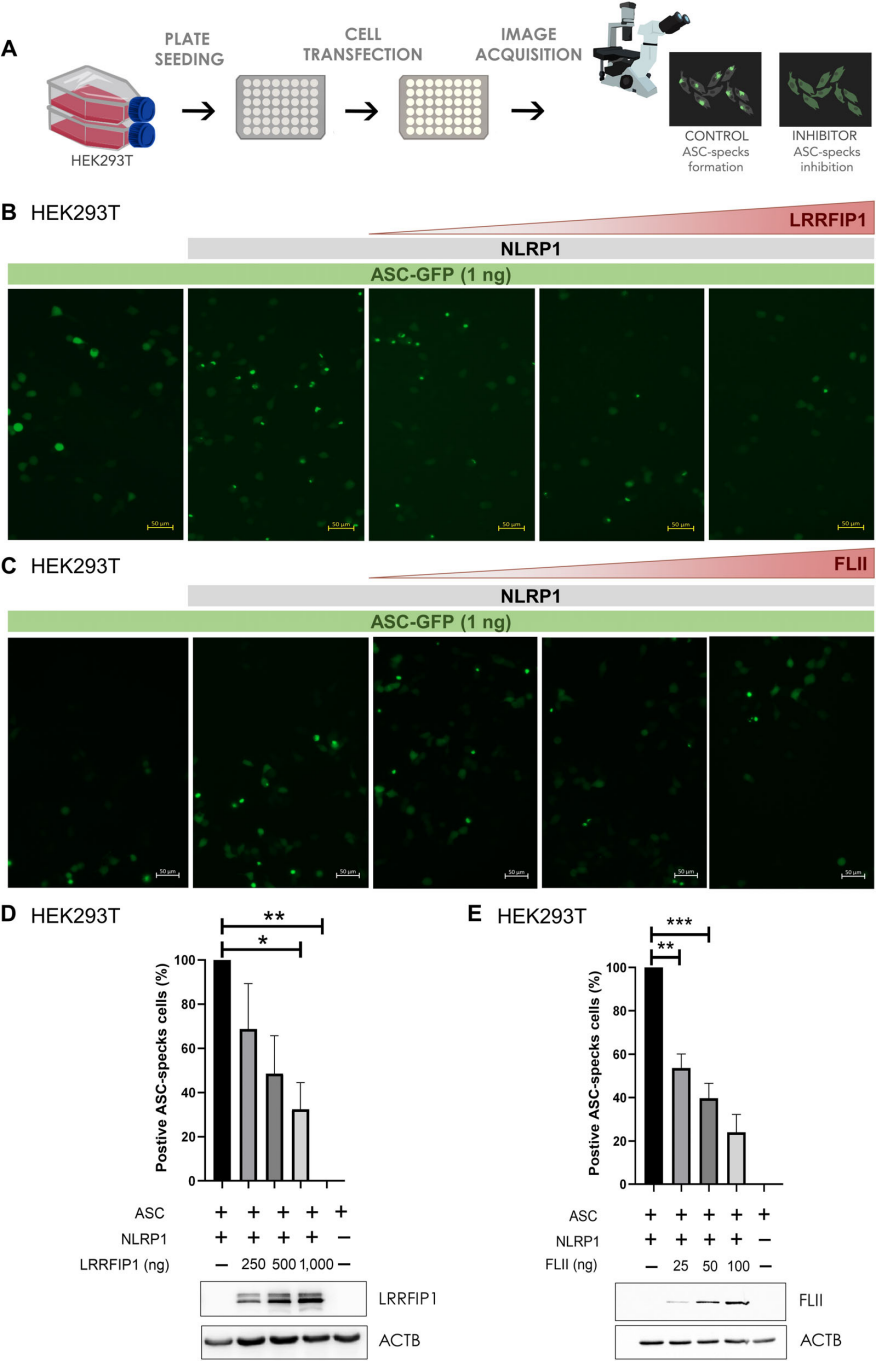
LRRFIP1 and FLII negatively regulate human NLRP1 inflammasome activation A
HEK293T cells were transfected with 300 ng NLRP1‐FLAG, 1 ng ASC‐GFP and the indicated concentrations of FLAG‐LRRFIP1 and FLAG‐FLII plasmids, and the formation of ASC specks was analyzed by fluorescence microscopy 24 h post‐transfection.B, C
Representative images of ASC specks.D, E
Number of positive ASC specks in cells co‐transfected with LRRFIP1 (D) and FLII (E). Expression of LRRFIP and FLII was confirmed by Western blot using anti‐FLAG and anti‐ACTB antibodies. Data are shown as the means ± SEM (*n* = 95–156 cells). *P* values were calculated using one‐way ANOVA and Tukey's multiple range test. *P < 0.05; **P < 0.01; ***P < 0.001. Source data are available online for this figure.

These results prompted us to investigate the role of LRRFIP1 and FLII in NLRP1 activation by reconstituting this inflammasome in HEK239T and evaluating the formation of ASC specks using small amounts of ASC‐GFP to prevent its self‐oligomerization (Fig 3A). The results showed that both LRRFIP1 (Fig 3B and D) and FLII (Fig 3C and E) were able to reduce ASC speck formation in a dose‐dependent manner. However, neither LRRFIP1 nor FLII inhibited the self‐oligomerization of ASC (Appendix Fig S8A–D) or the activation of NLRP3 inflammasome (Appendix Fig S9A and B).

### Lrrfip1 and Flii regulate hematopoiesis through the Nlrp1 inflammasome in zebrafish

**Figure 4:**
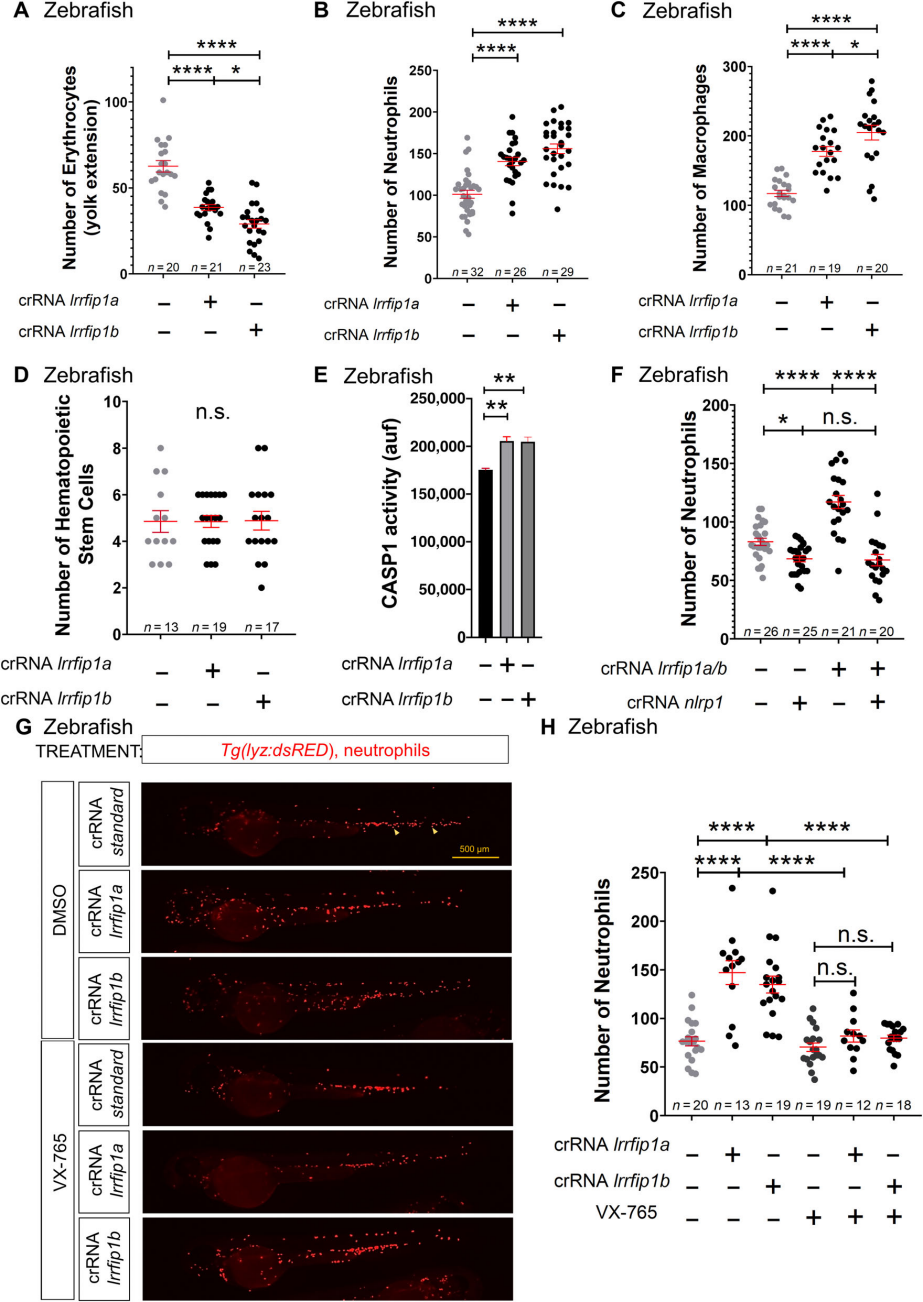
Lrrfip1 regulates hematopoiesis through the Nlrp1 inflammasome in zebrafish A–H
Number of erythrocytes (A), neutrophils (B, F, H), macrophages (C), and HSPCs (D), and caspase‐1 activity (E) in *nlrp1* crispant larvae of 2 dpf obtained by injecting one‐cell stage embryos with standard, *nlrp1*, *lrrfip1a* and/or *lrrfip1b* crRNAs/Cas9 complexes. Note that either *lrrfip1a* or *lrrfip1b* crRNAs/Cas9 complexes target both *lrrfip1* paralogs (see Appendix Fig S10). (G, H) Larvae were also treated by bath immersion with 100 μM of the caspase‐1 inhibitor VX‐765. Representative images of neutrophils in *lrrfip1* crispant larvae using *Tg*(*lyz:dsRED*) reporter line are shown in (G). Each dot represents one individual and the means ± SEM for each group are also shown. *P* values were calculated by Student's *t*‐test. n.s., non‐significant; *P<0.05; **P<0.01; ****P<0.0001. Source data are available online for this figure.

We next sought to determine whether Lrrfip1 and Flii are involved in the regulation of hematopoiesis using the zebrafish model. Zebrafish have two paralog genes of *lrrfip1*, called *lrrfip1a* and *lrrfip1b*, which showed high homology to each other (62% nucleotide identity). We designed crRNAs for both genes and found that each crRNA edited both genes with different efficiencies: the crRNA for *lrrfip1a* showed a knockdown efficiency of 20% for *lrrfip1a* and *lrrfip1b* (Appendix Fig S10A and B), whereas the crRNA for *lrrfip1b* edited *lrrfip1a* and *lrrfip1b* with 65 and 80% efficiency, respectively (Appendix Fig S10C and D). Although *lrrfip1a* and *lrrfip1b* crispant larvae developed normally without any obvious developmental defects (Appendix Fig S10E–G), these editing efficiencies translated into hematopoietic alterations. Thus, *lrrfip1a* and *lrrfip1b* crispant larvae showed fewer erythrocytes, increased counts of neutrophils and macrophage, an unaltered number of HSPCs, and higher caspase‐1 activity than wild type larvae (Fig 4A–E; Appendix Fig S11A–D). Strikingly, the increased number of neutrophils of *lrrfip1a/b* crispant larvae was rescued by *nlrp1* deficiency (Fig 4F; Appendix Fig S11E) and the caspase‐1 inhibitor VX‐765 (Fig 4G and H). Taken together, all these results suggest that Lrrfip1 acted upstream Nlrp1, as a negative regulator, and critically regulated hematopoiesis.

**Figure 5:**
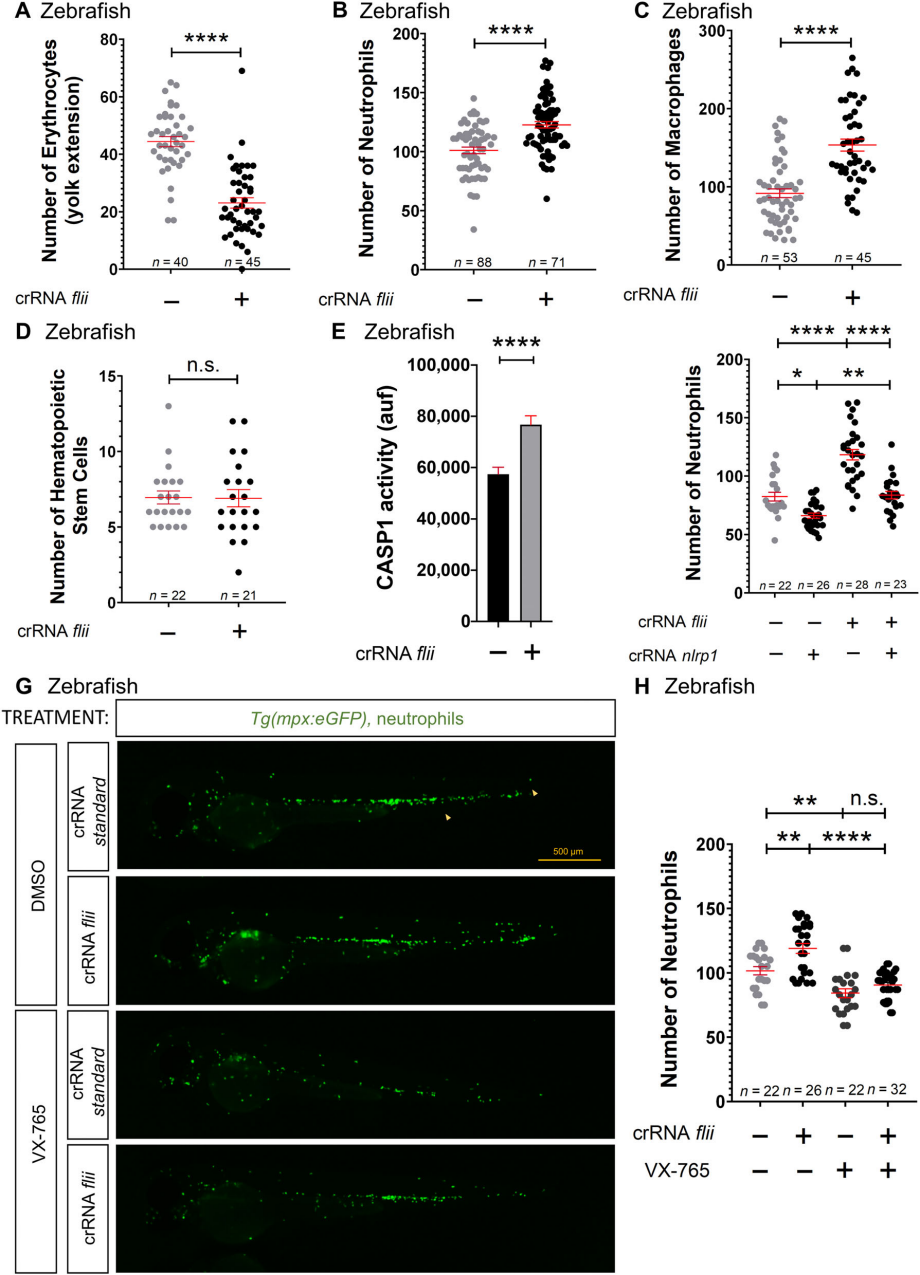
Flii regulates hematopoiesis through the Nlrp1 inflammasome in zebrafish A–H
Number of erythrocytes (A), neutrophils (B, F‐H), macrophages (C), and HSPCs (D), and caspase‐1 activity (E) in *nlrp1* crispant larvae of 2 dpf obtained by injecting one‐cell stage embryos with standard, *nlrp1* and/or *flii* crRNAs/Cas9 complexes. (G, H) Larvae were also treated by bath immersion with 100 μM of the caspase‐1 inhibitor VX‐765. Representative images of neutrophils in *flii* crispant larvae using *Tg*(*mpx:eGFP*) reporter line are shown in (G). Each dot represents one individual and the mean ± SEM for each group is also shown. *P* values were calculated using Student's *t*‐test. n.s., non‐significant; *P < 0.05; **P < 0.01; ****P < 0.0001. Source data are available online for this figure.

Similarly, Flii knockdown (80% efficiency) (Appendix Fig S12A) produced no obvious developmental alteration (Appendix Fig S12B–D), but phenocopied the hematopoietic alterations observed in *lrrfi1a/b* crispant larvae (Fig 5A–E; Appendix Fig S13A–D) and the effect on caspase‐1 activity (Fig 5G and H), and were also dependent on Nlrp1 (Fig 5F; Appendix Fig S13E). In contrast, forced expression of *flii* mRNA resulted in increased erythrocyte numbers, reduced neutrophil and macrophages counts (Appendix Fig S14A–F), and reduced caspase‐1 activity (Appendix Fig S14G). Notably, epistasis experiments revealed that forced expression of *flii* was unable to reduce neutrophil numbers in *lrrfip1a/b* crispant larvae, suggesting that the ability of Flii to inhibit the Nlrp1 inflammasome is dependent on Lrrfip1 (Appendix Fig S14H and I). Finally, either Flii (Appendix Fig S15A and B) or *lrrfip1a/b* (Appendix Fig S15C and D) deficiency exacerbated the neutrophilia of the Spint1a mutant inflammation model.

### ZAKα activates the NLRP1 inflammasome in HSPCs after ribosomal stress in zebrafish and human

**Figure 6:**
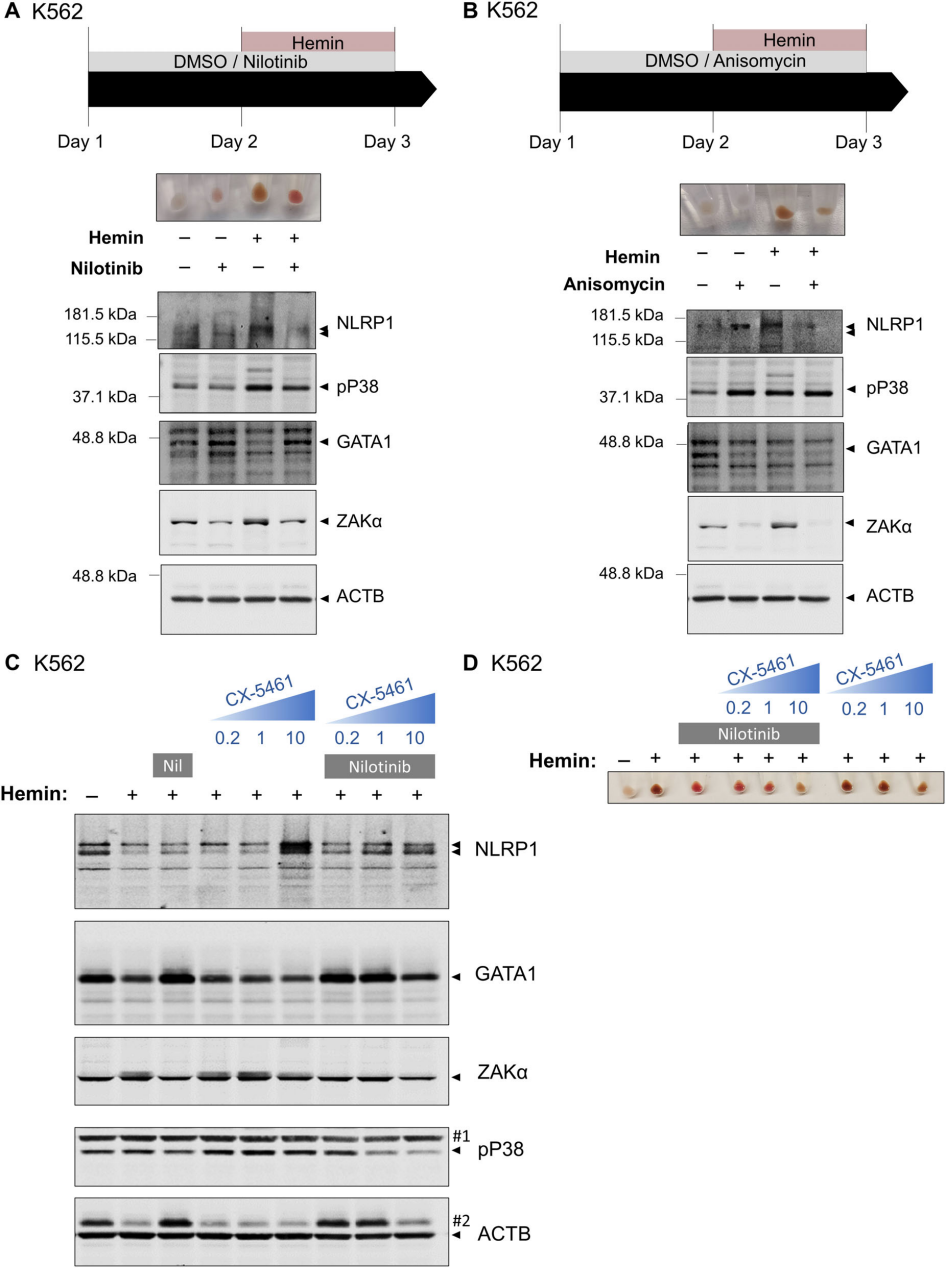
ZAKα/P38 signaling pathway is activated in K562 after erythroid differentiation and its inhibition facilitates terminal erythroid differentiation A–D
K562 cells were pretreated with 0.1 μM nilotinib (Nil) (A, C, D), 1 μM anisomycin (B) and/or the indicated concentrations of the RNA Pol II inhibitor CX‐5461 for 24 h, and then differentiated with 50 μM hemin for another 24 h. Hemoglobin accumulation (A, B, D) and NLRP1, phosphorylated P38, GATA1, ZAKα and ACTB (A–C) amounts were then evaluated by Western blot. Immunoblots are representative of three independent experiments. #1, ACTB; #2, GATA1. Source data are available online for this figure.

**Figure 7:**
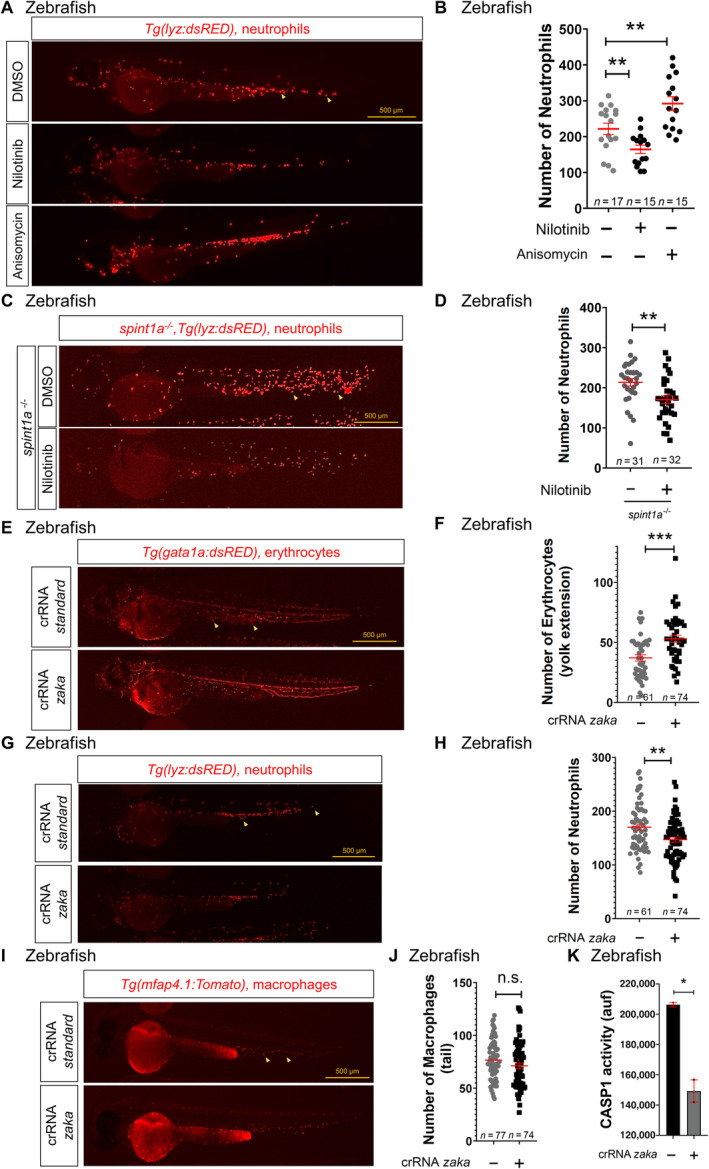
ZAKα regulates hematopoiesis in zebrafish through the Nlrp1 inflammasome A–K
Number of neutrophils (B, D, H), erythrocytes (F) and macrophages (J), and caspase‐1 activity (K) in 2 dpf larvae either treated from 1 to 2 dpf by bath immersion with 1 μM nilotinib and/or 100 μM anisomycin (A–D) or obtained by injecting one‐cell stage embryos with standard or *zaka* crRNAs/Cas9 complexes (E–K). Representative images of neutrophils (A, C, G), erythrocytes (E) and macrophages (I) are also shown. Each dot represents one individual and the mean ± SEM for each group is also shown. *P* values were calculated using one‐way ANOVA and Tukey's multiple range test (B) or Student's *t*‐test (D, F, H, J, K). Data are shown as the means ± SEM of two technical replicates in (K). n.s., non‐significant; *P < 0.05; **P < 0.01; ***P < 0.001. Source data are available online for this figure.

Given that our results suggest that DPP9 does not regulate the NLRP1 inflammasome in HSPCs and that three recent papers have shown that cellular stress, including oxidative nucleic acid damage and ribotoxic stress, activates ZAKa/P38 axis that phosphorylates and activates human NLRP1 inflammasome independently of DPP9 (Robinson *et al*, 2022; Jenster *et al*, 2023; Zhou *et al*, 2023), we tested whether a similar mechanism operates to regulate hematopoiesis. In K562 cells, nilotinib, a kinase inhibitor that has a high affinity for ZAKα (Sauter *et al*, 2010), strongly promoted erythroid differentiation, even in the absence of hemin (Fig 6A). Notably, erythroid differentiation with hemin resulted in phosphorylation of P38 and ZAKα, and degradation of GATA1, whereas nilotinib strongly reversed all of them (Fig 6A; Appendix Fig S16). Furthermore, nilotinib facilitated erythroid differentiation of K562 cells treated with the antibiotic anisomycin (Fig 6B), which promotes ribotoxic stress‐mediated activation of NLRP1 (Jenster *et al*, 2023). In addition, nilotinib was also able to promote accumulation of GATA1 in the presence of the RNA polymerase I inhibitor CX‐5461, which impaired erythroid differentiation of K562 cells (Fig 6C and D) and is widely used to model ribosomopathies, such as Diamond‐Blackfan anemia (DBA) (Khajuria *et al*, 2018). Similarly, treatment of zebrafish larvae with nilotinib caused a decrease in neutrophil number in wild type larvae and reduced neutrophilia in the Spint1a mutant model of neutrophilic inflammation (Fig 7A–D), whereas anisomycin caused neutropenia (Fig 7A and B).

We next investigated whether the effects of nilotinib and anisomycin in hematopoiesis were mediated through ZAKα. Interestingly, although human ZAKα and ZAKβ are generated by alternative splicing from the same gene and only ZAKα can interact with the ribosome (Vind *et al*, 2020), zebrafish showed two genes: *map3k20a* encoding Zaka and *map3k20b* encoding Zakb (Appendix Fig S17A). Although knockdown of Zaka and Zakb (60 and 80% editing efficiency, respectively) did not result in any obvious developmental defects (Appendix Fig S17B–E), *zaka* deficiency phenocopied the effects of *nlrp1* deficiency in zebrafish larvae; that is, reduced erythrocyte numbers, increased neutrophil and macrophage counts (Fig 7C–J), and decreased caspase‐1 activity (Fig 7K). In sharp contrast, *zakb* deficiency did not affect neutrophil numbers (Appendix Fig S18A and B). Importantly, the increase in neutrophil numbers observed in *flii* crispant larvae was fully rescued by *zaka* deficiency (Appendix Fig S18C and D) and anisomycin failed to increase neutrophil number in *nlrp1*‐ and *zaka* crispant larvae (Appendix Fig S18E and F), suggesting that Zaka acts upstream of Nlrp1 activation.

**Figure 8:**
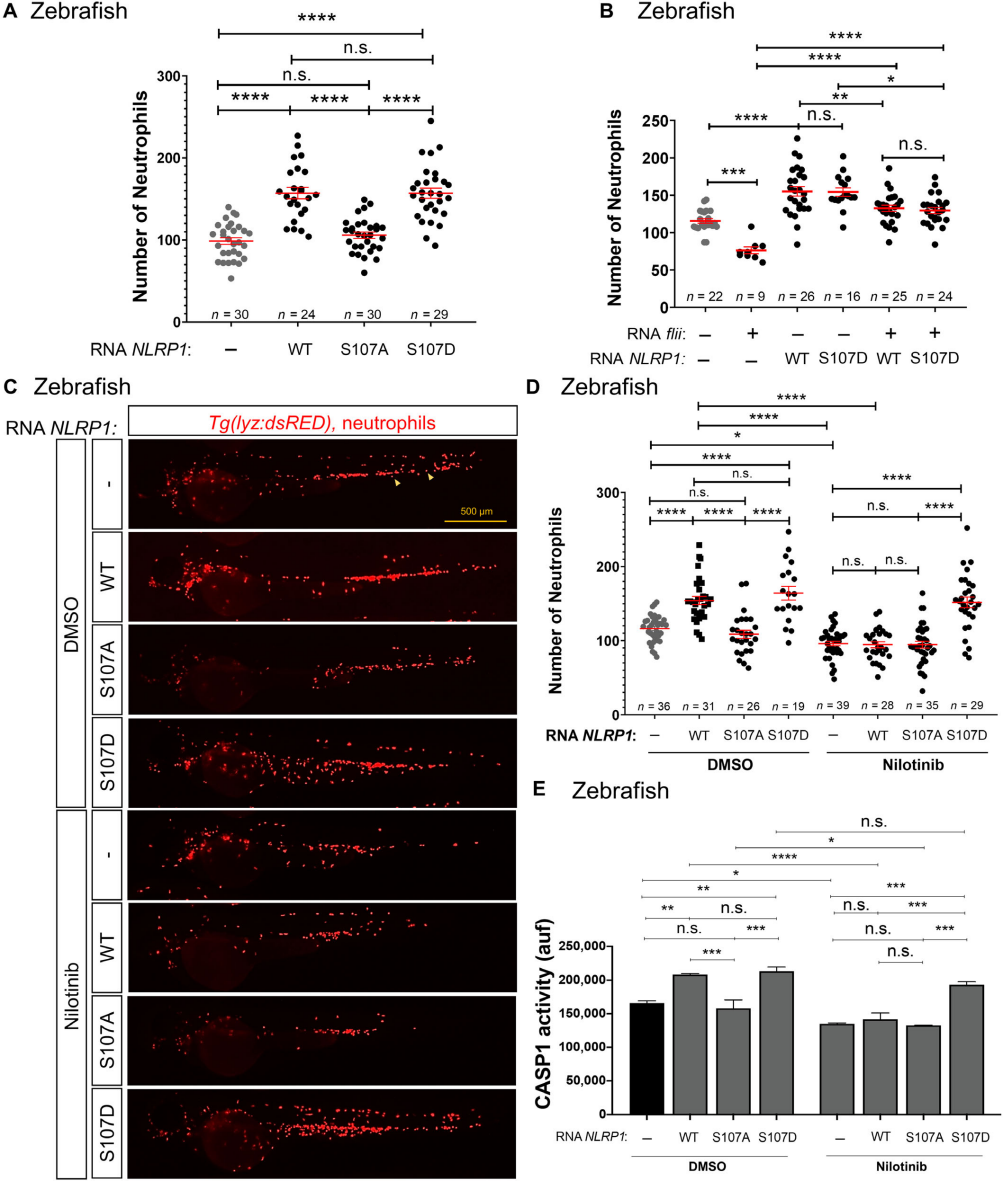
NLRP1 is activated by phosphorylation of the linker domain after activation of ZAKα A–E
Number of neutrophils (A–D), and caspase‐1 activity (E) in 2 dpf larvae obtained by injection of one‐cell stage embryos with human NLRP1 (wild type‐WT, S107A and S107D) and treated from 1 to 2 dpf by bath immersion with 1 μM nilotinib (D, E). Representative images of neutrophils (arrows) are also shown in (C). Each dot represents one individual and the mean ± SEM for each group is also shown. Data are shown as the means ± SEM of two technical replicates in (E). *P* values were calculated by one‐way ANOVA and Tukey's multiple range test. n.s., non‐significant; *P < 0.05; **P < 0.01; ***P < 0.001; ****P < 0.0001. Source data are available online for this figure.

The conservation of the activation of zebrafish and human NLRP1 by ZAKα following cellular stress was unexpected, as mouse NLRP1s are not activated by ribotoxic stress and both mouse and zebrafish lack a C‐terminal PYD domain (Jenster *et al*, 2023). However, the linker domain of zebrafish Nlrp1 (Appendix Fig S17F) showed relatively well conserved serine and threonine residues, including S107, which has been shown to be directly phosphorylated by P38 and important for human NLRP1 activation by this mechanism (Jenster *et al*, 2023). To confirm the conservation of this activation mechanism in zebrafish and gain further insight into the relevance of NLRP1 phosphorylation by ZAKα, we expressed human wild type S107A and S107D NLRP1 in zebrafish larvae and found that both wild type and S107D, but not S107A, increased neutrophil number (Fig 8A) and caspase‐1 activity (Fig 8E). Furthermore, the neutropenia of larvae induced by forced expression of zebrafish *flii* was rescued by human wild type and S107D NLRP1 (Fig 8B). Strikingly, although nilotinib was able to abrogate neutrophilia and caspase‐1 activation induced by wild type NLRP1, it failed to reverse neutrophilia and caspase‐1 activation induced by NLRP1 with phosphomimetic S107D mutation (Fig 8C–E). These results confirmed that the activation of NLRP1 inflammasome is conserved in zebrafish and human and that NLRP1 is activated by phosphorylation of the linker domain following activation of ZAKα in HSPCs to regulate the erythroid‐myeloid lineage decision.

## Discussion

**Figure 9:**
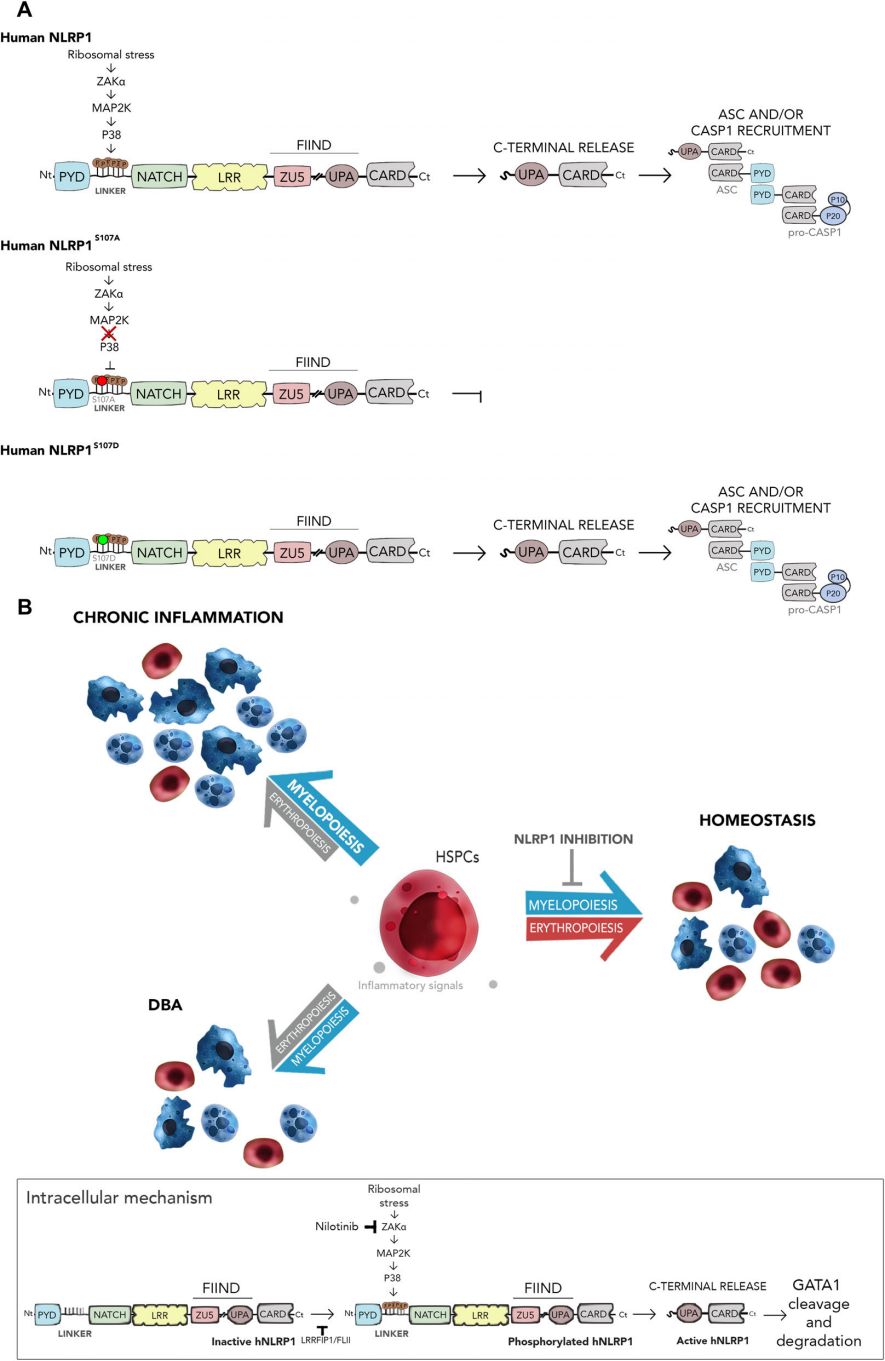
Activation of NLRP1 by the ZAKα/P38 signaling pathway A
Schemes showing the domain organization of human wild type NLRP1 and the S107A and S107D mutants. Phosphorylation of the linker domain by ZAKα (MAP3K)/MAP2K/P38 (MAPK) axis is highlighted. Note that S107A cannot be phosphorylated by P38, while phosphomimetic S107D is constitutively active independently of P38 activation.B
The NLRP1 inflammasome critically regulates hematopoiesis following its activation by ZAKα/P38 signaling pathways, which is initiated by ribosomal stress. Inhibition of this pathway with nilotinib rescues erythropoiesis defects in HSPCs from DBA and neutrophilic inflammation patients.

Although the relevance of inflammasomes in the regulation of inflammation and immunity is widely known, their role in hematopoiesis is just beginning to be appreciated (Rodriguez‐Ruiz *et al*, 2020; Ratajczak & Kucia, 2021). For example, the NLPR3 inflammasome has been found to regulate HSPC trafficking, but its excessive activation can lead to HSPC death by pyroptosis and the onset of myelodysplastic syndromes and other hematological malignancies (Ratajczak & Kucia, 2021). Similarly, a gain‐of‐function mutation of murine NLRP1a induces IL‐1β‐driven neutrophilia and systemic inflammation but, paradoxically, in the absence of IL‐1β signaling, reduces the number of myeloid progenitors (Masters *et al*, 2012), suggesting that systemic inflammation unmasks myeloid progenitor pyroptosis. In addition, chemotherapy and viral infection further exacerbated HSPC death by pyroptosis (Masters *et al*, 2012). In the present study, however, we show that the NLRP1 inflammasome plays a previously unappreciated physiological role in regulating erythroid‐myeloid lineage decision in both zebrafish and human HSPCs (Fig 9). Thus, genetic inhibition of Nlrp1 led to an increase in erythrocyte numbers in zebrafish larvae and a concomitant reduction in neutrophil and macrophage counts, phenocopying the effects of pharmacological inhibition of caspase‐1 or genetic inhibition of Asc or Guanylate‐binding protein 4 (Gbp4) (Tyrkalska *et al*, 2019).

One of the most interesting results of this study is that zebrafish Nlrp1, despite lacking a C‐terminal PYD domain, like murine NLRP1s, displayed a well‐conserved linker domain, including S107, which has been found to be critical for the activation of human NLRP1 following its phosphorylation by P38 kinase, which is activated by ZAKα in response to ribotoxic stress or alphavirus infection (Jenster *et al*, 2023). In fact, human NLRP1 promoted myelopoiesis in zebrafish larvae and the S107A mutant did not. Furthermore, genetic inhibition of Zaka or treatment of larvae with the tyrosine kinase inhibitor nilotinib also promoted neutropenia and increased erythrocyte counts in zebrafish. Interestingly, the human phosphomimetic S107D NLRP1 mutant was able to promote myelopoiesis in the presence of the tyrosine kinase inhibitor nilotinib, demonstrating conservation of the activation of NLRP1 by ZAKα/P38‐mediated phosphorylation of S107 between zebrafish and humans, in contrast to mice (Robinson *et al*, 2022; Jenster *et al*, 2023), and pointing to the relevance of the zebrafish model for studying the regulation of hematopoiesis by the inflammasome (Fig 9A and B). In particular, zebrafish would be a uniquely appropriate model to study the contribution of Zaka in the activation of the Nlrp1 inflammasome and the role of Zakb, since zebrafish have *zaka* and *zakb* genes, unlike mice and humans, where ZAKα and ZAKβ are generated by alternative splicing from the same gene.

The activation of ZAKα in HSPCs after their differentiation is surprising. However, ZAKα is not only activated by the ribotoxic stress response, but also by viral infection (Jenster *et al*, 2023) and oxidative nucleic acid damage and cellular stress (Zhou *et al*, 2023). We observed that the ZAKα‐P38 axis is activated in K562 cells after erythroid differentiation and inhibition of this axis with nilotinib promoted GATA1 accumulation and its erythroid differentiation even in the absence of hemin. Similarly, it has been shown that ribosome biogenesis is abruptly interrupted during human and murine erythropoiesis, leading to activation of the ATR/CHK1/P53 pathway (Le Goff *et al*, 2021). We propose, therefore, that ribosomal stress induced during erythroid differentiation also activates the ZAKα/P38 signaling pathway, which, in turn, induces NLRP1 inflammasome activation, GATA1 cleavage, and terminal erythroid differentiation (Tyrkalska *et al*, 2019).

DPP9 has been widely reported as the main negative regulator of mouse and human NLRP1 (Bauernfried & Hornung, 2022). However, recent studies have found that activation of human NLRP1 by both ZAKα/P38 axis following ribotoxic stress response and viral infection (Robinson *et al*, 2022; Jenster *et al*, 2023) and by the ORF45 protein of the Kaposi's sarcoma‐associated herpesvirus (Yang *et al*, 2022) are independent of DPP9. Interestingly, in both cases, activation required the linker domain and thus murine NLRP1s cannot be activated by these stimuli. In the present study, we have identified that the regulation of the NLRP1 inflammasome in HSPCs is also independent of DPP9 and found LRRFIP1 and FLII as two negative regulators of NLRP1 that are critically involved in hematopoiesis. LRRFIP1 and FLII are pleiotropic proteins capable of interacting with each other and regulating different biological processes, including regulation of transcription, cell cycle and apoptosis, cytoskeleton remodeling, signal transduction, and immunity (Takimoto, 2019; Strudwick & Cowin, 2020). FLII has also been found to interact with pro‐caspase‐1 and pro‐caspase‐11 by dampening their activation (Li *et al*, 2008). In addition, LRRFIP2, which shows 41% sequence homology with LRRFIP1, has also been found to directly interact and inhibit the activation of macrophage NLRP3 inflammasome by recruiting FLII and facilitating the inhibition of caspase‐1 (Jin *et al*, 2013). Interestingly, LRRFIP1 fails to bind and inhibit NLRP3 inflammasome (Jin *et al*, 2013). Furthermore, the signaling adaptor B cell adaptor for phosphoinositide 3‐kinase (BCAP) has also been reported to interact with LRRFIP1 and FLII to inhibit NLRP3 and NLRC4 inflammasomes (Carpentier *et al*, 2019). We found that LRRFIP1 bound full‐length NLRP1, but not UPA‐CARD, and inhibited it. In addition, we confirmed that LRRFIP1 was unable to inhibit NLRP3. Notably, epistasis experiments suggest that the ability of Flii to inhibit the Nlrp1 inflammasome in zebrafish is dependent on Lrrfip1, as it has been demonstrated for the binding of FLII to NLRP3, which requires LRRFIP2 (Jin *et al*, 2013). Therefore, we have identified a novel mechanism involved in the negative regulation of the NLRP1 inflammasome involving recruitment of FLII by LRRFIP1 to inhibit the NLRP1 inflammasome in HSPCs. It would be interesting to address whether BCAP is required for the negative regulation of the NLRP1 inflammasome of HSPCs by LRRFIP1/FLII and the relevance of LRRFIP1 and FLII in the regulation of NLRP1 inflammasome in other cell types, such as keratinocytes, especially after their activation by UV‐induced ribotoxic stress response and viral infection, which are independent of DPP9. Furthermore, although it is expected that LRRFIP1 and FLII inhibit caspase‐1 downstream NLRP1 inflammasome activation, as FLII is a caspase‐1 pseudosubstrate (Jin *et al*, 2013), it would be compelling to ascertain whether LRRFIP1 and FLII regulate the phosphorylation of NLRP1 by ZAKα/P38 tyrosine kinase axis.

The results obtained in this study may also have clinical implications, since hematopoietic lineage bias, such as neutrophilia and anemia, is often associated with chronic inflammatory diseases (Weiss, 2015; Marzano *et al*, 2018), cancer (Wu *et al*, 2014) and aging (Elias *et al*, 2017). Furthermore, the ZAKα/P38 signaling pathway is expected to be hyperactivated in ribosomopathies, such as DBA (Fig 9A and B). In fact, increased phosphorylation of P38 has been found in human RPS19‐deficient erythroid progenitors and pharmacological inhibition of P38 in CD34^+^ cells increased GATA1 levels (Bibikova *et al*, 2014). Our results strongly support this idea, since genetic inhibition of Zaka or Nlpr1, forced expression of the endogenous Nlrp1 inhibitor Flii, and pharmacological inhibition of Zaka with nilotinib alleviated neutrophilia in a zebrafish model of neutrophilic inflammation. In addition, nilotinib also promoted erythroid differentiation and GATA1 accumulation in K562 cells upon inhibition of RNA polymerase I. Since nilotinib (Tasigna) is an FDA/EMA‐approved drug for the treatment of chronic myeloid leukemia, it is attractive for repurposing in the treatment of DBA and other ribosomopathies.

In summary, we have identified that the NLRP1 inflammasome critically regulates hematopoiesis following its activation by ZAKα/P38 signaling pathways, which is initiated by ribosomal stress, and that LRRFIP1 and FLII negatively regulate this activation, rather than DPP9. Since this signaling pathway is conserved between zebrafish and humans, but not mice, zebrafish is a unique model to further clarify the relevance of the NLRP1 inflammasome in hematopoiesis. In addition, our study has identified novel therapeutic targets for the treatment of hematopoietic alterations associated with chronic and rare diseases.

## Materials and Methods

### Zebrafish

Zebrafish (*Danio rerio* H.) were obtained from the Zebrafish International Resource Center and mated, staged, raised, and processed as described (Westerfield, 2000). The lines *Tg*(*mpx:eGFP*)^*i114*^ (Renshaw *et al*, 2006), *Tg*(*lyz:DsRED2*)^*nz50*^ (Hall *et al*, 2007), *Tg*(*mfap4.1:Tomato*)^*xt2*^ (Walton *et al*, 2015), *Tg*(*gata1a:DsRed*)^*sd2*^ (Traver *et al*, 2003), *Tg*(*runx1:GAL4*)^*utn6*^ (Tamplin *et al*, 2015), *Tg*(*UAS‐E1B:NTR‐mCherry*) (Davison *et al*, 2007) and casper (*mitfa* ^*w2/w2*^; *mpv17* ^*a9/a9*^) (White *et al*, 2008) were previously described. *spint1a* ^*hi2217Tg/hi2217Tg*^ was isolated from insertional mutagenesis screens (Amsterdam *et al*, 1999). The experiments performed comply with the Guidelines of the European Union Council (Directive 2010/63/EU) and the Spanish RD 53/2013. The experiments and procedures performed were approved by the Bioethical Committees of the University of Murcia (approval number #669/2020).

### CRISPR and RNA injections in zebrafish

Negative control crRNA (catalog no. 1072544, crSTD) and crRNA for *nlrp1*, *flii*, *lrrpif1a*, *lrrfip1b*, *zaka* and *zakb* (Appendix Table S1), and tracrRNA were resuspended in Nuclease‐Free Duplex Buffer to 100 μM. One μl of each was mixed and incubated for 5 min at 95°C for duplexing. After removal from heat and cooling to room temperature, 1.43 μl of Nuclease‐Free Duplex Buffer was added to the duplex, yielding a final concentration of 1,000 ng/μl. Finally, the injection mix was prepared by mixing 1 μl of duplex, 2.55 μl of Nuclease‐Free Duplex Buffer, 0.25 μl Cas9 Nuclease V3 (IDT, 1081058) and 0.25 μl of phenol red, giving final concentrations of 250 ng/μl of crRNA duplex and 500 ng/μl of Cas9. The prepared mix was microinjected into the yolk sac of one‐ to eight‐cell‐stage embryos using a microinjector (Narishige) (0.5–1 nl per embryo). The same amounts of crRNA were used in all experimental groups. The efficiency of each crRNA was tested by amplifying the target sequence with a specific pair of primers (Appendix Table S2) and the amplicon was then analyzed with the TIDE webtool (https://tide.nki.nl/).

The coding sequence of zebrafish *flii* (accession number NM_001257146.1) and human *NLRP1‐FLAG* (accession number NM_033004.4) (wild type, S107A and S107D) were synthesized by GeneScript. *In vitro*‐transcribed RNA was obtained following the manufacturer's instructions (mMESSAGE mMACHINE kit, Ambion). RNA was mixed with microinjection buffer and microinjected into the yolk sac of one‐cell‐stage embryos using a microinjector (Narishige; 0.5–1 nl per embryo). The same amount of RNA was used for all experimental groups.

### Chemical treatments of zebrafish larvae

One dpf larvae were manually dechorionated and treated for 24 h at 28°C by bath immersion with the caspase‐1 inhibitor VX‐765 (Belnacasan, 100 μM), the tyrosine kinase inhibitor nilotinib (AMN107, 1 μM) and the protein synthesis inhibitor anisomycin (100 μM) (all from MedChemExpress) diluted in egg water supplemented with 0.1% DMSO.

### Caspase‐1 activity assays

Caspase‐1 activity was determined with the fluorometric substrate Z‐YVAD 7‐Amido‐4‐trifluoromethylcoumarin (Z‐YVAD‐AFC, caspase‐1 substrate VI, Calbiochem), as previously described (Lopez‐Castejon *et al*, 2008; Angosto *et al*, 2012; Tyrkalska *et al*, 2016). Briefly, 25–35 larvae were lysed in hypotonic cell lysis buffer on ice for 10 min. For each reaction, 100 μg of protein were incubated for 90 min at room temperature with 50 μM Z‐YVAD‐AFC and 50 μl of reaction buffer. After incubation, the fluorescence of AFC released from the Z‐YVAD‐AFC substrate was measured with a FLUOstart spectrofluorometer (BGM, LabTechnologies) at an excitation wavelength of 405 nm and an emission wavelength of 492 nm. A representative graph of caspase‐1 activity of three replicates is shown in the figures.

### Imaging of zebrafish larvae

Larvae were anesthetized in embryo medium with 0.16 mg/ml buffered tricaine and whole‐body images were taken with a Leica MZ16F fluorescence stereomicroscope. The number of neutrophils (*mpx* ^+^ or *lyz* ^+^), macrophages (*mfap4.1* ^+^), erythrocytes (*gata1a* ^+^, in the yolk sac extension) and HSPCs (*runx1* ^+^) was determined by counting them visually in blinded samples (Tyrkalska *et al*, 2019).

### Primary cell collection and culture

M‐PBMCs were obtained from several healthy donors provided by the Hematology Service at Hospital Clínico Universitario Virgen de la Arrixaca (HCUVA) with ethics approval number 2021‐11‐9‐HCUVA. Briefly, donors were treated with 10 μg/Kg G‐CSF (Filgrastim, split in 2 doses/day for 5 days) and PBMCs were collected by apheresis. The percentage of CD34^+^ cells were analyzed by flow cytometry and ranged from 0.9 to 1.6. Ten thousand M‐PBMCs were seeded in 6‐well plates, incubated in human methylcellulose complete medium (#HSC003, R&D Systems) at 37°C in a 5% CO_2_‐humidified atmosphere, and colonies were counted at day 9 and 14 using standard morphological criteria.

### Erythroid differentiation assay

K562 cells (CRL‐3343; American Type Culture Collection) were maintained in RPMI culture medium supplemented with 10% fetal calf serum (FCS), 2 mM Glutamine, and 1% penicillin–streptomycin (Life Technologies). They were tested for mycoplasma contamination and authenticated by STR profiling. Cells were maintained and subcultured before confluence every 72 h. For differentiation, cells were treated with 50 μM hemin (#16009‐13‐5, Sigma‐Aldrich), prepared as previously described (Smith *et al*, 2000), in the presence of 0.1% DMSO alone or with the caspase‐1 inhibitor VX‐765 (100 μM), the tyrosine kinase inhibitor nilotinib (0.1 μM), the RNA polymerase I inhibitor CX‐5461 (200 nM, 1 μM and 10 μM) (all from MedChemExpress). Cells were collected at different time points (0, 24, 48 h post‐hemin addition), centrifuged, washed twice with PBS, and stored at −80°C for further analysis.

### Inflammasome reconstitution in HEK293T cells

HEK293T cells (CRL‐11268; American Type Culture Collection) were maintained in DMEM:F12 (1:1) supplemented with 10% FCS, 2 mM Glutamax, and 1% penicillin–streptomycin (ThermoFisher Scientific). They were tested for mycoplasma contamination and authenticated by STR profiling. Plasmid DNA was prepared using the Mini‐Prep procedure (Qiagen). The plasmid used were: human FLAG‐NLRP1, human FLAG‐NLRP3 (Tapia‐Abellan *et al*, 2021), human FLAG‐LRRFIP1 (#DU43760) and human FLAG‐FLII (#DU24044) from MRC PPU Reagents and Services, and human ASC‐eGFP (NM_013258, GenScript). Cells were transfected with Lipofectamine (Thermofisher) and examined at 48 h post‐transfection with a fluorescence microscope (Leica Microsystems).

### Mass spectrometry

Pull down elutions were separated by SDS–PAGE and stained with Brilliant Blue G‐Colloidal Concentrate (Sigma‐Aldrich) following the manufacturer's instructions. Gel bands were excised from the whole gel lane, destained, and proteins were digested in‐gel with trypsin (sequencing grade, Promega) overnight. The resulting peptide mixtures were analyzed by liquid chromatography (LC)‐tandem mass spectrometry (MS/MS) using Orbitrap Fusion Lumos Tribrid Mass Spectrometer (Thermo Electron, Bremen, Germany) online with a nanoLC Ultimate 3000 chromatography system (Dionex, Sunnyvale, CA) through a LC EASY‐Spray C18 column from Dionex. All raw LC–MS files were processed with MaxQuant software (version 1.5.3.8, www.maxquant.org) and searched against species‐specific Uniprot protein sequence databases and common contaminants using the Andromeda peptide search engine, with a false discovery rate of 0.01 at both the peptide and protein level.

The lists of proteins identified by MS were analyzed as follows. First, contaminants and proteins identified by a single peptide were eliminated. Next, only those proteins present in the samples of interest and absent in the control samples were considered. For the volcano plot, the differential analysis dataset was iterated upon, and each row was used as data point on a scatter plot. From each row, the −log_10_(*P*‐value) and the log_2_(fold‐change) were used as y and x coordinates respectively. Labelling of the datapoint was done using a combination of UniProt accession id and gene name of the protein. To set the color legend of the plot, by default, the data are separated into multiple combination of categories, > fold change cut‐off, ≤ fold change cut‐off, > *P*‐value cut‐off and ≤ *P*‐value cut‐off. Each of these pair of combinations between fold change and *P*‐value cut‐off was assigned a different color. If the data point belongs to a selected protein or set of selected protein, they were given a different color than those that have been already presented in the legend. Using the colors and coordinates data for each data point, the volcano plot figure was then created using the plotly.js module.

For the violin plot, from the original dataset, for each protein, samples with the same condition were grouped together and used with plotly.js to create the violin plots with the data itself formed the y‐axis range and the condition name being the x‐axis. With each unique condition, the violin shape was given a unique color. Within each violin shape was a box plot that was created by calculating either the standard error or standard deviation of the data points within the violin shape. The violin plot was also set to also showed each individual data point that was used to calculate the violin shape. Each data point had a slight horizontal x‐axis jitter to make it easier to identify each point separately. The y coordinate for each data point was the intensity of the data point.

### Immunoblotting and immunoprecipitation

Cells were lysed in 50 mM Tris–HCl (pH 7.5), 150 mM NaCl, 1% (w/v) NP‐40 and fresh protease inhibitor cocktail (1/20, #P8340, Sigma‐Aldrich). Protein quantification was performed with the BCA kit using BSA as standard. Cell lysates (40 μg) in SDS sample buffer were subjected to electrophoresis on a polyacrylamide gel and transferred to nitrocellulose membranes. The membranes were incubated for 1 h with TTBS containing 5% (w/v) skimmed dried milk powder or 2% (w/v) BSA. The membranes were immunoblotted in the same buffer 16 h at 4°C with the different primary antibodies diluted 1/1000. The blots were then washed with TTBS and incubated for 1 h at room temperature with secondary HRP‐conjugated antibodies diluted 2,500‐fold in 5% (w/v) skimmed milk in TTBS. After repeated washes, the signal was detected with enhanced chemiluminescence reagent and ChemiDoc XRS Biorad.

PhosTag SDS–PAGE was carried as described previously (Robinson *et al*, 2022). Briefly, 30 μM Phos‐tag Acrylamide (Wako Chemicals, AAL‐107) and 60 μM MnCl_2_ were added to homemade 10% SDS–PAGE gel. PhosTag‐SDS‐Agarose‐PAGE gels were made to 3% polyacrylamide and 0.5% agarose with a final concentration of Phos‐tag Acrylamide and MnCl_2_ as mentioned above. Cells were directly harvested using Laemmli buffer, lysed with an ultrasonicator, and loaded into the Phos‐tag gel to run. Once the run was completed, the polyacrylamide gel was washed in transfer buffer with 10 mM EDTA twice, subsequently washed once without EDTA, blotted onto 0.45 μm PVDF membranes (Bio‐rad), blocked with 3% milk, and incubated with primary and corresponding secondary antibodies.

Pull down assays were performed as previously described (Tyrkalska *et al*, 2017). HEK293T cells transfected as described above were washed twice with PBS, solubilized in lysis buffer (50 mM Tris–HCl, 150 mM NaCl, 1% NP40 and protease inhibitor cocktail for 30 min under agitation at 4°C and centrifuged (13,000 *g*, 10 min, 4°C). The cell lysate (1 mg) was incubated for 2 h at 4°C under gentle agitation with 40 μl of slurry of ANTI‐FLAG® M2 (Sigma‐Aldrich). The immunoprecipitates were washed four times with lysis buffer containing 0.15 mM NaCl and then twice with PBS. Finally, the resin was boiled 5 min at 95°C in SDS sample buffer, and the bound proteins were resolved on 10 or 15% SDS–PAGE and transferred to nitrocellulose membranes (BioRad) for 60 min at 100 V.

The primary antibodies used were rabbit human GATA1 (#3535, Cell Signaling), human NLRP1 (#AF6788, R&D Systems and #67980, Biolegend), human LRRFIP1 (#A303‐079A, Bethyl Laboratories), human FLII (#PA5‐21735, ThermoFisher Scientific), human ZAKα (#A301‐993A, Bethyl Laboratories), human phosphoP38 (#MA5‐15177, ThermoFisher Scientific), human DPP9 (MAB5419, R&D Systems), ACTB‐HRP (#sc‐47778, Santa Cruz Biotechnology), human CASP1 (#sc‐56036 Santa Cruz Biotechnology) and ANTI‐FLAG_M2‐HRP. The secondary antibodies used were anti‐sheep (#31480, Thermofisher), anti‐rabbit (#A6154, Sigma‐Aldrich), anti‐mouse (#A4416, Sigma‐Aldrich).

### Immunofluorescence

K562 cells (50,000) were seeded in Poly‐L‐Lys Cellware 12 mm cover (Corning) in 100 μl and allowed to adhere to the cover for 10 min at room temperature, and then culture medium with hemin and appropriate treatments were added. After treatment, cells were washed with PBS, fixed with 4% paraformaldehyde in PBS for 10 min, incubated 20 min at room temperature with 20 mM glycine, permeabilized with 0.5% NP40 and blocked for 1 h with 2% BSA. Cells were then labeled with the corresponding primary antibody diluted 1/200, followed by Alexa 594‐conjugated secondary antibody or Alexa 488‐conjugated secondary antibody diluted 1/400 (ThermoFisher Scientific). Samples were mounted using a Dako mounting medium and examined with a Leica laser scanning confocal microscope AOBS and software (Leica Microsystems). Images were acquired in a 1.024 × 1.024 pixel format in sequential scan mode between frames to avoid cross‐talk. The objective used was HCX PL APO CS × 63 and the pinhole value was 1, corresponding to 114.73 μm.

### Proximity ligation assay (PLA)

For specific protein–protein interaction, PLA was performed as described in the manufacture's protocol. Briefly, dK562 cells seeded in poly‐L‐Lys coverslips were washed with PBS and fixed in 4% formaldehyde in PBS, permeabilized for 1 h with 1% saponin, and blocked for 1 h in Duolink block solution. The primary antibodies were incubated in Duolink antibody dilution buffer overnight at 4°C. Samples were washed twice with 5% BSA in PBS for 10 min. Secondary antibodies from the kit were incubated for 1 h at 37°C. The secondary antibodies were washed twice with 1× buffer A for 5 min and the ligation step was performed at 37°C for 30 min. Then, the cells were washed twice with 1× buffer A, and the amplification reaction was carried out for 100 min at 37°C. Finally, the cells were washed twice for 10 min with 1× buffer B, once with 0.01× buffer B, mounted and analyzed by confocal microscopy as described above.

### Microarray assay

Total RNA was extracted from 10^6^ cells with TRIzol reagent and RNA Mini Kit (Thermo Fisher Scientific) following the manufacturer's instruction. The amount and quality of the RNA checked by Bioanalyzer (Agilent Technologies). ss‐cDNA was synthesized from 100 ng of each sample using the GeneChip WT Plus Reagent kit (Affymetrix ThermoFisher Scientific, P/N 902280), according to the protocol supplied by manufacturer. The amount and quality of ss‐cDNA was checked by Nanodrop and Bioanalyzer. ss‐cDNA targets were cleaned up and after fragmentation and terminal labelling, 2.3 μg of fragmented and biotinylated ss‐cDNA were included in the hybridization mix, using the GeneChip® Hybridization, Wash and Stain kit (Affymetrix ThermoFisher Scientific, P/N 900720) according to recommendations of manufacturer. The resulting preparations were hybridized to Human Clariom S Arrays (Affymetrix ThermoFisher Scientific, P/N 902926) designed to provide extensive coverage of all known well‐annotated genes including more than 337,000 transcripts to define the level of expression of more than 20,800 genes of human transcriptome.

After scanning, microarrays data were processed using Affymetrix Expression Command Console (Affymetrix ThermoFisher Scientific). Data analyses were then performed with RMA (Robust Multiarray Average) allowing that raw intensity values were background corrected, log_2_ transformed and then quantile normalized to obtain an individual intensity value for each probeset. Partek Genomics Suite and Partek Pathways software (Partek Incorporated, St. Louis, USA) were used for the statistical analysis and an ANOVA test was applied with a restrictive threshold at *P*‐value ≤ 0.05. The molecular interaction, reaction, and relation networks that showed differentially expressed genes (DEGs) were then analyzed using KEGG Pathways Kyoto Encyclopedia of Genes and Genomes.

### RT–qPCR

Total RNA extracted as indicated above was treated with DNase I, amplification grade (1 U/μg RNA; ThermoFisher Scientific). SuperScript III RNase H^−^ Reverse Transcriptase (ThermoFisher Scientific) was used to synthesize first‐strand cDNA with oligo(dT)18 primer from 1 ug of total RNA at 50°C for 50 min. Real‐time PCR was performed with an ABI PRISM 7500 instrument (Applied Biosystems) using SYBR Green PCR Core Reagents (Applied Biosystems). The reaction mixtures were incubated for 10 min at 95°C, followed by 40 cycles of 15 s at 95°C, 1 min at 60°C, and finally 15 s at 95°C, 1 min 60°C and 15 s at 95°C. For each mRNA, gene expression was normalized to *ACTB* content in each sample by the Pfaffl method (Pfaffl, 2001). The primers used are shown in (Appendix Table S2). In all cases, each PCR was performed with triplicate samples and repeated with at least two independent samples.

### Statistical analysis

Data were shown as mean ± SEM and analyzed by analysis of variance (ANOVA) and a Tukey or Bonferroni multiple range test to determine differences among groups. Differences between two samples were analyzed by Student *t*‐test. At least three independent experiments were performed with zebrafish larvae and biochemical studies. All larvae from the different independent experiments were pooled for plotting and statistical analysis. The number of total larvae analyzed in each experiment is indicated in all figures. Three independent caspase‐1 activity assays were performed in all experiments using a pool of 30 larvae and one representative experiment is shown with several technical replicates. Colony assays with M‐PBMC were performed with two technical replicates per donor. One hundred cells were analyzed in ASC speck formation assays and PLA.

## Author contributions

**Lola Rodríguez‐Ruiz:** Formal analysis; investigation. **Juan M Lozano‐Gil:** Formal analysis; investigation. **Elena Naranjo‐Sánchez:** Formal analysis; investigation. **Elena Martínez‐Balsalobre:** Formal analysis; investigation. **Alicia Martínez‐López:** Formal analysis; investigation. **Christophe Lachaud:** Formal analysis; investigation. **Miguel Blanquer:** Resources. **Toan K Phung:** Formal analysis. **Diana García‐Moreno:** Formal analysis; supervision; funding acquisition; investigation. **María L Cayuela:** Formal analysis; supervision; funding acquisition; visualization. **Sylwia D Tyrkalska:** Conceptualization; formal analysis; supervision; investigation. **Ana B Pérez‐Oliva:** Conceptualization; formal analysis; supervision; funding acquisition; investigation. **Victoriano Mulero:** Conceptualization; formal analysis; supervision; funding acquisition; visualization; writing – original draft; project administration; writing – review and editing.

## Disclosure and competing interests statement

A patent for the use of ZAKα inhibitors to treat anemia has been registered by Universidad de Murcia, IMIB Pascual Parrilla, and CIBERER (#P201831288). The authors declare no competing interest.

## For more information

- OMIM for Diamond‐Blackfan anemia: https://www.omim.org/entry/105650.
- Diamond‐Blackfan anemia Foundation: https://dbafoundation.org.

## Supporting information



Appendix S1

Source Data for Figure 1

Source Data for Figure 2

Source Data for Figure 3

Source Data for Figure 4

Source Data for Figure 5

Source Data for Figure 6

Source Data for Figure 7

Source Data for Figure 8

## Data Availability

Complete microarray data and microscopic images were deposited in GEO under the accession number GSE232511 (https://www.ncbi.nlm.nih.gov/geo/query/acc.cgi?acc=GSE232511) and in BioImage Archive under accession number S‐BIAD823 (https://www.ebi.ac.uk/biostudies/bioimages/studies/S‐BIAD823?key=75da6410‐05c3‐4358‐9110‐dd250e8db016), respectively.
