# Supplementary material for: ZAKα/P38 kinase signaling pathway regulates hematopoiesis by activating the NLRP1 inflammasome
Source: EMBO Mol Med. 2023 Sep 7;15(10):e18142. doi: 10.15252/emmm.202318142 (PMC10565642; doi:10.15252/emmm.202318142)
Supplement: Supplementary file 1 — Appendix S1 [file EMMM-15-e18142-s005.pdf]

Appendix data

ZAKα/P38 kinase signaling pathway regulates hematopoiesis by activating the NLRP1 inflammasome

The appendix file includes 2 Tables and 18 Figures

Contents

Appendix Table S1. Primers used in this study ………………………………………….…...……2

Appendix Table S2. gRNA used in this study …………………………………….……..….….2

Appendix Figure S1. Transcriptomic analysis of K562 cells after erythroid differentiation. ………………………………………………………………………………...…………..3

Appendix Figure S2. Single cell analysis of CASP1, GATA1 and NLRP1 in K562 cells after erythroid differentiation ……………………………………………….........................……4

Appendix Figure S3. Development of nlrp1 crispant larvae……………………………….....5

Appendix Figure S4. DPP9 does not regulate the NLRP1 inflammasome in K562 cells................7

Appendix Figure S5. Interactome of NLRP1 in K562 cells……………………………….….8

Appendix Figure S6. Expression of LRRFIP1 and FLII increases in K562 cells after erythroid differentiation…………………………………………………….................................9

Appendix Figure S7. Domain organization of human NLRP1……………………………..…...10

Appendix Figure S8. LRRFIP1 and FLII failed to inhibit the self-oligomerization of ASC speck in the absence of NLRP1……………………………………………………………………..…11

Appendix Figure S9. LRRFIP1 and FLII failed to inhibit the NLRP3 inflammasome………………………………………………………………………......12

Appendix Figure S10. Development of lrrfip1 crispant larvae…………………………………...13

Appendix Figure S11. Lrrfip1 regulates hematopoiesis through the Nlrp1 inflammasome in zebrafish……………………….………………………………………...…………15

Appendix Figure S12. Development of *flii* crispant and *flii*-overexpressing larvae…….……..16

Appendix Figure S13. Flii regulates hematopoiesis through the Nlrp1 inflammasome in zebrafish……………………………………………….……………………...…17

Appendix Figure S14. Flii-mediated regulation of hematopoiesis requires Lrrfip1 in wild type zebrafish.……………..........................……………………………………………...18

Appendix Figure S15. Flii-mediated regulation of hematopoiesis requires Lrrfip1 in Spint1a mutant zebrafish…………………………………………………………………………20

Appendix Figure S16. The ZAKα/P38 signaling pathways is activated in K562 after erythroid differentiation……………………...………………..…………………………….……..21

Appendix Figure S17. Comparison of human and zebrafish ZAKα and ZAKβ………………….22

Appendix Figure S18. Zaka mediates the activation of the Nlrp1 inflammasome to regulate zebrafish hematopoiesis………………………………………………………………….…24

Appendix Table S1. Primers used in this study. The gene symbols followed the zebrafish (https://zfin.atlassian.net/wiki/spaces/general/pages/1818394635/ZFIN+Zebrafish+Nomenclature+Conventions) and human (https://www.genenames.org/) nomenclature guidelines.

Species	Gene	Name	Sequence (5'→3')	Use
Danio rerio	*nlrp1*	F	TGAGCCTGACTGAGCTCTTGA	PCR
		R	AGCCAGTCCTGGTTACACTCT	
	flii	F	GGCCAGAGCAAACAACCTGAA	
		R	GCATATTGGGGAAAACCATGACTC	
	lrrfip1a	F	GCAGGACTGAGCACCATCTAC	
		R	TTCACCAATACATTACAACAAACCA	
	lrrfip1b	F	GTAGGCTGTGAAGTAAGTTGTACTAACTG	
		R	TTGGCACCATAGACATGCTCCTAG	
	zaka	F	TTGGCCATCATTTAATGGACCCGT	
		R	TTTTGGTTCAGTCGCCCAGCA	
	zakb	F	GTGTGGGATTCCTCTGCATCTTA	
		R	ATGCAGCTTTTGGGTGACGTA	
Homo sapiens	*NLRP1*	F	CACAGAAATCAGAGAAAGAGAG	RT-qPCR
		R	AAATCCTCATTTTTCCAGGG	
	LRRFIP1	F	GAGATGAAGGACTCTCTAGC	
		R	TGTTTTTCTCTTCGTACTGC	
	FLII	F	TATGTCACCAGGATGTATCG	
		R	CATACGTAGATGTCTAGCCC	
	ACTB	F	GGCACCACACCTTCTACAATG	
		R	GTGGTGGTGAAGCTGTAGCC	

Appendix Table S2. gRNA used in this study. The gene symbols followed the Zebrafish Nomenclature Guidelines (https://zfin.atlassian.net/wiki/spaces/general/pages/1818394635/ZFIN+Zebrafish+Nomenclature+Conventions).

Gene	Name	Sequence (5'→3')	Use
nlrp1	CD.Cas9.JTJN2987.AA	TCACAGAAGACTCAACTAGC	gRNA
lrrfip1a	CD.Cas9.JNBF0094.AA	GGAGAAGTACCGTAAGGCCA	
lrrfip1b	CD.Cas9.JMHW7812.AA	GGAGAAGTACCGTAAAGCCA	
flii	Dr.Cas9.FLII.1.AB	TGGAGTTCTCCAAGTCCCGG	
zaka	Dr.Cas9.ZAK.1.AB	AAGCCCCTCCAGACCTTTGA	
zakb	Dr.Cas9.LOC405768.1.AV	GGTCCCACAGGATAAAGAAG	

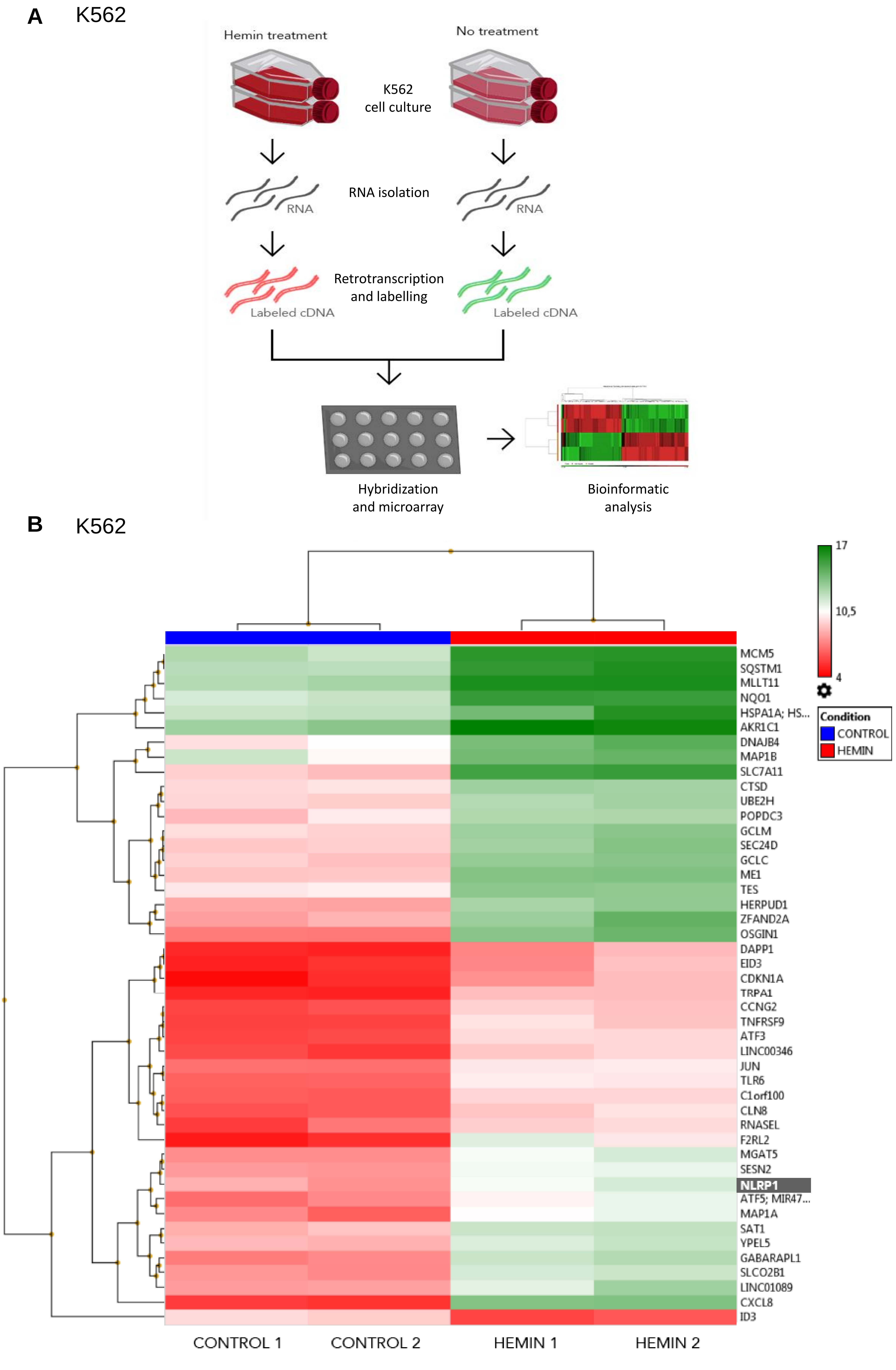

Appendix Figure S1 (related to Figure 1). Transcriptomic analysis of K562 cells after erythroid differentiation. (A) Experimental procedure. (B) Heat map of top upregulated genes in K562 cells differentiated with 50 µM hemin. Two biological replicates were used.

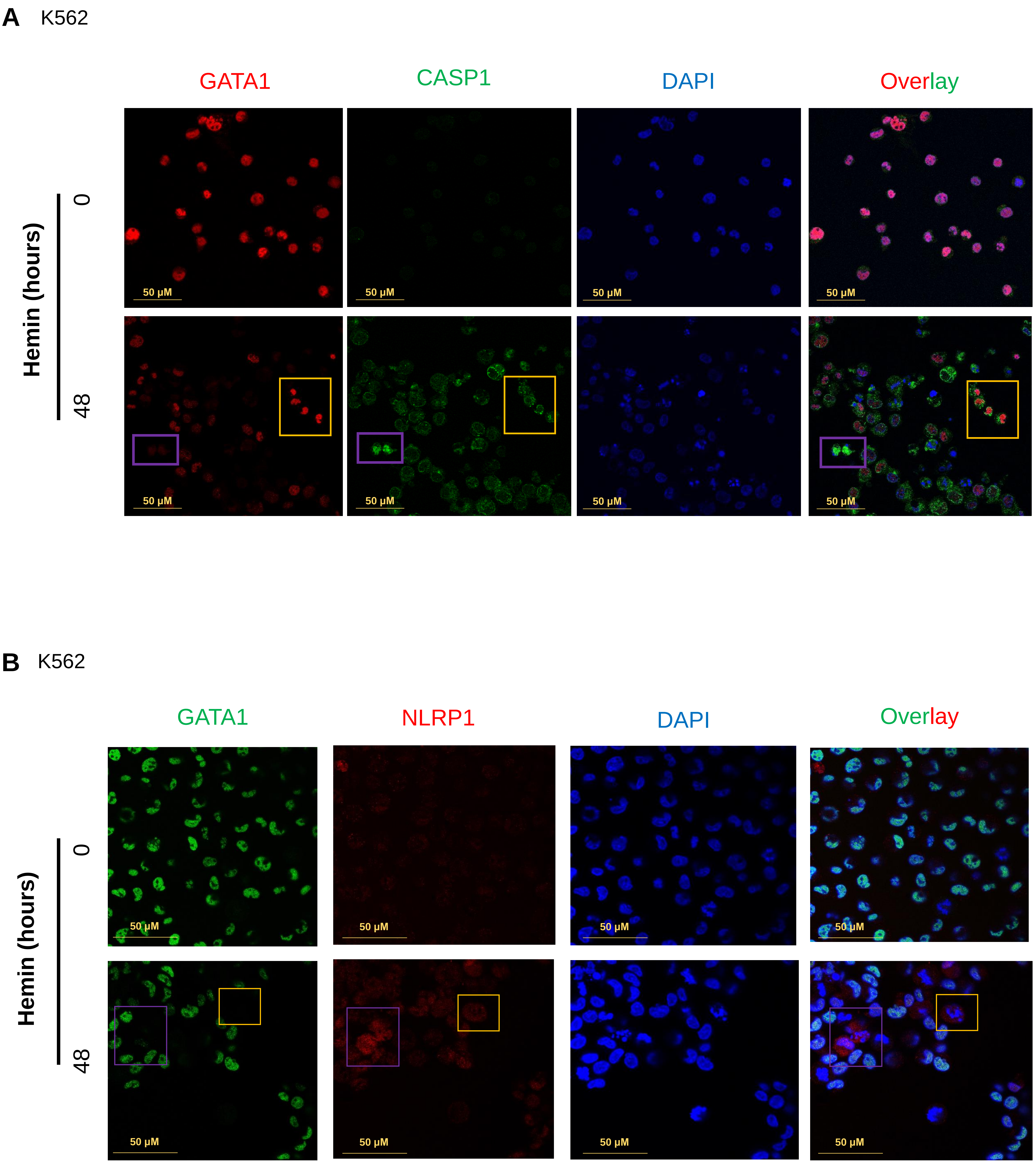

Appendix Figure S2 (related to Figure 1). Single cell analysis of CASP1, GATA1 and NLRP1 in K562 cells after erythroid differentiation. Immunofluorescence analysis of CASP1 and GATA1 (A) and GATA1 and NLRP1 (B) in K562 cells left untreated or treated with 50 μM hemin for 48h. Nuclei were counterstained with DAPI (blue). Squares highlight cells with disparate expression of the two proteins analyzed after erythroid differentiation.

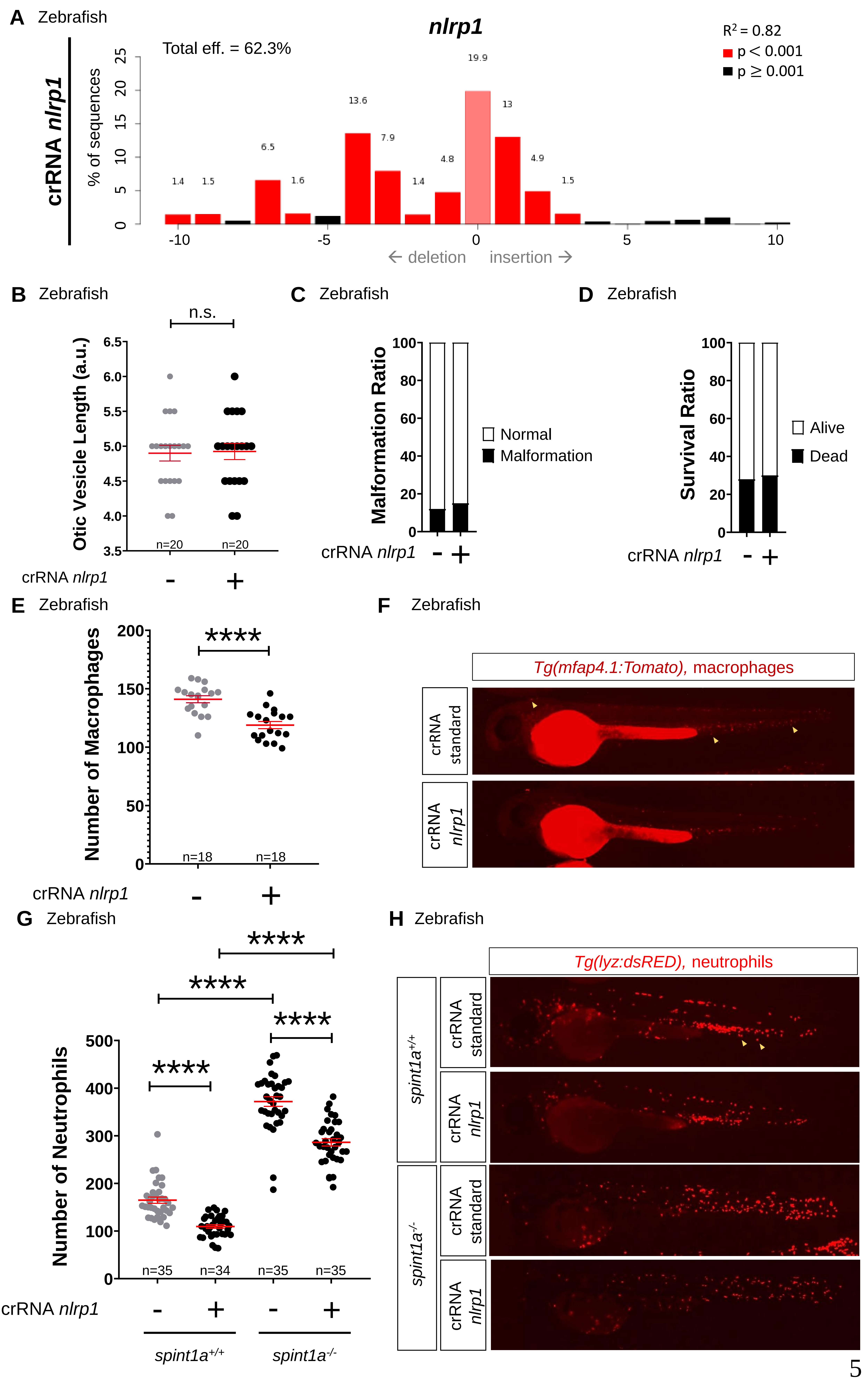
A Zebrafish
nlrp1
crRNA nlrp1
Total eff. = 62.3%
R2 = 0.82
p < 0.001
p ≥ 0.001
% of sequences
← deletion insertion →
B Zebrafish
n.s.
Otic Vesicle Length (a.u.)
n=20 n=20
crRNA nlrp1 - +
C Zebrafish
Malformation Ratio
Normal
Malformation
crRNA nlrp1 - +
D Zebrafish
Survival Ratio
Alive
Dead
crRNA nlrp1 - +
E Zebrafish
****
Number of Macrophages
n=18 n=18
crRNA nlrp1 - +
F Zebrafish
Tg(mfap4.1:Tomato), macrophages
crRNA standard
crRNA nlrp1
G Zebrafish
****
****
****
****
Number of Neutrophils
n=35 n=34 n=35 n=35
crRNA nlrp1 - + - +
spint1a+/+ spint1a-/-
H Zebrafish
Tg(lyz:dsRED), neutrophils
spint1a+/+
spint1a-/-
crRNA standard
crRNA nlrp1

Appendix Figure S3 (related to Figure 1). Development of *nlrp1* crispant larvae. (A) Analysis of genome editing efficiency in larvae injected with *nlrp1* crRNA/Cas 9 complexes and quantification rate of nonhomologous end joining mediated repair showing all insertions and deletions at the target site using TIDE (https://tide.nki.nl). (B-D) Developmental stage (B), malformation (C) and survival (D) of *nlrp1* crispant embryos were determined at 24 hpf. (E-G) Number of macrophages (E) and neutrophils (G) in wild type and Spint1a mutant larvae of 2 dpf obtained by injecting of one-cell stage embryos with standard and *nlrp1* crRNAs/Cas9 complexes. Representative images of macrophages (F) and neutrophils (H) (arrows) are also shown. Each dot represents one individual and the mean ± SEM for each group is also shown. P values were calculated using Student's t test (A-D) and one-way ANOVA and Tukey's multiple range test (F). n.s., non-significant; ****P<0.0001. a.u., arbitrary units.

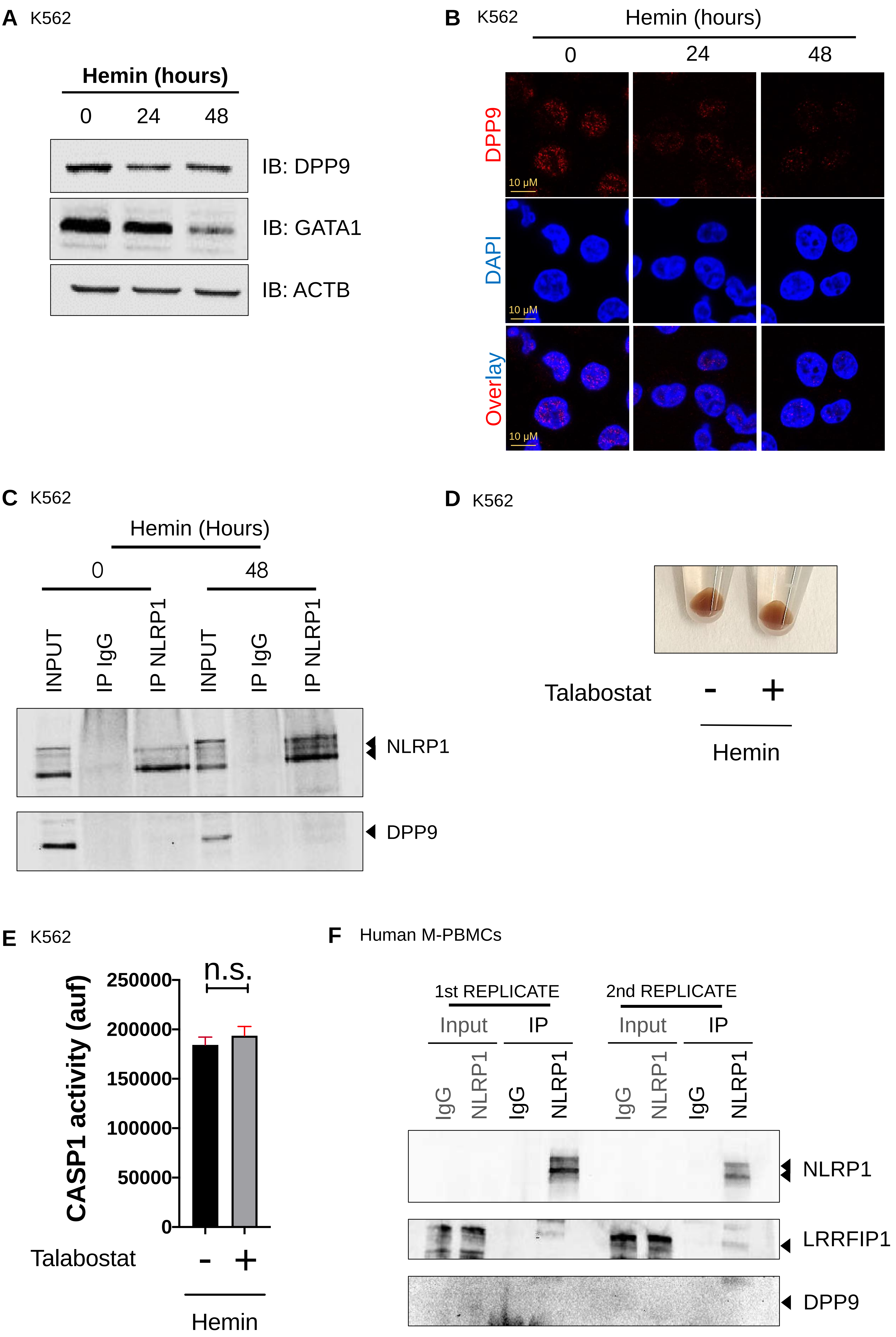

Appendix Figure S4 (related to Figure 2). DPP9 does not regulate the NLRP1 inflammasome in K562 cells. Western blot (A, C, F) and immunofluorescence (B) analysis of DPP9, GATA1, NLRP1 and LRRFIP1, hemoglobin accumulation (D), and caspase-1 activity (E) in K562 cells (A-E) and M-PBMCs (F). Nuclei were counterstained with DAPI in B.

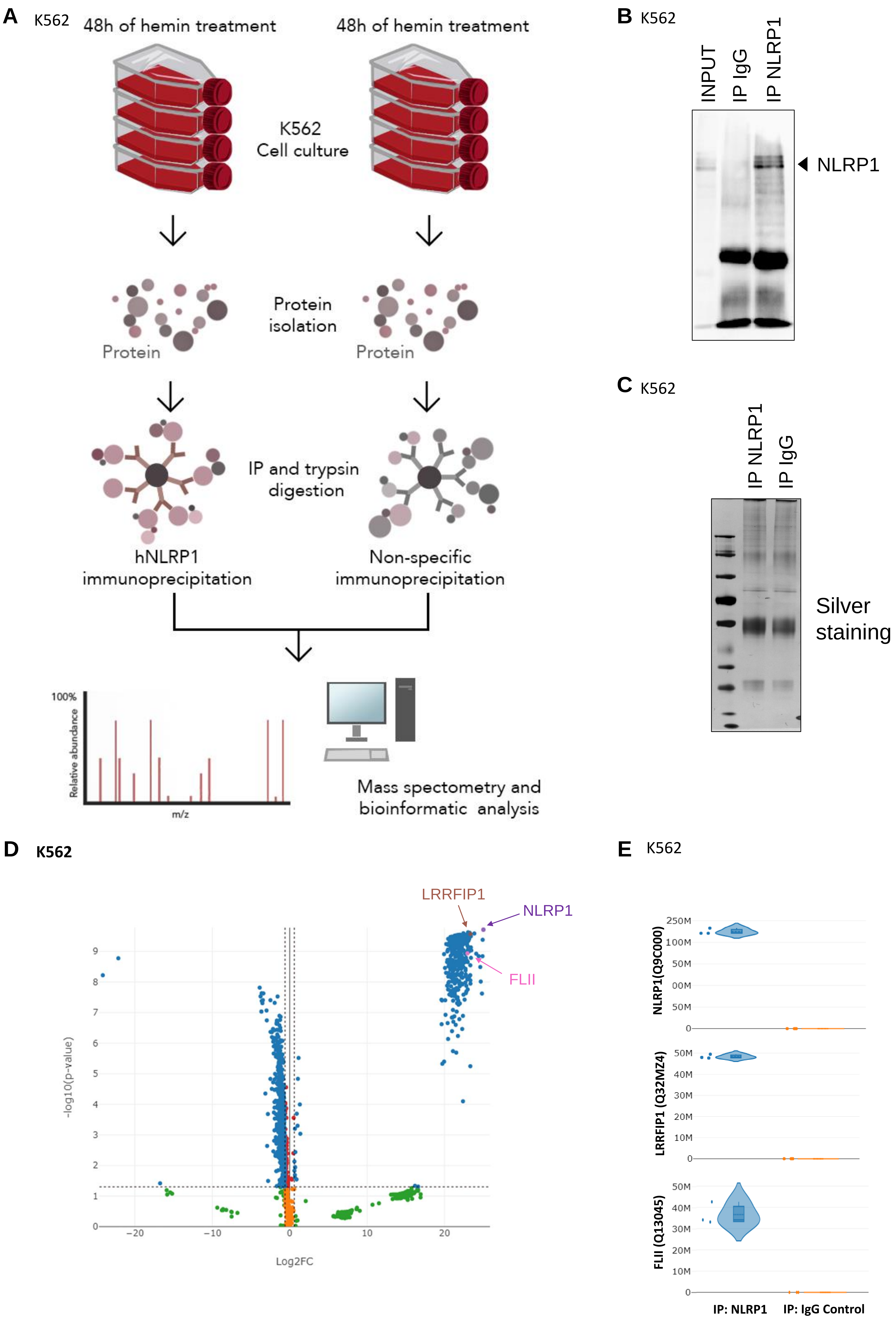

Appendix Figure S5 (related to Figure 2). Interactome of NLRP1 in K562 cells. (A) Experimental procedure used to identify the interactome of NLRP1. (B) Western blot analysis of immunoprecipitated NLRP1. (C) Immunoprecipitated proteins with NLRP1 stained with silver. (D) Volcano plot showing the NLRP1 interactors. Statistically significant interactors obtained in 3 biological replicates depicted in blue. (E) NLRP1, LRRFIP1 and FLII were immunoprecipitated in NLRP1 samples but not in control IgG.

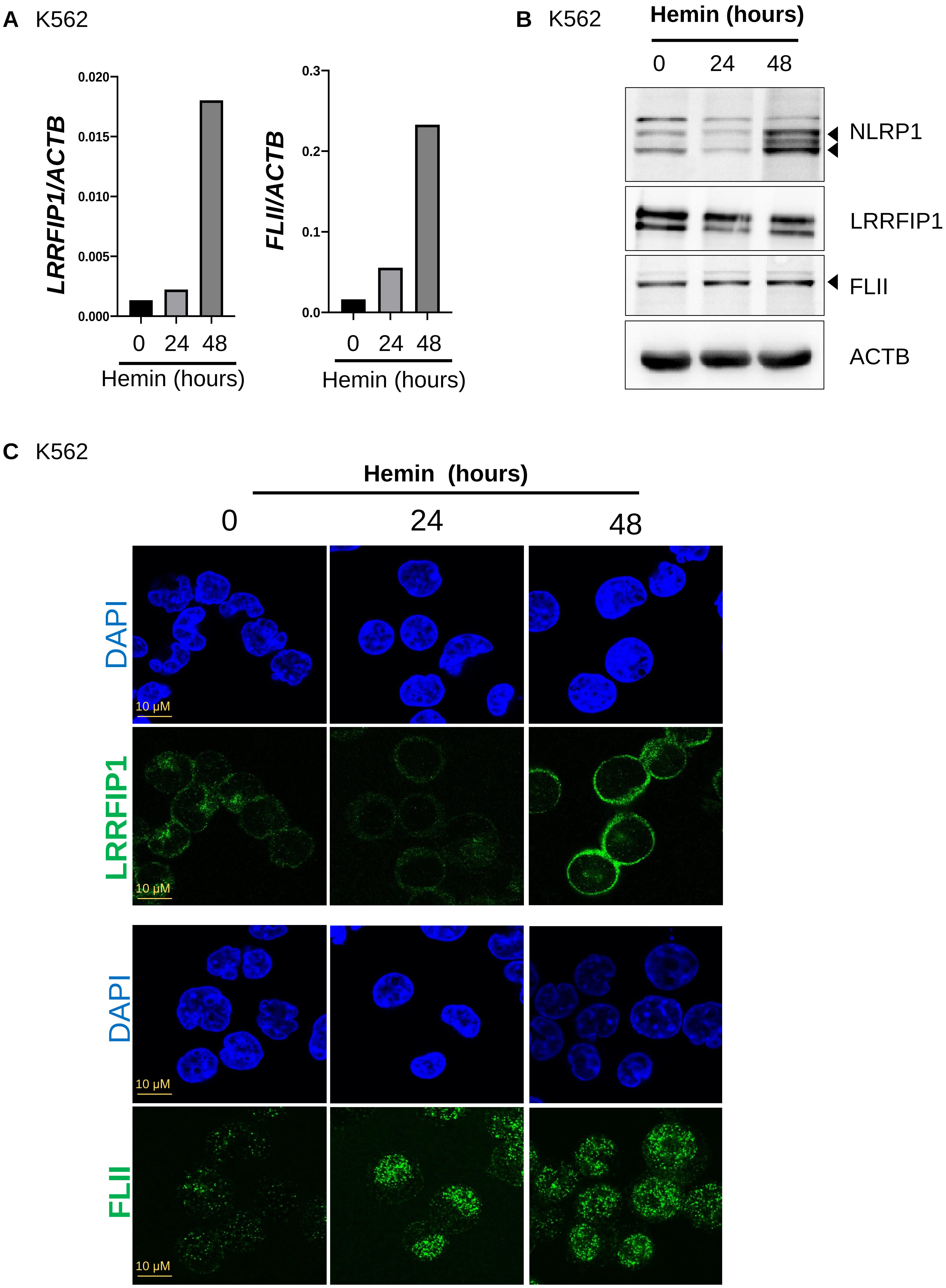

Appendix Figure S6 (related to Figure 2). Expression of LRRFIP1 and FLII increases in K562 cells after erythroid differentiation. LRRFIP1 and FLII expression analyzed by RT-qPCR (A), western blot (B) and immunofluorescence (C) in K562 cells differentiated with 50 µM hemin for 24 and 48 h. Nuclei were counterstained with DAPI in C.

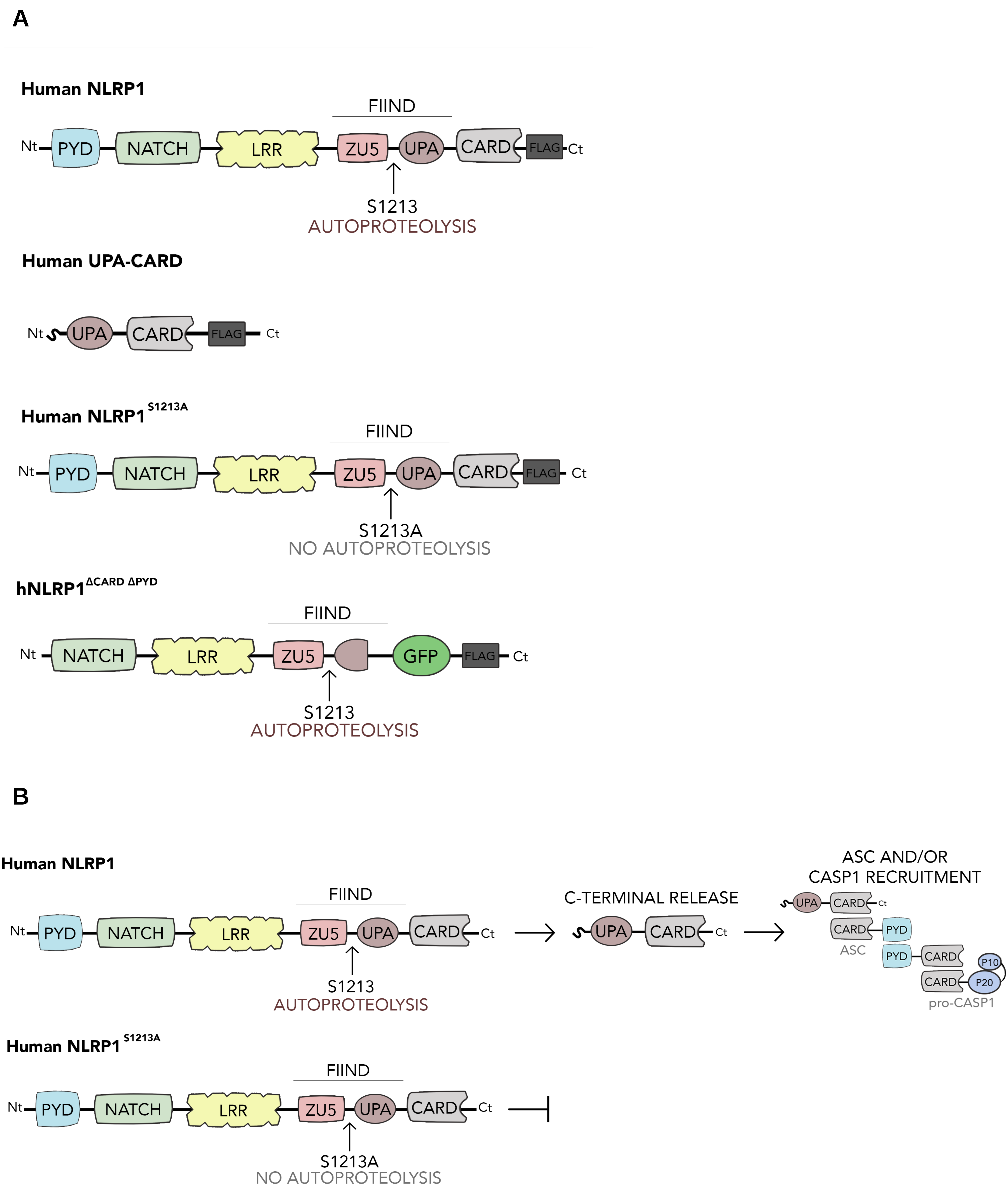

Appendix Figure S7 (related to Figure 2). Domain organization of human NLRP1. (A) Schemes showing domain organization of human wild type NLRP1, NLRP1-S1213A, UPA-CARD and NLRP1-ΔPYDΔCARD. Proposed mechanism of activation of NLRP1 and inability of the NLRP1-S1213A to activate by autoproteolysis.

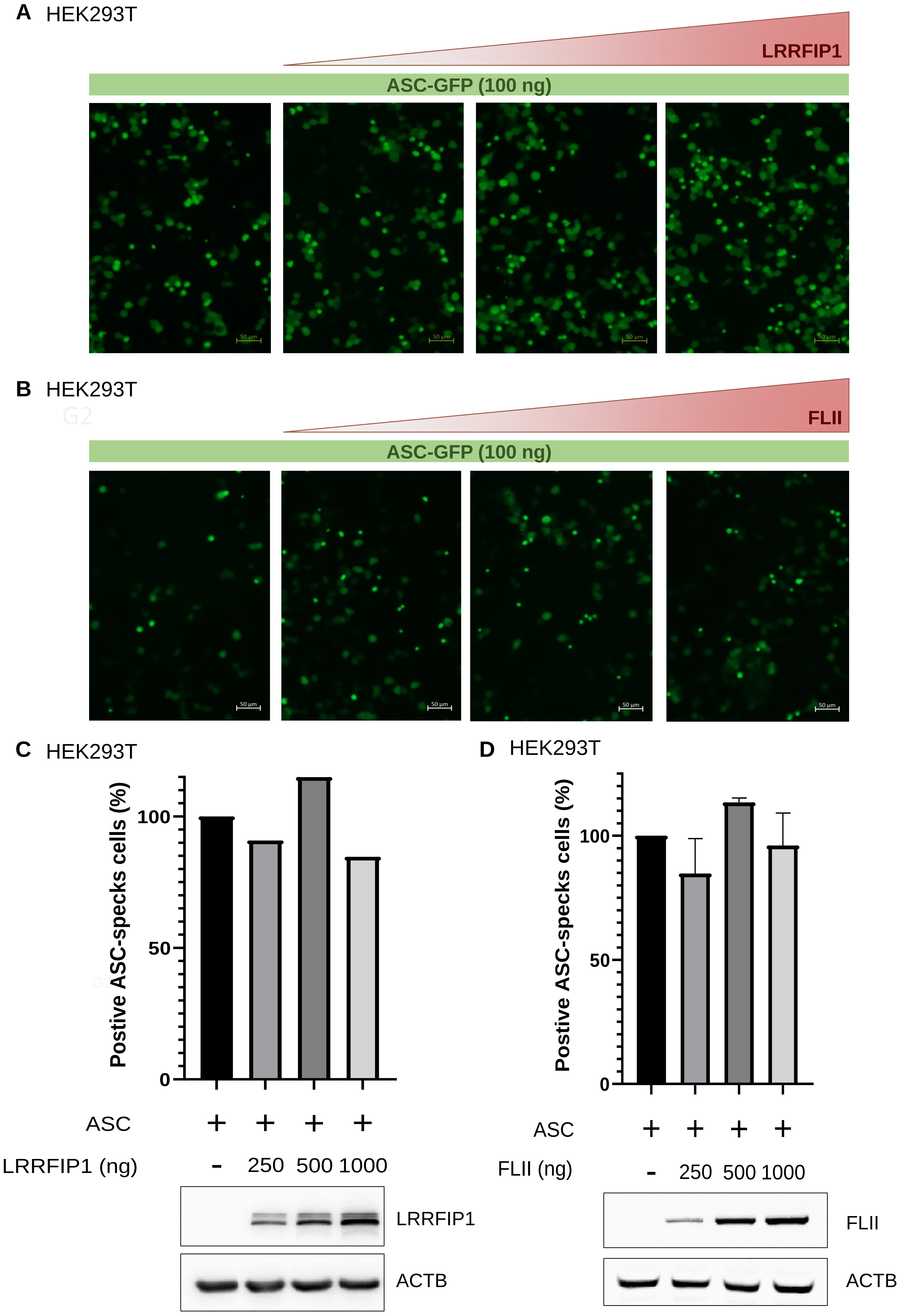

Appendix Figure S8 (related to Figure 3). LRRFIP1 and FLII failed to inhibit the self-oligomerization of ASC speck in the absence of NLRP1. HEK293T cells were transfected with 100 ng ASC-GFP and the indicated concentrations of FLAG-LRRFIP1 and FLAG-FLII plasmids, and the formation of ASC specks were analyzed by confocal microscopy at 24 h post-transfection. Representative images of ASC specks (A, B) and percentage of positive cells with ASC specks (C, D).

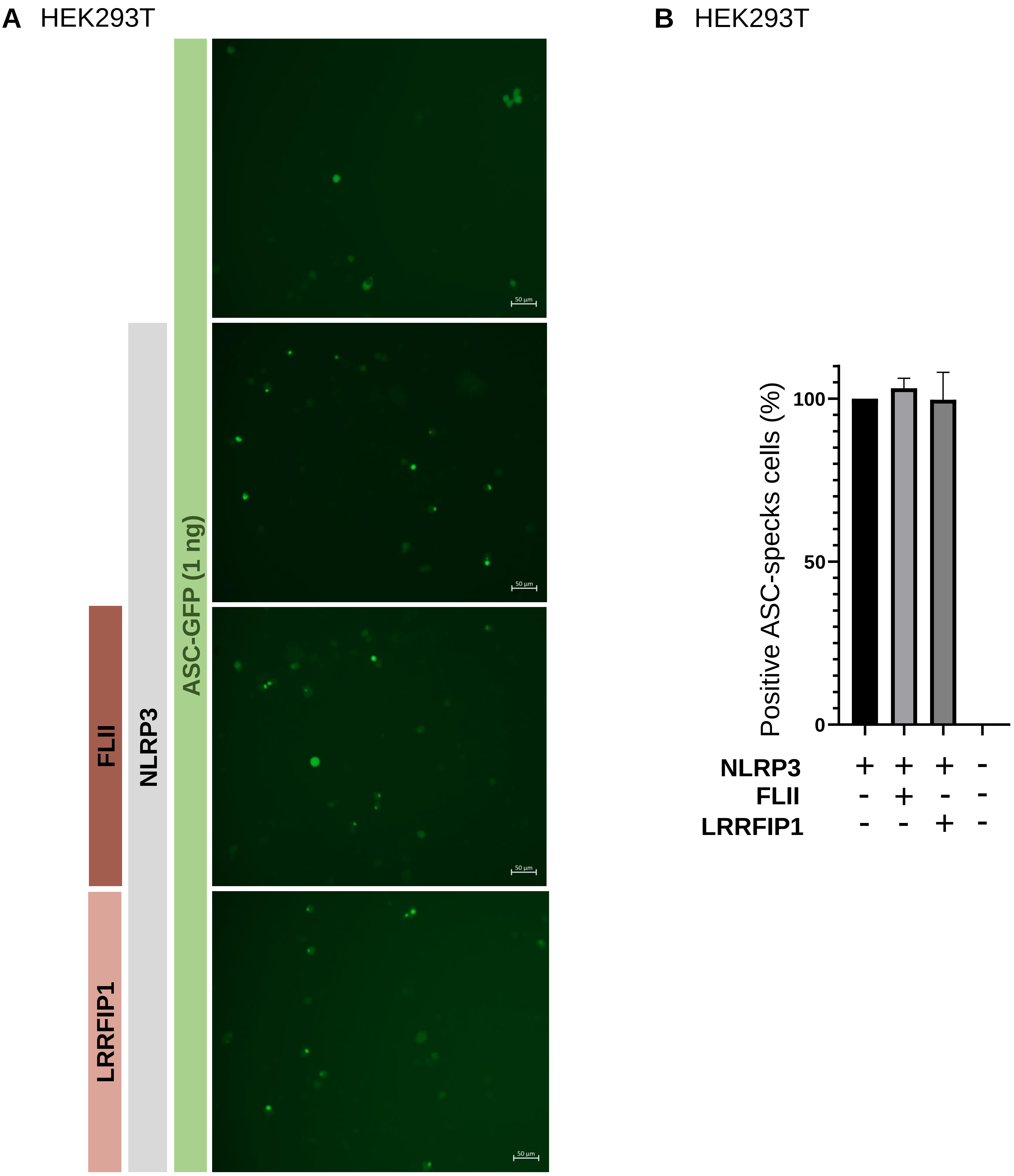

Appendix Figure S9 (related to Figure 3). LRRFIP1 and FLII failed to inhibit the NLRP3 inflammasome. HEK293T cells were transfected with 300 ng NLRP3, 1 ng ASC-GFP and 1000 ng of FLAG-LRRFIP1 and FLAG-FLII plasmids, and the formation of ASC specks were analyzed by confocal microscopy at 24 h post-transfection. Representative images of ASC specks (A) and percentage of positive cells with ASC specks (B).

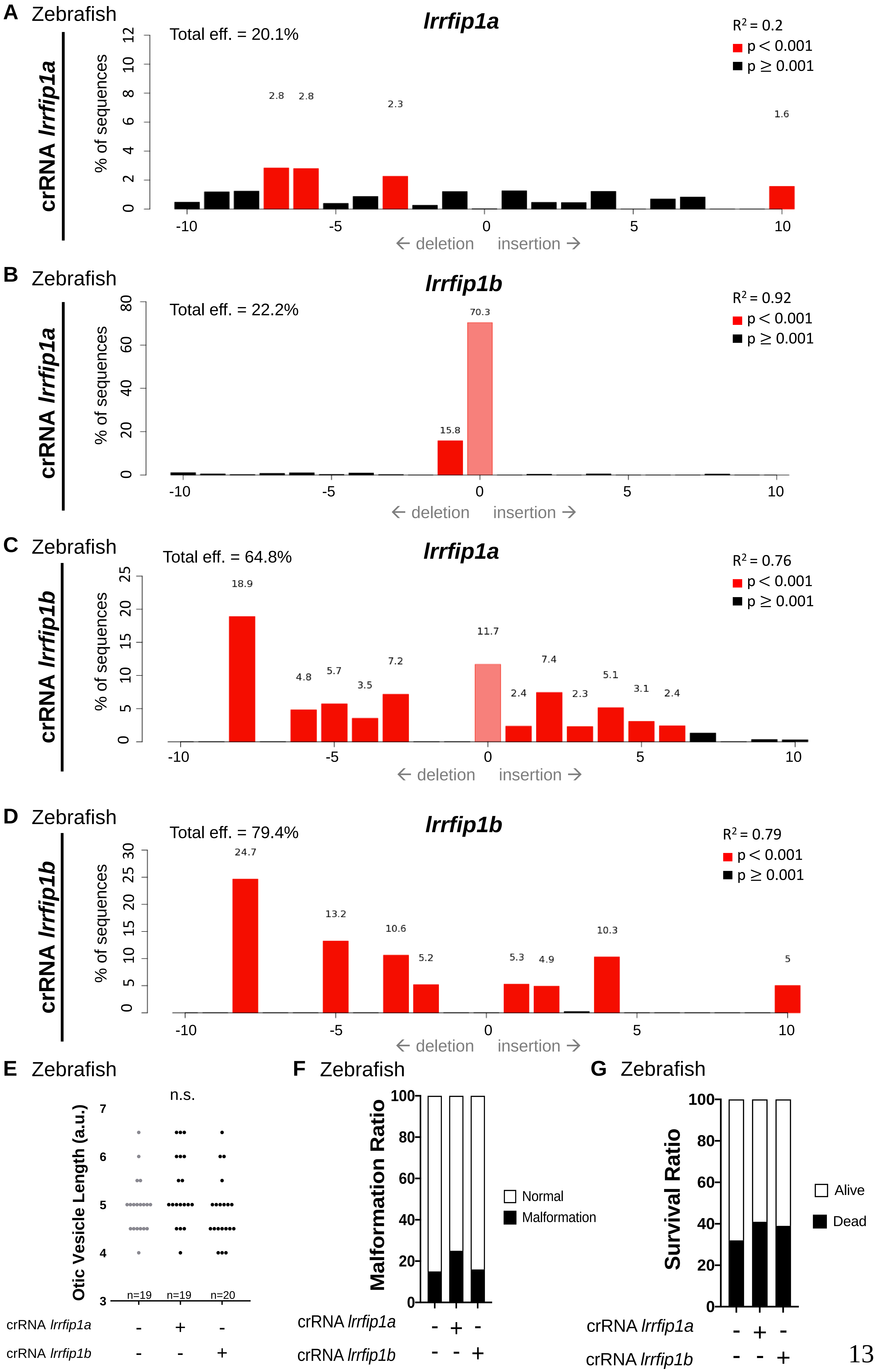
A Zebrafish
crRNA Irrfip1a
Irrfip1a
Total eff. = 20.1%
R² = 0.2
p < 0.001
p ≥ 0.001
% of sequences
2.8
2.8
2.3
1.6
← deletion insertion →
B Zebrafish
crRNA Irrfip1a
Irrfip1b
Total eff. = 22.2%
R² = 0.92
p < 0.001
p ≥ 0.001
% of sequences
70.3
15.8
← deletion insertion →
C Zebrafish
crRNA Irrfip1b
Irrfip1a
Total eff. = 64.8%
R² = 0.76
p < 0.001
p ≥ 0.001
% of sequences
18.9
4.8
5.7
3.5
7.2
11.7
2.4
7.4
2.3
5.1
3.1
2.4
← deletion insertion →
D Zebrafish
crRNA Irrfip1b
Irrfip1b
Total eff. = 79.4%
R² = 0.79
p < 0.001
p ≥ 0.001
% of sequences
24.7
13.2
10.6
5.2
5.3
4.9
10.3
5
← deletion insertion →
E Zebrafish
n.s.
Otic Vesicle Length (a.u.)
n=19
n=19
n=20
crRNA Irrfip1a - + -
crRNA Irrfip1b - - +
F Zebrafish
Malformation Ratio
Normal
Malformation
crRNA Irrfip1a - + -
crRNA Irrfip1b - - +
G Zebrafish
Survival Ratio
Alive
Dead
crRNA Irrfip1a - + -
crRNA Irrfip1b - - +

Appendix Figure S10 (related to Figure 4). Development of *lrrfip1* crispant larvae. (A-D) Analysis of genome editing efficiency in larvae injected with *lrrfip1a* (A, B) or *lrrfip1b* (C, D) crRNA/Cas 9 complexes and quantification rate of nonhomologous end joining mediated repair showing all insertions and deletions at the target site of *lrrfip1a* (A, C) and *lrrfip1b* (B, D) using TIDE (https://tide.nki.nl). Note that either *lrrfip1a* or *lrrfip1b* crRNAs/Cas9 complexes target both *lrrfip1* paralogs (see Appendix Figure S10). (E-G) Developmental stage (E), malformation (F) and survival (D) of *lrrfip1* crispant embryos were determined at 24 hpf. Each dot represents one individual and the mean ± SEM for each group is also shown. P values were calculated using one-way ANOVA and Tukey's multiple range test. n.s., non-significant. a.u., arbitrary units.

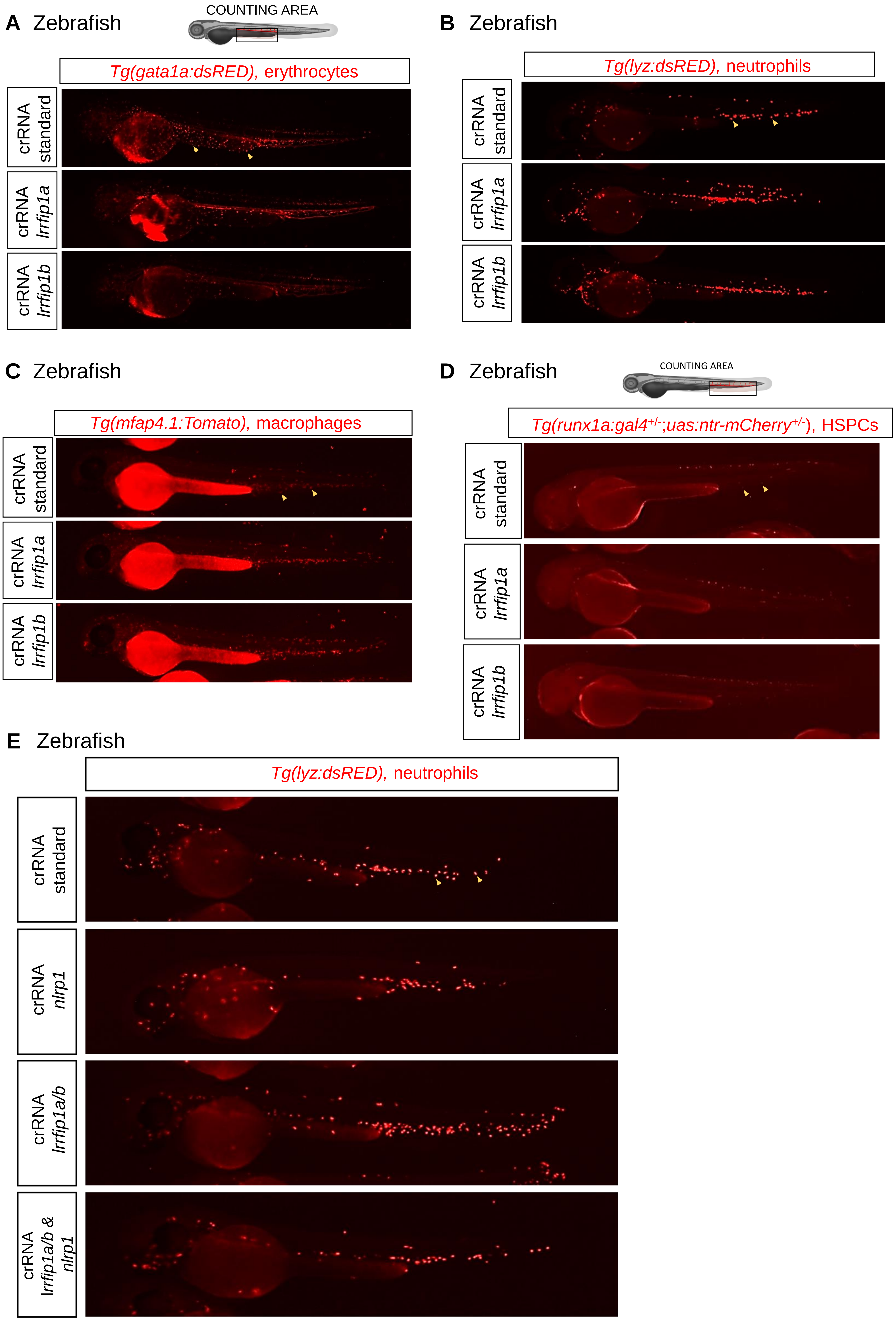

Appendix Figure S11 (related to Figure 4). Lrrfip1 regulates hematopoiesis through the Nlrp1 inflammasome in zebrafish. Representative images of erythrocytes (A), neutrophils (B, E), macrophages (C), and HSPCs (D) in *lrrfip1* crispant larvae of 2 dpf obtained by injecting one-cell stage embryos with standard, *nlrp1*, *lrrfip1a* and/or *lrrfip1b* crRNAs/Cas9 complexes. Fluorescent cells in each reporter line are indicated with arrowheads. Note that either *lrrfip1a* or *lrrfip1b* crRNAs/Cas9 complexes target both lrrfip1 paralogs (see Appendix Figure S10).

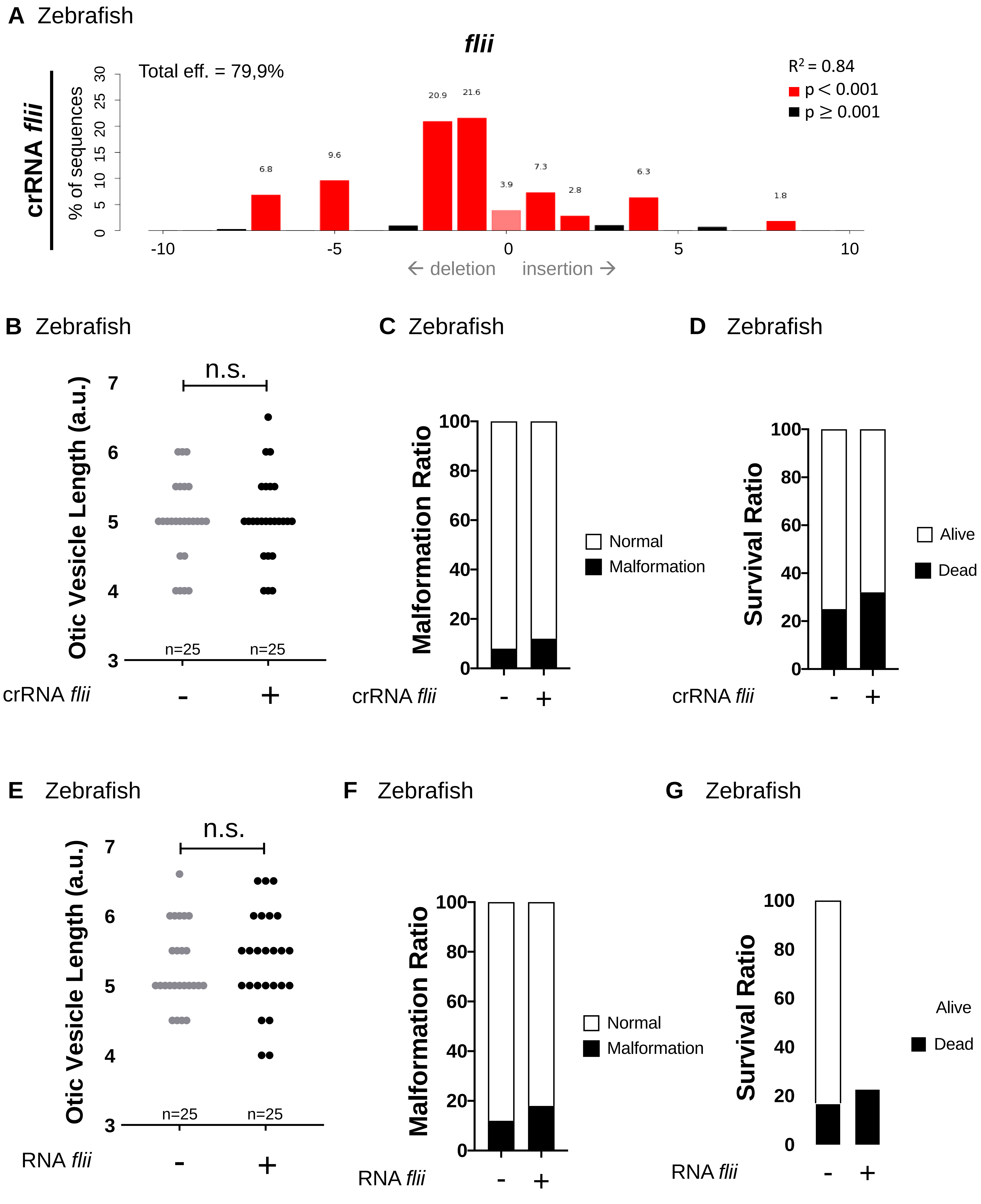

Appendix Figure S12 (related to Figure 5). Development of *flii* crispant and *flii*-overexpressing larvae. (A) Analysis of genome editing efficiency in larvae injected with *flii* crRNA/Cas 9 complexes and quantification rate of nonhomologous end joining mediated repair showing all insertions and deletions at the target site using TIDE (https://tide.nki.nl). (B-G) Developmental stage (B, E), malformation (C, F) and survival (D, G) of *flii* crispant (B-D) and forced to express *flii* mRNA (E-G) embryos were determined at 24 hpf. Each dot represents one individual and the mean ± SEM for each group is also shown. P values were calculated using Student's *t* test. n.s., non-significant. a.u., arbitrary units.

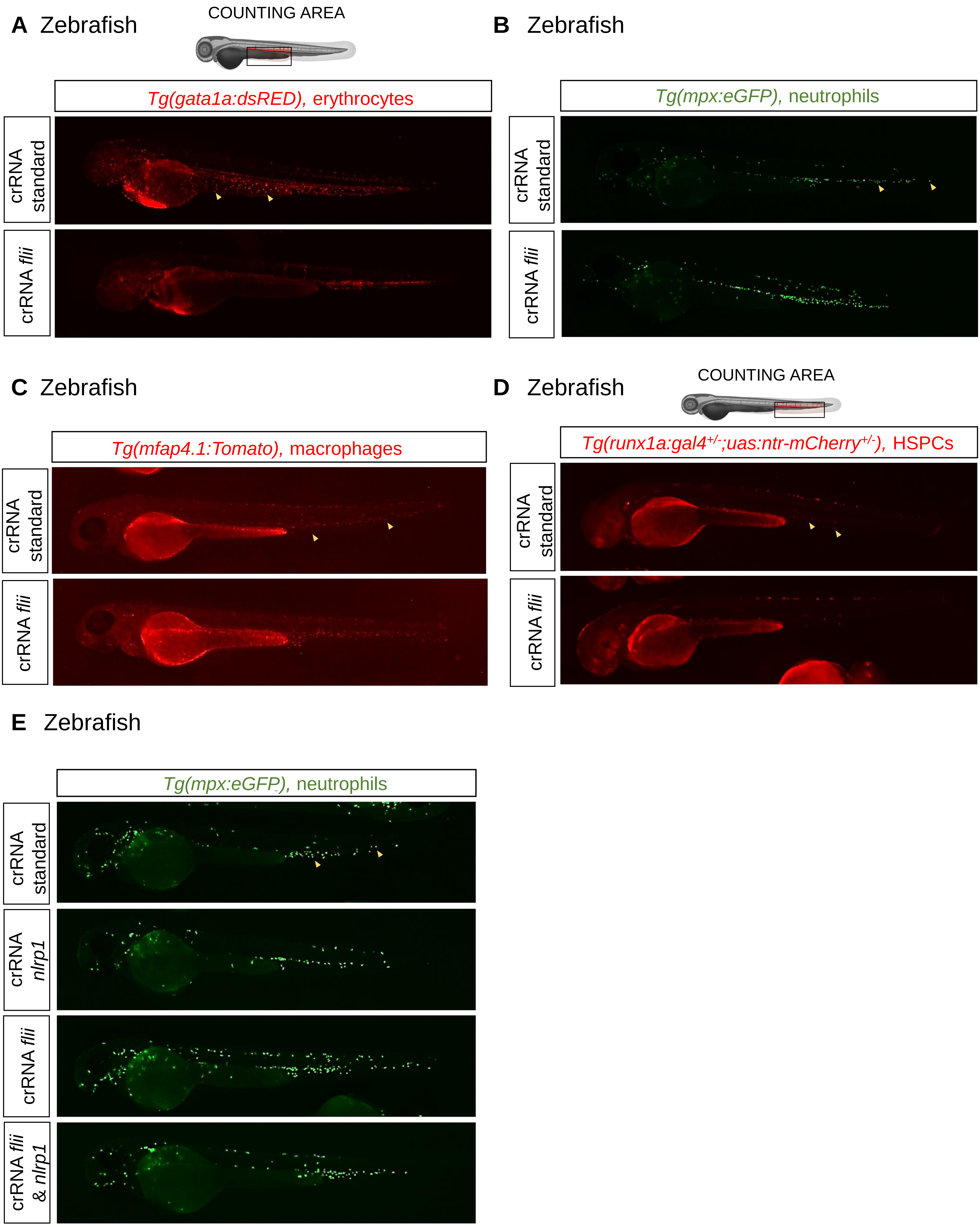

Appendix Figure S13 (related to Figure 5). Flii regulates hematopoiesis through the Nlrp1 inflammasome in zebrafish. Representative images of erythrocytes (A), neutrophils (B, E), macrophages (C), and HSPCs (D) in *flii* and/or *nlrp1* crispant larvae of 3 dpf obtained by injecting one-cell stage embryos with standard, *nlrp1* and/or *flii* crRNAs/Cas9 complexes. Fluorescent cells in each reporter line are indicated with arrowheads.

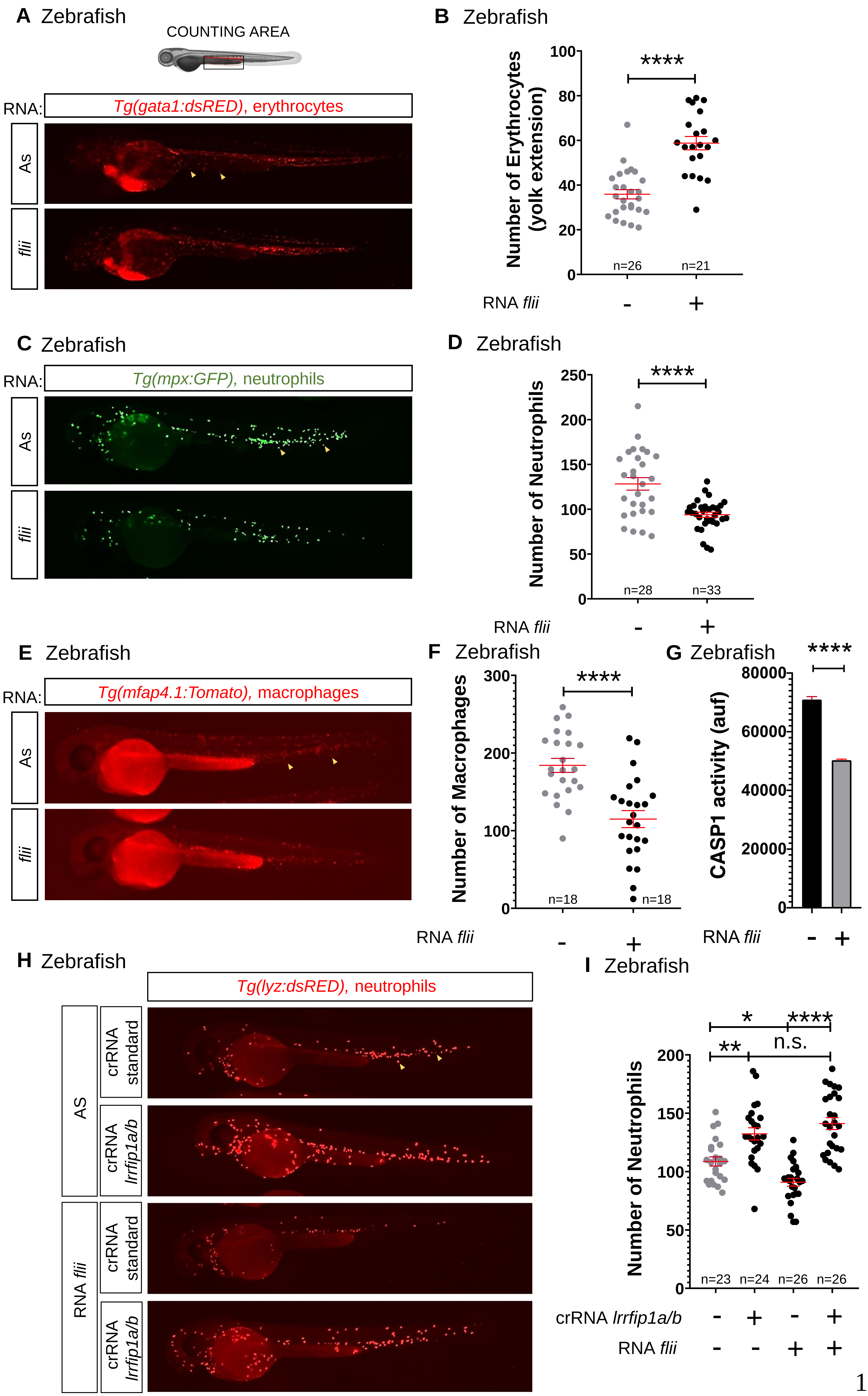
A Zebrafish
COUNTING AREA
RNA:
Tg(gata1:dsRED), erythrocytes
As
flii
B Zebrafish
Number of Erythrocytes (yolk extension)
****
n=26
n=21
RNA flii
-
+
C Zebrafish
RNA:
Tg(mpx:GFP), neutrophils
As
flii
D Zebrafish
Number of Neutrophils
****
n=28
n=33
RNA flii
E Zebrafish
RNA:
Tg(mfap4.1:Tomato), macrophages
As
flii
F Zebrafish
Number of Macrophages
****
n=18
n=18
G Zebrafish
CASP1 activity (auf)
****
H Zebrafish
Tg(lyz:dsRED), neutrophils
AS
RNA flii
crRNA standard
crRNA lrrfip1a/b
I Zebrafish
Number of Neutrophils
*
****
**
n.s.
n=23
n=24
n=26
n=26
crRNA lrrfip1a/b
RNA flii

Appendix Figure S14 (related to Figure 5). Flii-mediated regulation of hematopoiesis requires Lrrfip1 in wild type zebrafish. Representative images (A, C, E, H) and number (B, D, F, I) of erythrocytes (A, B), neutrophils (C, D, H, I) and macrophages (E, F), and caspase-1 activity (G) in 2 dpf larvae forced to express Flii obtained by injecting one-cell stage embryos with *flii* mRNA and/or standard or *lrrflip1a/b* crRNAs/Cas9 complexes. Fluorescent cells in each reporter line are indicated with arrowheads. Each dot represents one individual and the mean ± SEM for each group is also shown. P values were calculated by Student's *t* test (A-C) or one-way ANOVA and Tukey's multiple range test (E). n.s., non-significant. $*P<0.05$; $**P<0.01$; $****P<0.0001$.

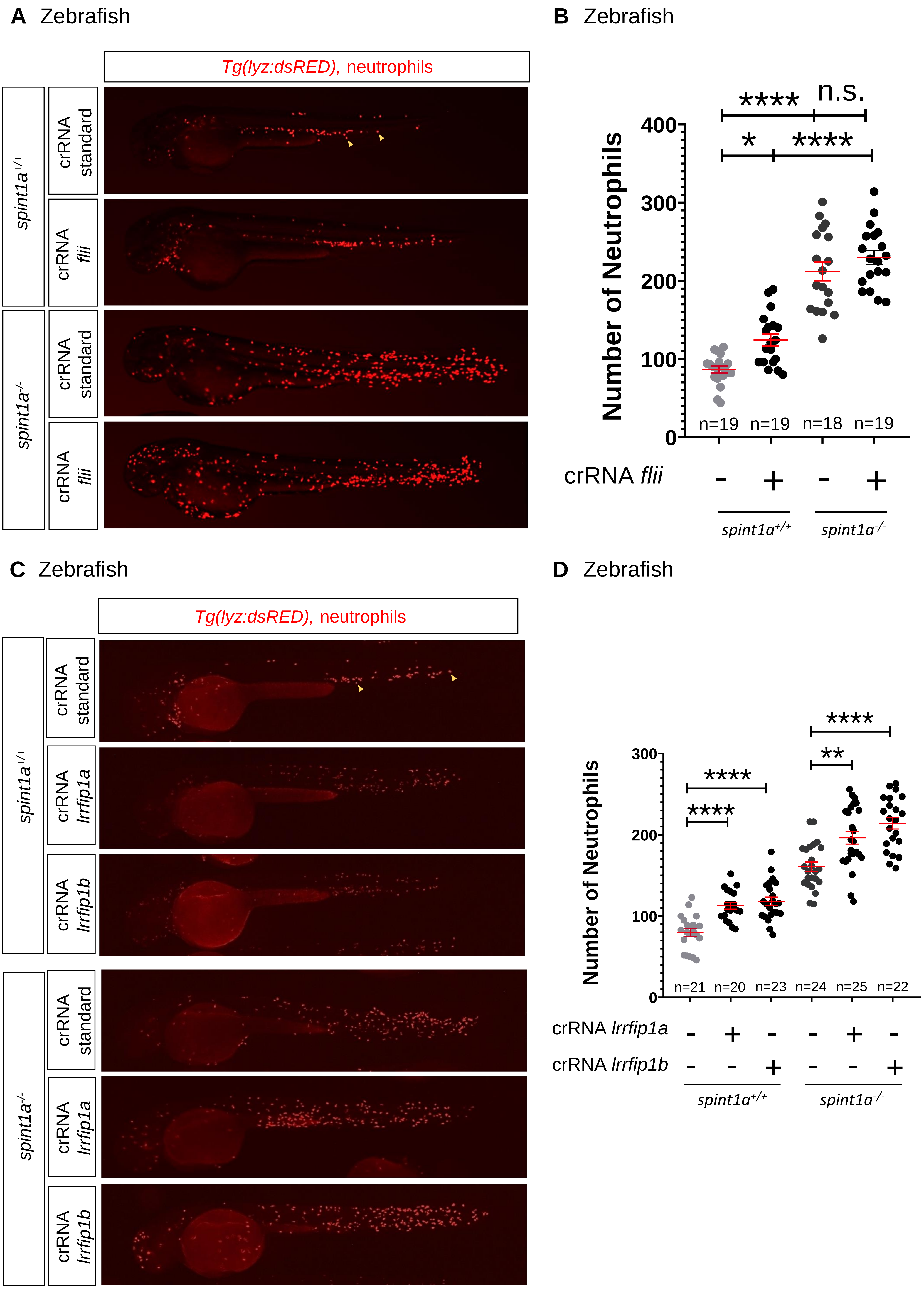

Appendix Figure S15 (related to Figures 4 and 5). Flii-mediated regulation of hematopoiesis requires Lrrfip1 in Spint1a mutant zebrafish. Representative images and number of neutrophils in *flii* (A, B) and *lrrfip1* crispant (C, D) larvae of 2 dpf obtained by injecting one-cell stage wild type and Spinta1 mutant embryos with standard, *flii* or *lrrflip1a/b* crRNAs/Cas9 complexes. Fluorescent cells in each reporter line are indicated with arrowheads. Each dot represents one individual and the mean ± SEM for each group is also shown. P values were calculated by one-way ANOVA and Tukey's multiple range test. n.s., non-significant. *P<0.05; **P<0.01; ****P<0.0001.

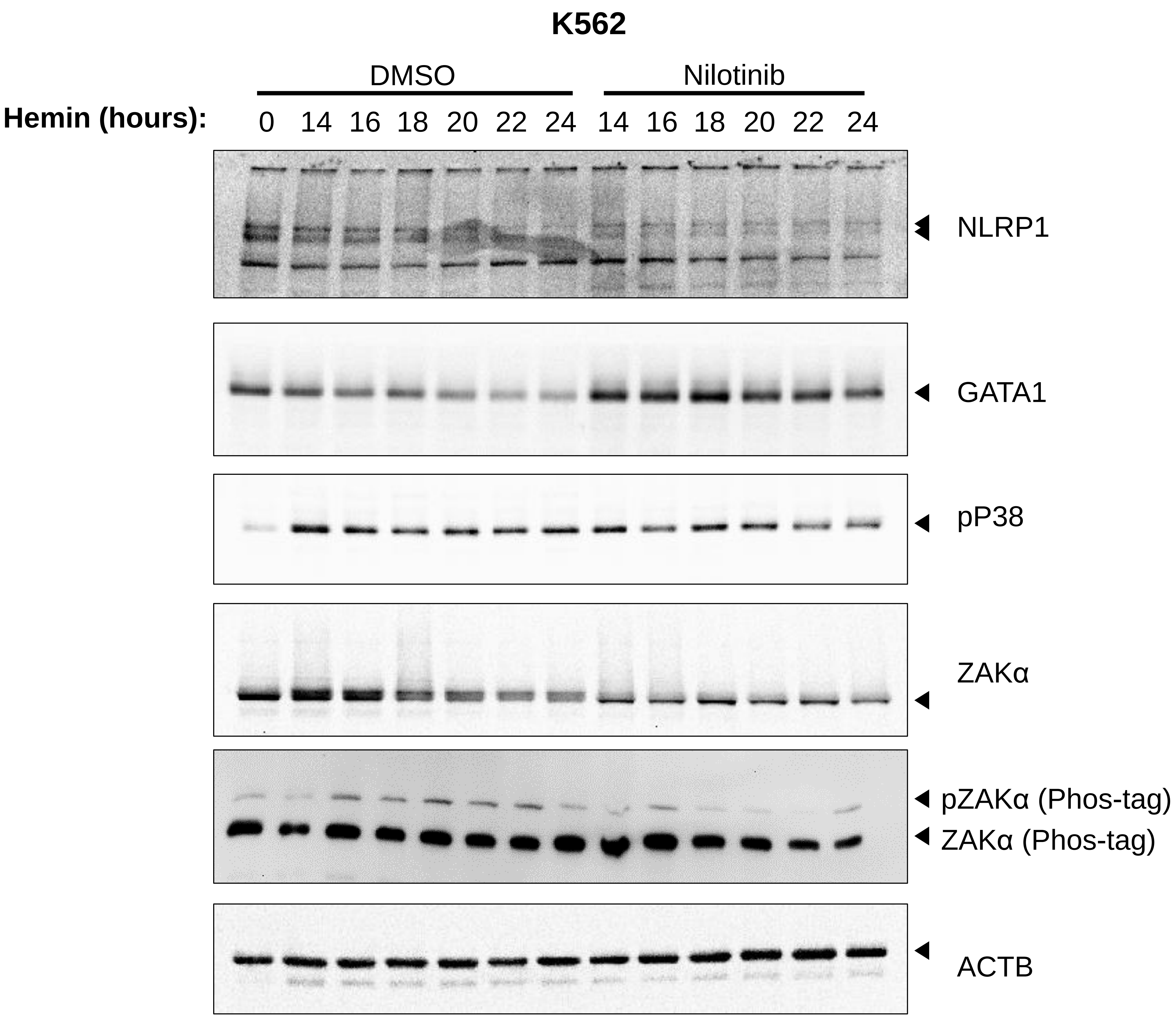

Appendix Figure S16 (related to Figure 6). The ZAKα/P38 signaling pathways is activated in K562 after erythroid differentiation. K562 cells were pretreated with 0.1 μM nilotinib for 24 h and then differentiated with 50 μM hemin for the indicated times. The amounts of NLRP1, GATA, phosphorylated P38, total ZAKα , phosphorylated ZAKα (Phos-tag) and ACTB were then evaluated by western blot.

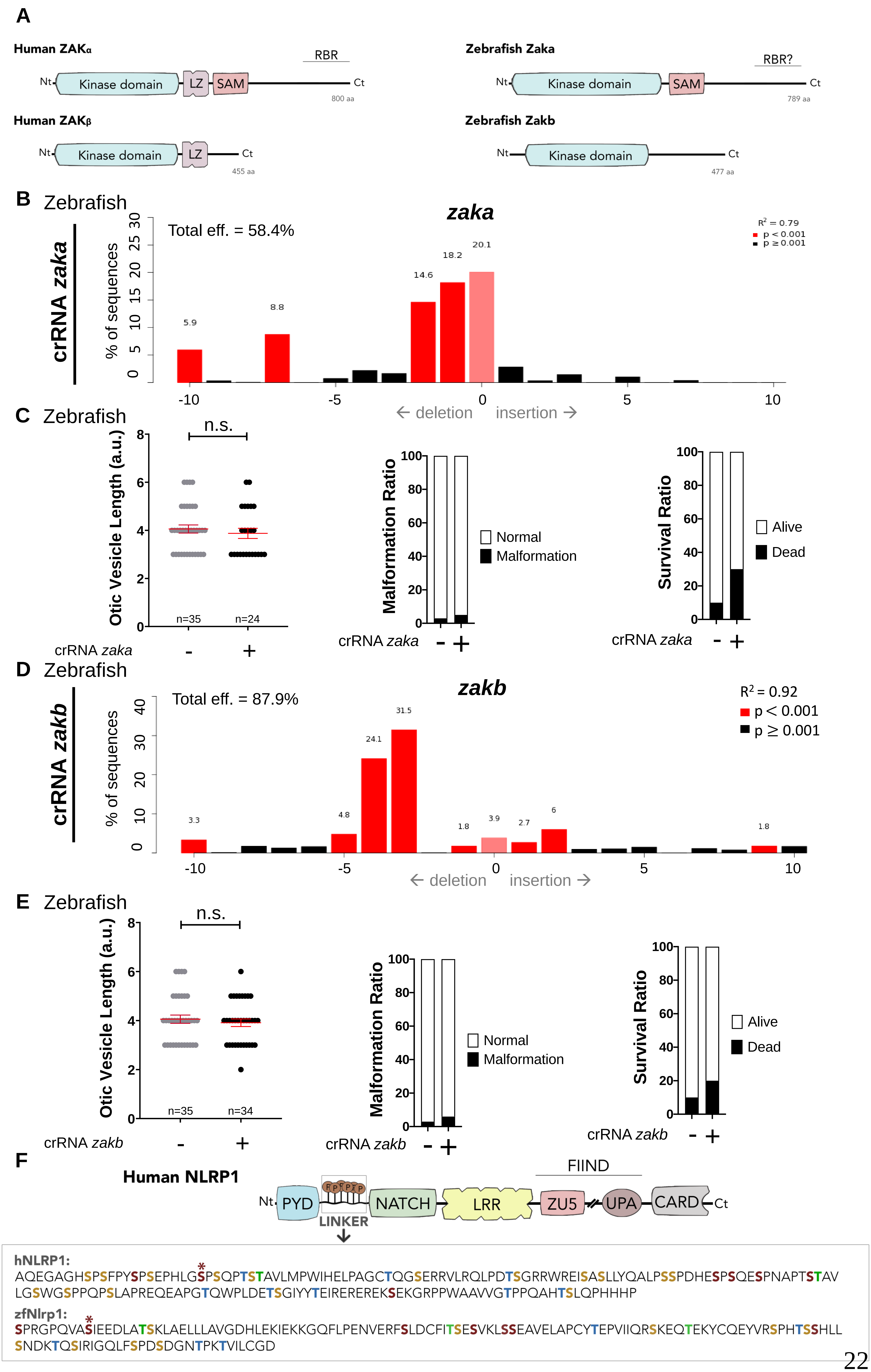
A
Human ZAKα
RBR
Nt
Kinase domain
LZ
SAM
Ct
800 aa
Human ZAKβ
Nt
Kinase domain
LZ
Ct
455 aa
Zebrafish Zaka
RBR?
Nt
Kinase domain
SAM
Ct
789 aa
Zebrafish Zakb
Nt
Kinase domain
Ct
477 aa
B Zebrafish
crRNA zaka
zaka
Total eff. = 58.4%
% of sequences
R² = 0.79
p < 0.001
p ≥ 0.001
5.9
8.8
14.6
18.2
20.1
-10
-5
0
5
10
← deletion
insertion →
C Zebrafish
Otic Vesicle Length (a.u.)
n.s.
n=35
n=24
crRNA zaka
-
+
Malformation Ratio
Normal
Malformation
Survival Ratio
Alive
Dead
D Zebrafish
crRNA zakb
zakb
Total eff. = 87.9%
% of sequences
R² = 0.92
p < 0.001
p ≥ 0.001
3.3
4.8
24.1
31.5
1.8
3.9
2.7
6
1.8
← deletion
insertion →
E Zebrafish
Otic Vesicle Length (a.u.)
n.s.
n=35
n=34
crRNA zakb
Malformation Ratio
Normal
Malformation
Survival Ratio
Alive
Dead
F
Human NLRP1
FIIND
Nt
PYD
NATCH
LRR
ZU5
UPA
CARD
Ct
LINKER
hNLRP1:
AQEGAGHSPSFPYSPSEPHLGSPSQPTSTAVLMPWIHELPAGCTQGSERRVLRQLPDTSGRRWREISASLLYQALPSSPDHESPSQESPNAPTSTAV
LGSWGSPPQPSLAPREQEAPGTQWPLDETSGIYYTEIREREREKSEKGRPPWAAVVGTPPQAHTSLQPHHHP
zfNlrp1:
SPRGPQVASIEEDLATSKLAELLLAVGDHLEKIEKKGQFLPENVERFSLDCFITSESVKLSSEAVELAPCYTEPVIIQRSKEQTEKYCQEYVRSPHTSSHLL
SNDKTQSIRIGQLFSPDSDGNTPKTVILCGD

Appendix Figure S17 (related to Figure 7). Comparison of human and zebrafish ZAKα and ZAKβ. (A) Schemes showing domain organization of human and zebrafish ZAKα and ZAKβ. (B, D) Analysis of genome editing efficiency in larvae injected with either *zaka* (B) or *zakb* (D) crRNA/Cas 9 complexes and quantification rate of nonhomologous end joining mediated repair showing all insertions and deletions at the target site using TIDE (https://tide.nki.nl). (C, E) Developmental stage, malformation, and survival of *zaka* (C) and *zakb* crispant (E) embryos were determined at 24 hpf. (F) Linker domains of human and zebrafish NLRP1 showing the conservation of serine and threonine residues. All serine residues are shown in yellow, those conserved in both proteins are highlighted in red, and S107 are marked with asterisks. Threonine residues are shown in blue and those conserved between both proteins are highlighted in green. Each dot represents one individual and the mean ± SEM for each group is also shown. P values were calculated by Student's *t* test. n.s., non-significant. a.u., arbitrary units.

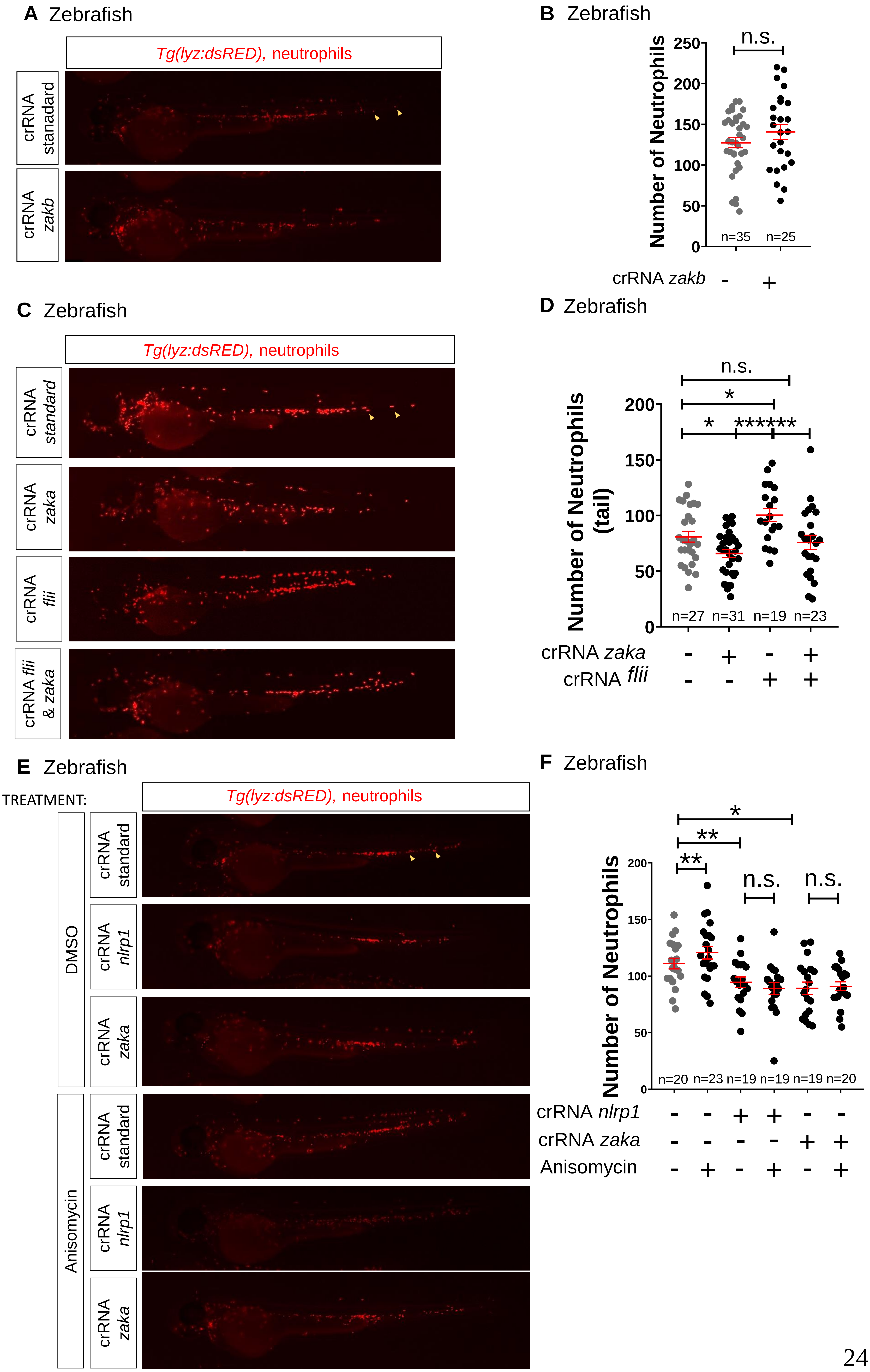

A Zebrafish
Tg(lyz:dsRED), neutrophils
crRNA stanadard
crRNA zakb
B Zebrafish
Number of Neutrophils
n.s.
n=35 n=25
crRNA zakb - +
C Zebrafish
Tg(lyz:dsRED), neutrophils
crRNA standard
crRNA zaka
crRNA flii
crRNA flii & zaka
D Zebrafish
Number of Neutrophils (tail)
n.s.
n=27 n=31 n=19 n=23
crRNA zaka - + - +
crRNA flii - - + +
E Zebrafish
TREATMENT:
Tg(lyz:dsRED), neutrophils
DMSO
Anisomycin
crRNA standard
crRNA nlrp1
crRNA zaka
F Zebrafish
Number of Neutrophils
n.s. n.s.
n=20 n=23 n=19 n=19 n=19 n=20
crRNA nlrp1 - - + + - -
crRNA zaka - - - - + +
Anisomycin - + - + - +

Appendix Figure S18 (related to Figures 7 and 8). Zaka mediates the activation of the Nlrp1 inflammasome to regulate zebrafish hematopoiesis. Representative images and number of neutrophils in Zakb- (A, B), Zaka- (C-F) and *nlrp1* crispant (E, F) larvae of 2 dpf obtained by injecting one-cell stage embryos with standard, *zakb*, *zaka* and/or *lrrflip1a/b* crRNAs/Cas9 complexes. Embryos were also treated by bath immersion with 100 µM anisomycin for 1 to 2 dpf in E and F. Fluorescent cells in each reporter line are indicated with arrowheads. Each dot represents one individual and the mean ± SEM for each group is also shown. P values were calculated by one-way ANOVA and Tukey's multiple range test. n.s., non-significant. *P<0.05; **P<0.01; ****P<0.0001.
