# Supplementary material for: ZAKα/P38 kinase signaling pathway regulates hematopoiesis by activating the NLRP1 inflammasome
Source: EMBO Mol Med. 2023 Sep 7;15(10):e18142. doi: 10.15252/emmm.202318142 (PMC10565642; doi:10.15252/emmm.202318142)
Supplement: Supplementary file 2 — Source Data for Figure 1 [file EMMM-15-e18142-s001.zip › Figure_1/1C/Information.PPTX]

## Slide 1
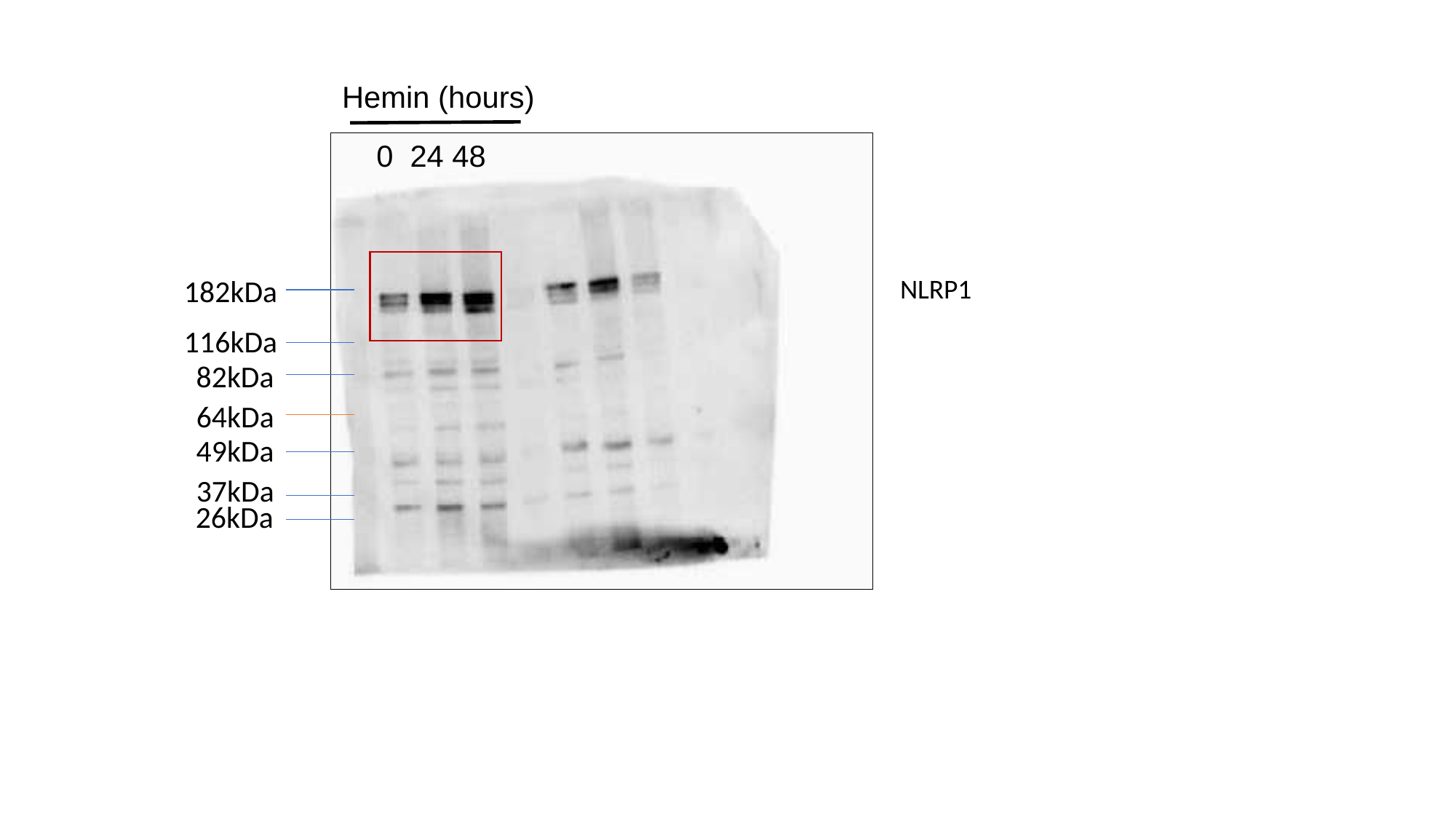

Hemin (hours)
 0 24 48
NLRP1
182kDa
116kDa
82kDa
64kDa
49kDa
37kDa
26kDa

## Slide 2
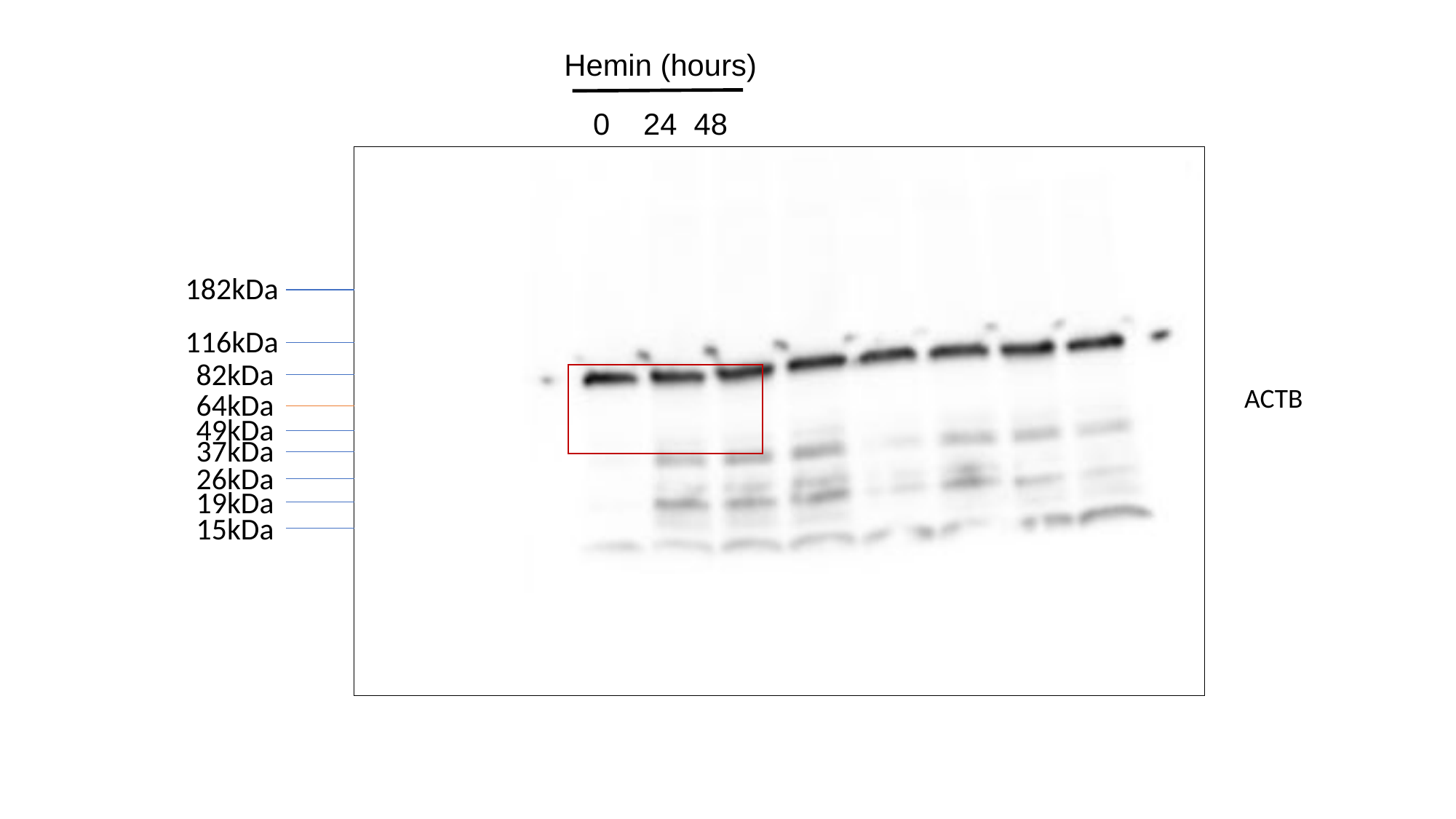

Hemin (hours)
 0 24 48
182kDa
116kDa
82kDa
64kDa
49kDa
37kDa
26kDa
19kDa
15kDa
ACTB
