# Supplementary figures and images for: ZAKα/P38 kinase signaling pathway regulates hematopoiesis by activating the NLRP1 inflammasome
Source: EMBO Mol Med. 2023 Sep 7;15(10):e18142. doi: 10.15252/emmm.202318142 (PMC10565642; doi:10.15252/emmm.202318142)

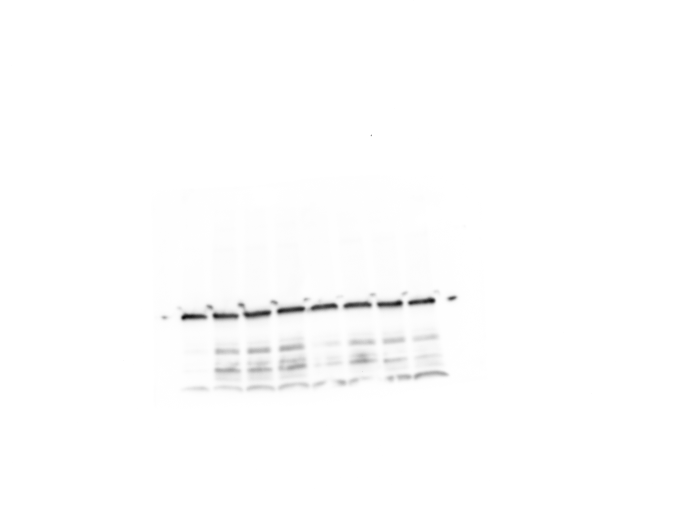

Supplement: Supplementary file 2 — Source Data for Figure 1 [file EMMM-15-e18142-s001.zip › Figure_1/1C/ACTB.tif]

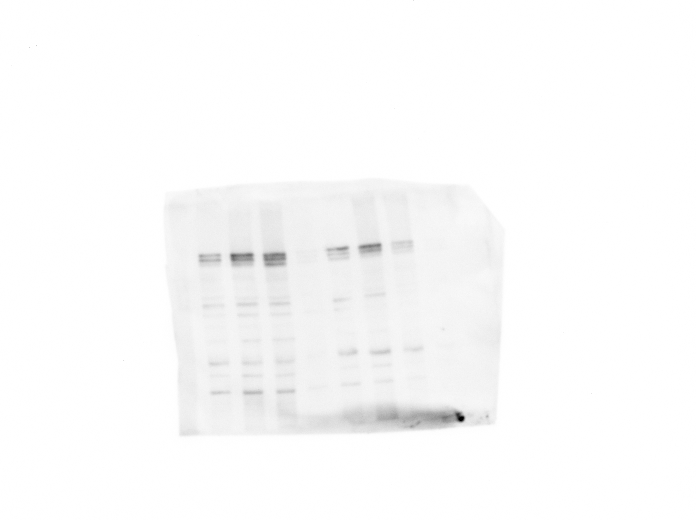

Supplement: Supplementary file 2 — Source Data for Figure 1 [file EMMM-15-e18142-s001.zip › Figure_1/1C/NLRP1.tif]

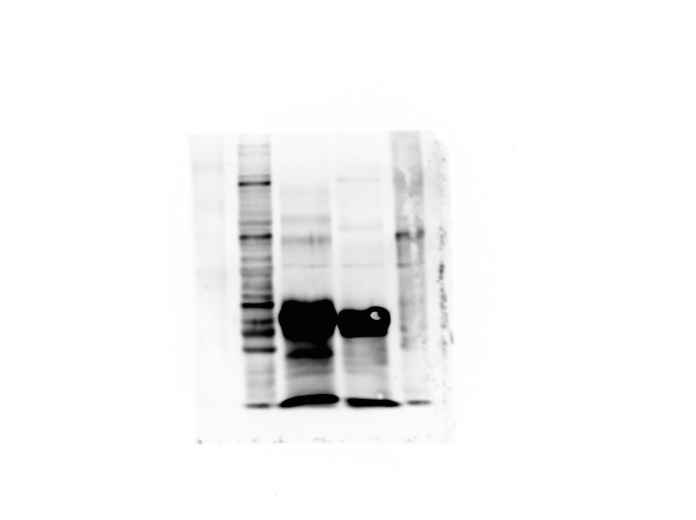

Supplement: Supplementary file 3 — Source Data for Figure 2 [file EMMM-15-e18142-s006.zip › Figure_2/2A/Right/FLII.tif]

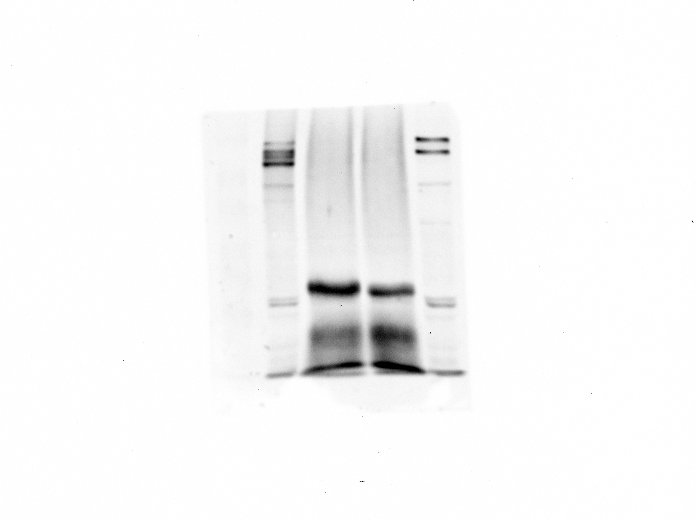

Supplement: Supplementary file 3 — Source Data for Figure 2 [file EMMM-15-e18142-s006.zip › Figure_2/2A/Right/NLRP1.tif]

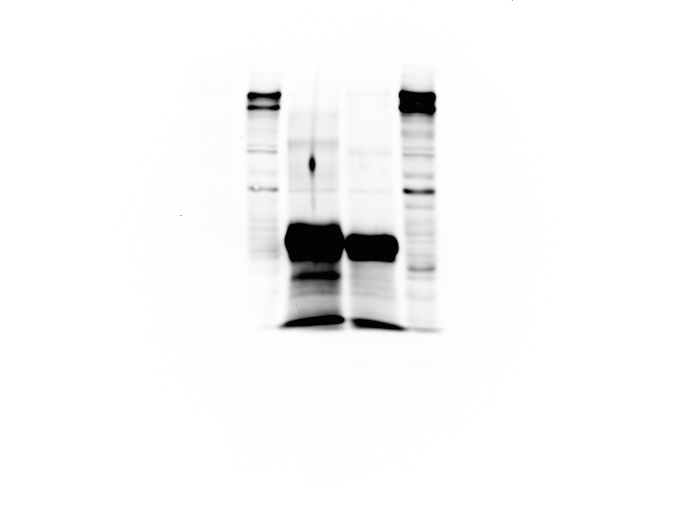

Supplement: Supplementary file 3 — Source Data for Figure 2 [file EMMM-15-e18142-s006.zip › Figure_2/2A/Right/LRRFIP1.tif]

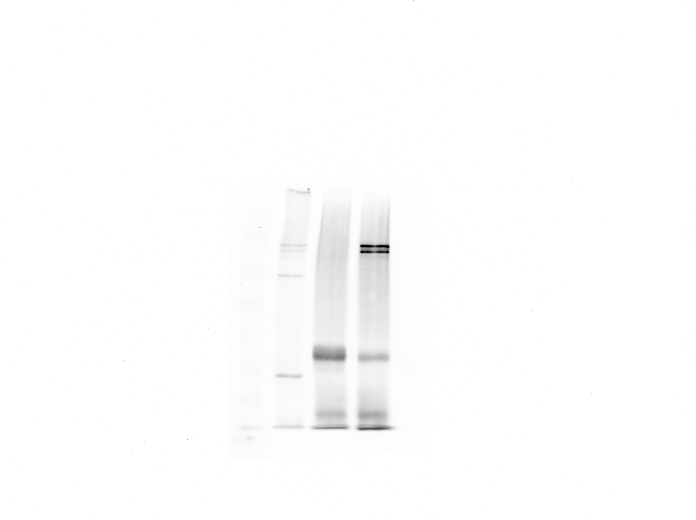

Supplement: Supplementary file 3 — Source Data for Figure 2 [file EMMM-15-e18142-s006.zip › Figure_2/2A/Middle/NLRP1.tif]

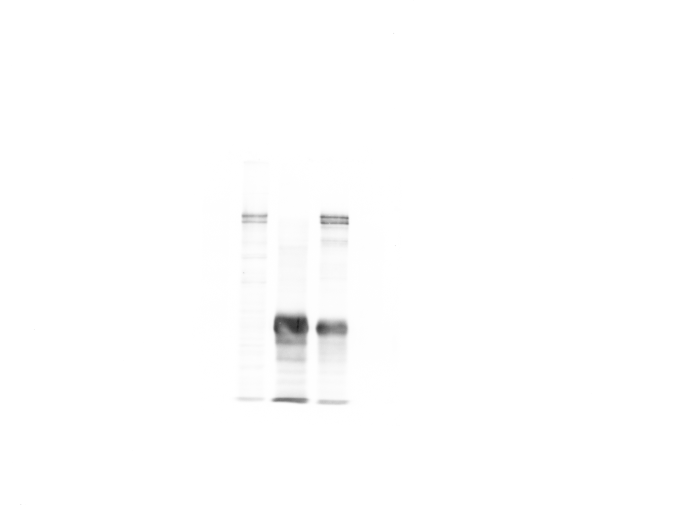

Supplement: Supplementary file 3 — Source Data for Figure 2 [file EMMM-15-e18142-s006.zip › Figure_2/2A/Middle/LRRFIP1.tif]

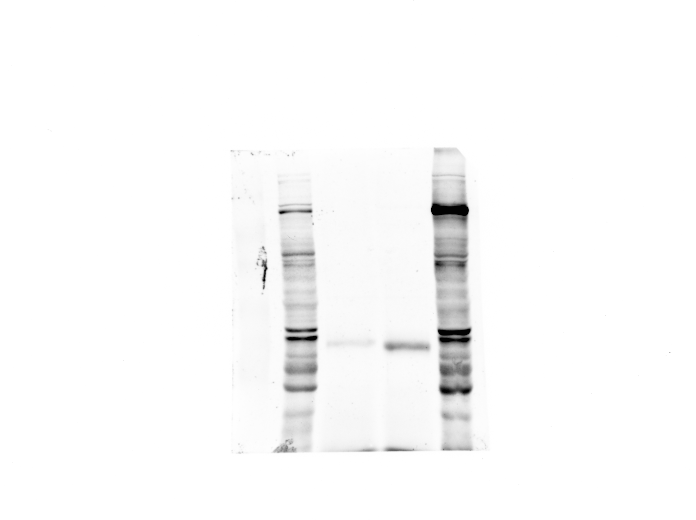

Supplement: Supplementary file 3 — Source Data for Figure 2 [file EMMM-15-e18142-s006.zip › Figure_2/2A/Left/FLII.tif]

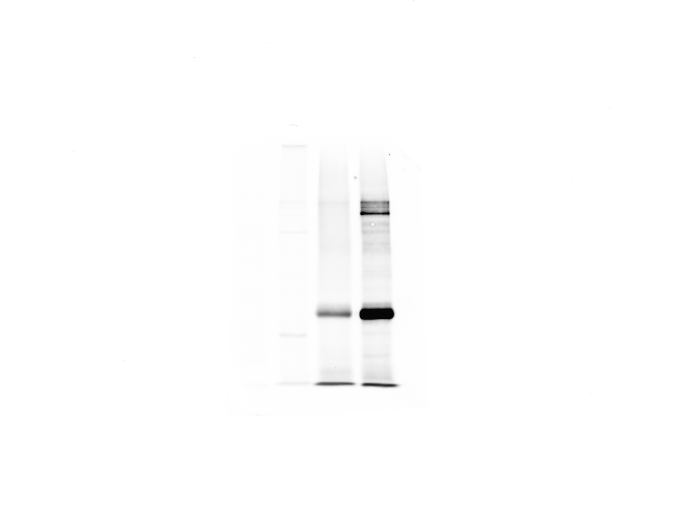

Supplement: Supplementary file 3 — Source Data for Figure 2 [file EMMM-15-e18142-s006.zip › Figure_2/2A/Left/NLRP1.tif]

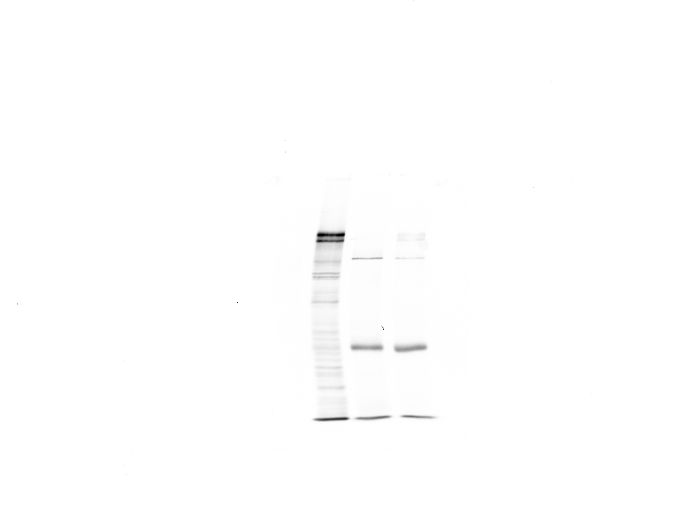

Supplement: Supplementary file 3 — Source Data for Figure 2 [file EMMM-15-e18142-s006.zip › Figure_2/2A/Left/LRRFIP1.tif]

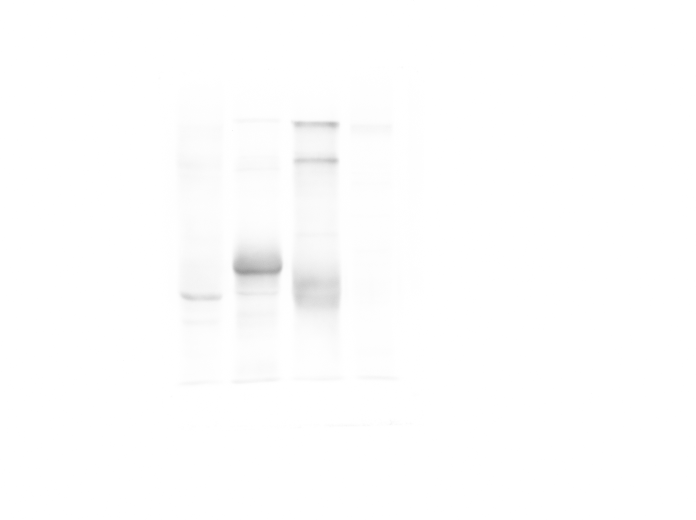

Supplement: Supplementary file 3 — Source Data for Figure 2 [file EMMM-15-e18142-s006.zip › Figure_2/2C/RIGHT/FLII.tif]

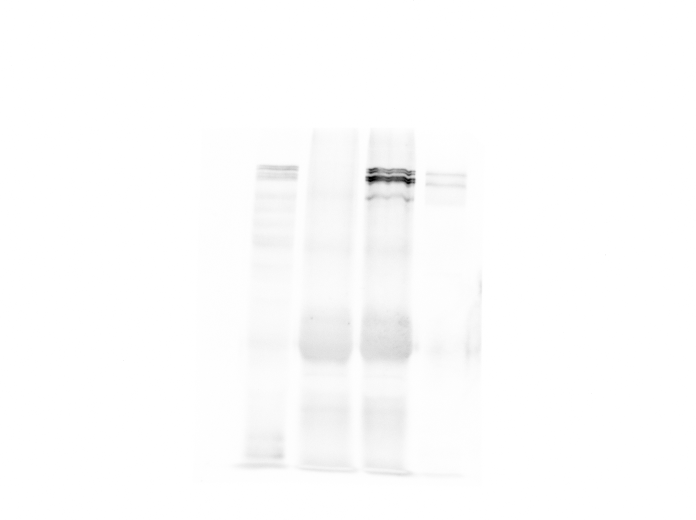

Supplement: Supplementary file 3 — Source Data for Figure 2 [file EMMM-15-e18142-s006.zip › Figure_2/2C/RIGHT/NLRP1.tif]

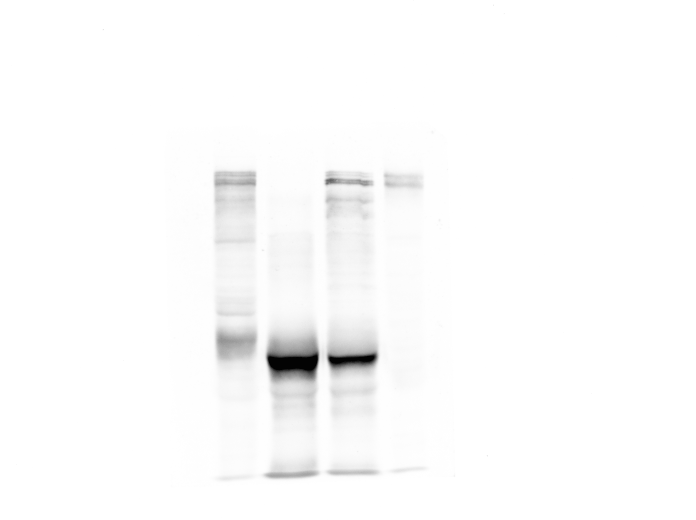

Supplement: Supplementary file 3 — Source Data for Figure 2 [file EMMM-15-e18142-s006.zip › Figure_2/2C/RIGHT/LRRFIP1.tif]

## Slide 1
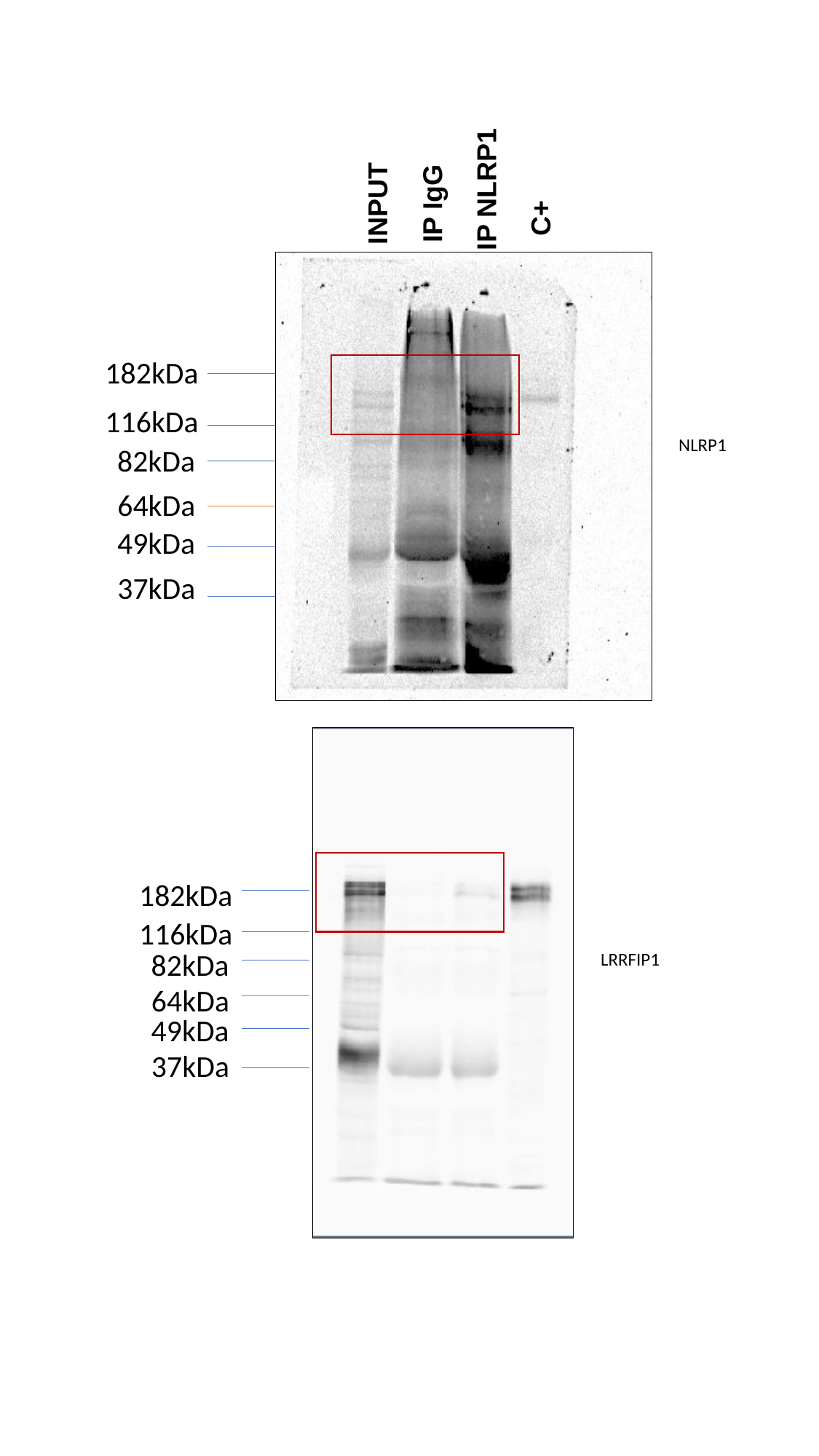

IP NLRP1
INPUT
IP IgG
C+
182kDa
116kDa
82kDa
64kDa
49kDa
37kDa
NLRP1
182kDa
116kDa
82kDa
64kDa
49kDa
37kDa
LRRFIP1

Supplement: Supplementary file 3 — Source Data for Figure 2 [file EMMM-15-e18142-s006.zip › Figure_2/2C/LEFT/Information.pptx]

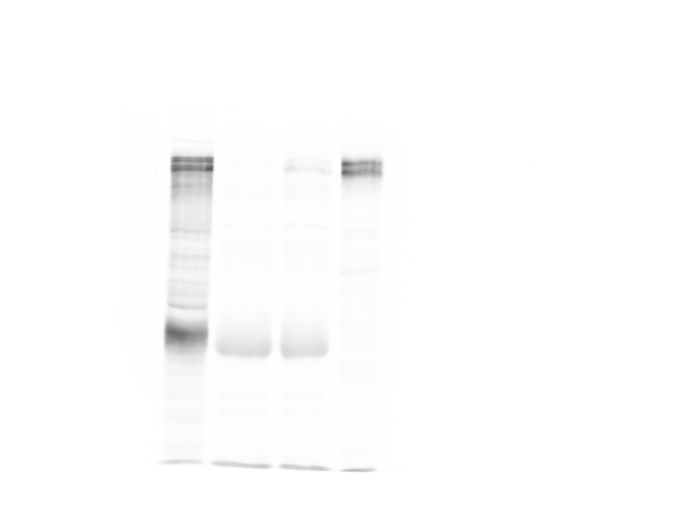

Supplement: Supplementary file 3 — Source Data for Figure 2 [file EMMM-15-e18142-s006.zip › Figure_2/2C/LEFT/LRRFIP1.tif]

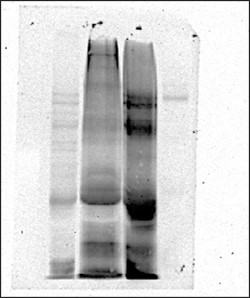

Supplement: Supplementary file 3 — Source Data for Figure 2 [file EMMM-15-e18142-s006.zip › Figure_2/2C/LEFT/NLRP1.jpg]

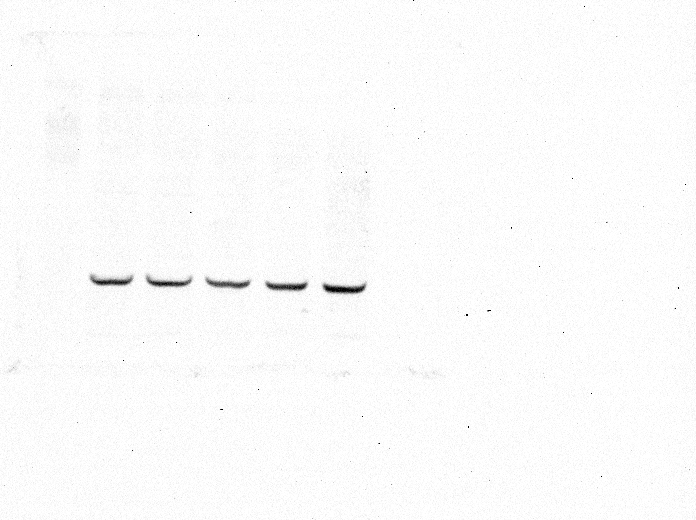

Supplement: Supplementary file 3 — Source Data for Figure 2 [file EMMM-15-e18142-s006.zip › Figure_2/2B/RIGHT/INPUT - ACTB.tif]

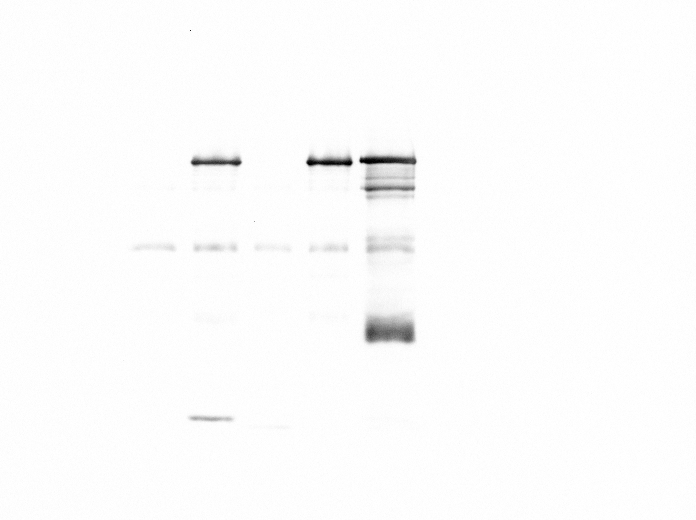

Supplement: Supplementary file 3 — Source Data for Figure 2 [file EMMM-15-e18142-s006.zip › Figure_2/2B/RIGHT/IP FLAG - NLRP1 (FLAG).tif]

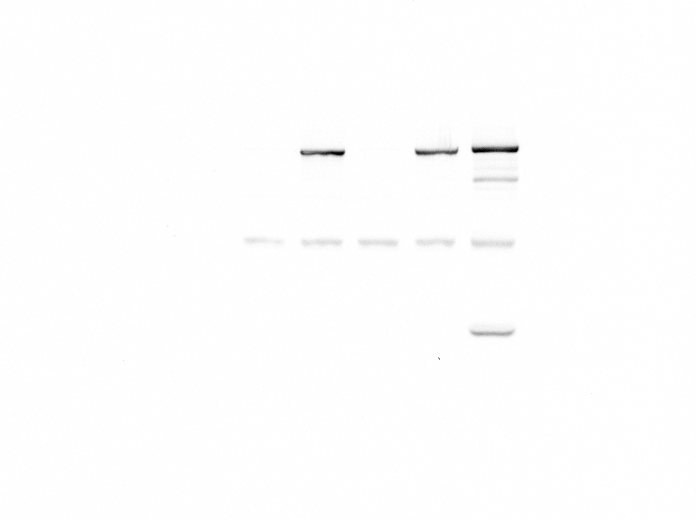

Supplement: Supplementary file 3 — Source Data for Figure 2 [file EMMM-15-e18142-s006.zip › Figure_2/2B/RIGHT/INPUT - NLRP1 (FLAG).tif]

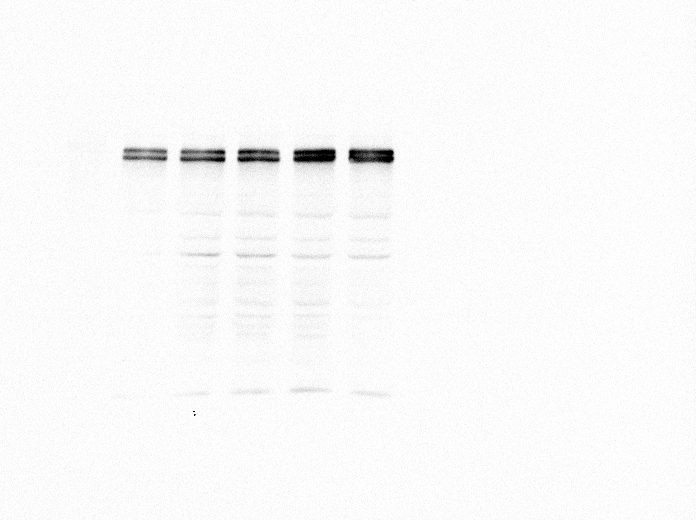

Supplement: Supplementary file 3 — Source Data for Figure 2 [file EMMM-15-e18142-s006.zip › Figure_2/2B/RIGHT/INPUT - LRRFIP1.tif]

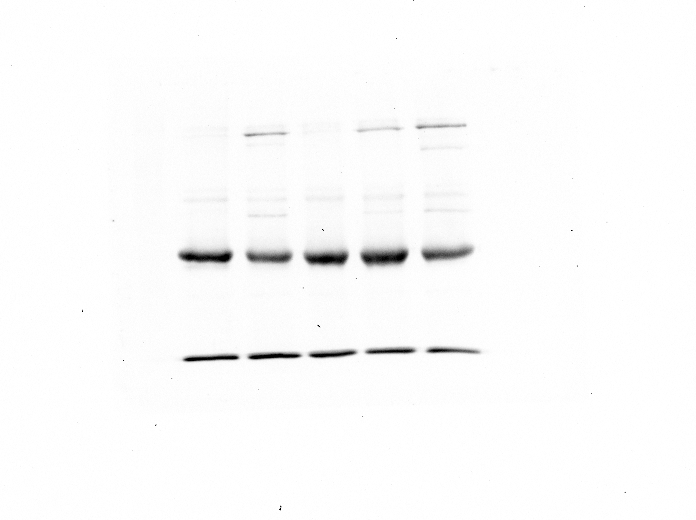

Supplement: Supplementary file 3 — Source Data for Figure 2 [file EMMM-15-e18142-s006.zip › Figure_2/2B/RIGHT/IP FLAG - LRRFIP1.tif]

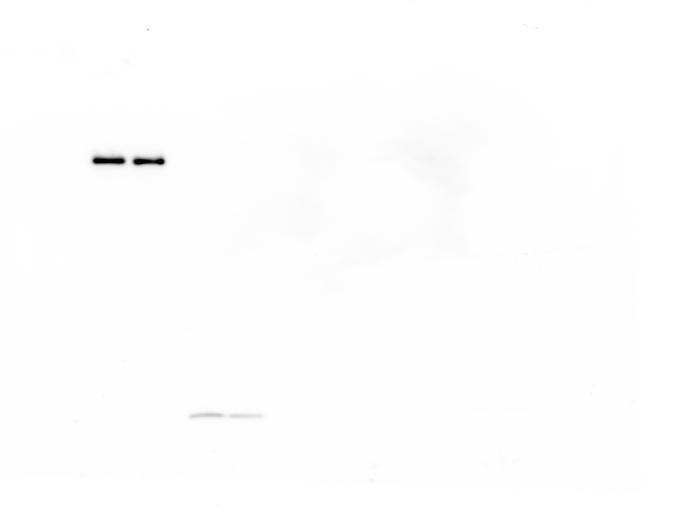

Supplement: Supplementary file 3 — Source Data for Figure 2 [file EMMM-15-e18142-s006.zip › Figure_2/2B/LEFT-MIDDLE/flag IP.tif]

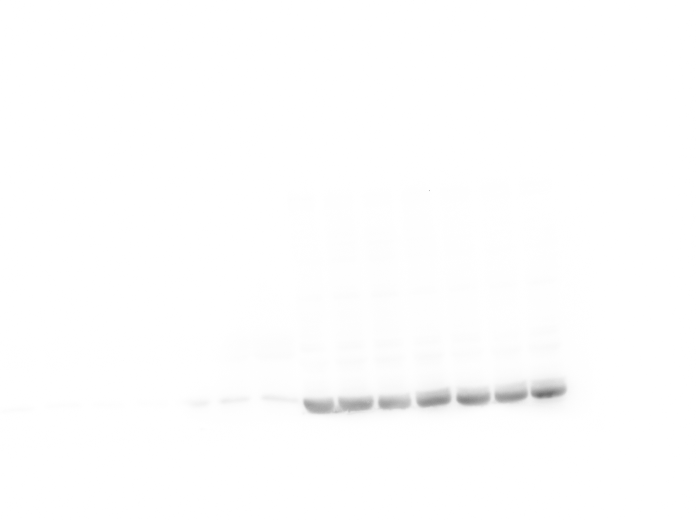

Supplement: Supplementary file 3 — Source Data for Figure 2 [file EMMM-15-e18142-s006.zip › Figure_2/2B/LEFT-MIDDLE/ACTB.tif]

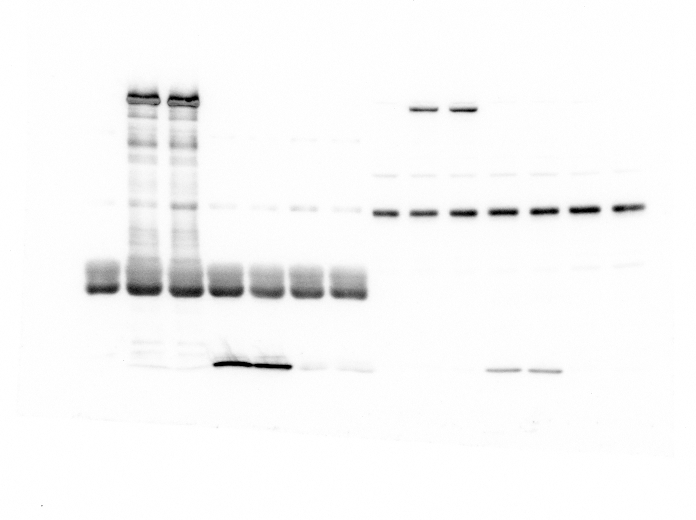

Supplement: Supplementary file 3 — Source Data for Figure 2 [file EMMM-15-e18142-s006.zip › Figure_2/2B/LEFT-MIDDLE/flag INPUT.tif]

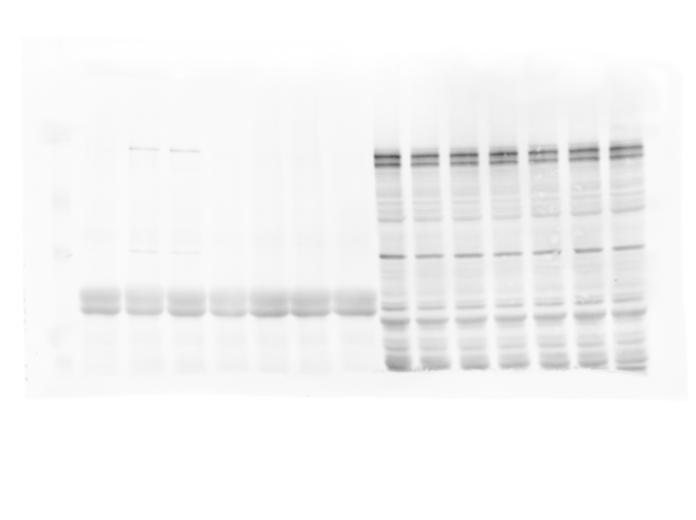

Supplement: Supplementary file 3 — Source Data for Figure 2 [file EMMM-15-e18142-s006.zip › Figure_2/2B/LEFT-MIDDLE/LRRFIP1.tif]

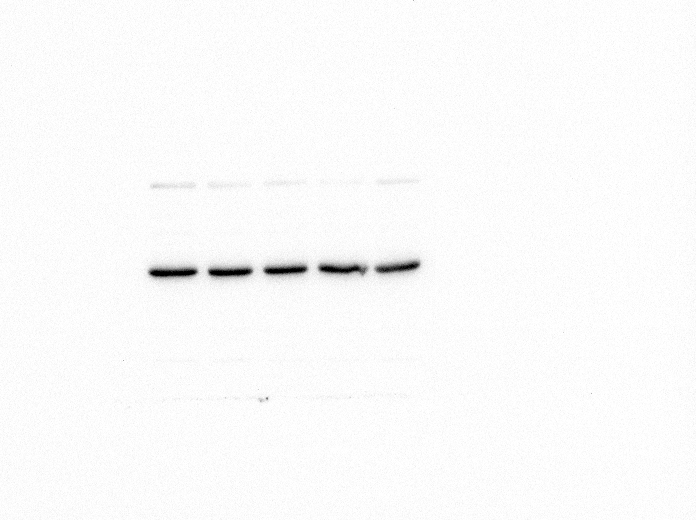

Supplement: Supplementary file 4 — Source Data for Figure 3 [file EMMM-15-e18142-s004.zip › Figure_3/3E/ACTB.tif]

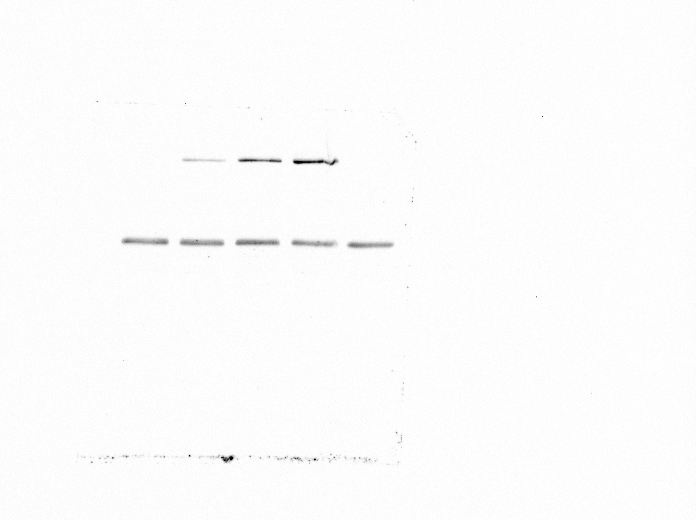

Supplement: Supplementary file 4 — Source Data for Figure 3 [file EMMM-15-e18142-s004.zip › Figure_3/3E/FLII (FLAG).tif]

## Slide 1
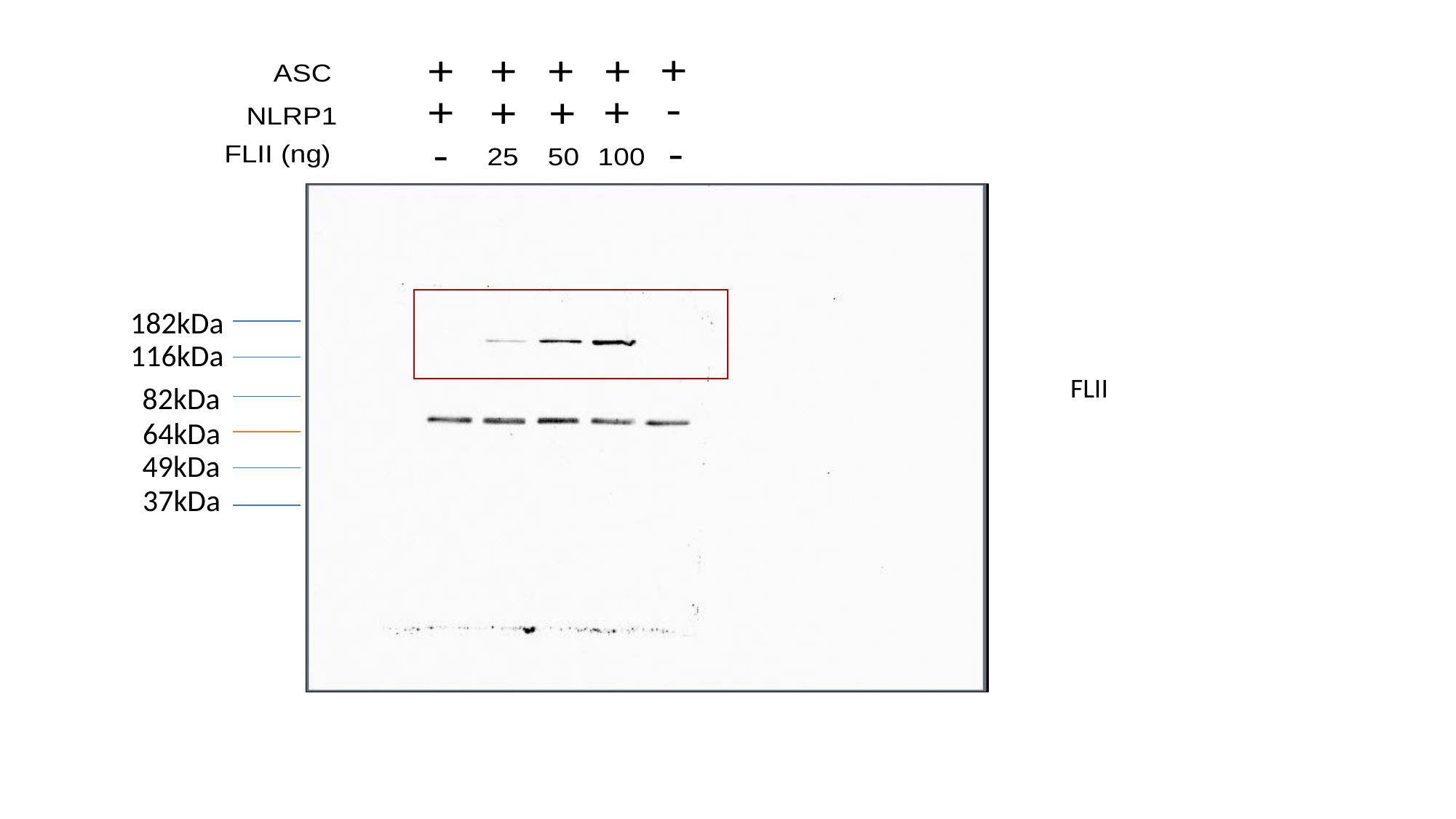

182kDa
116kDa
82kDa
64kDa
49kDa
37kDa
FLII

## Slide 2
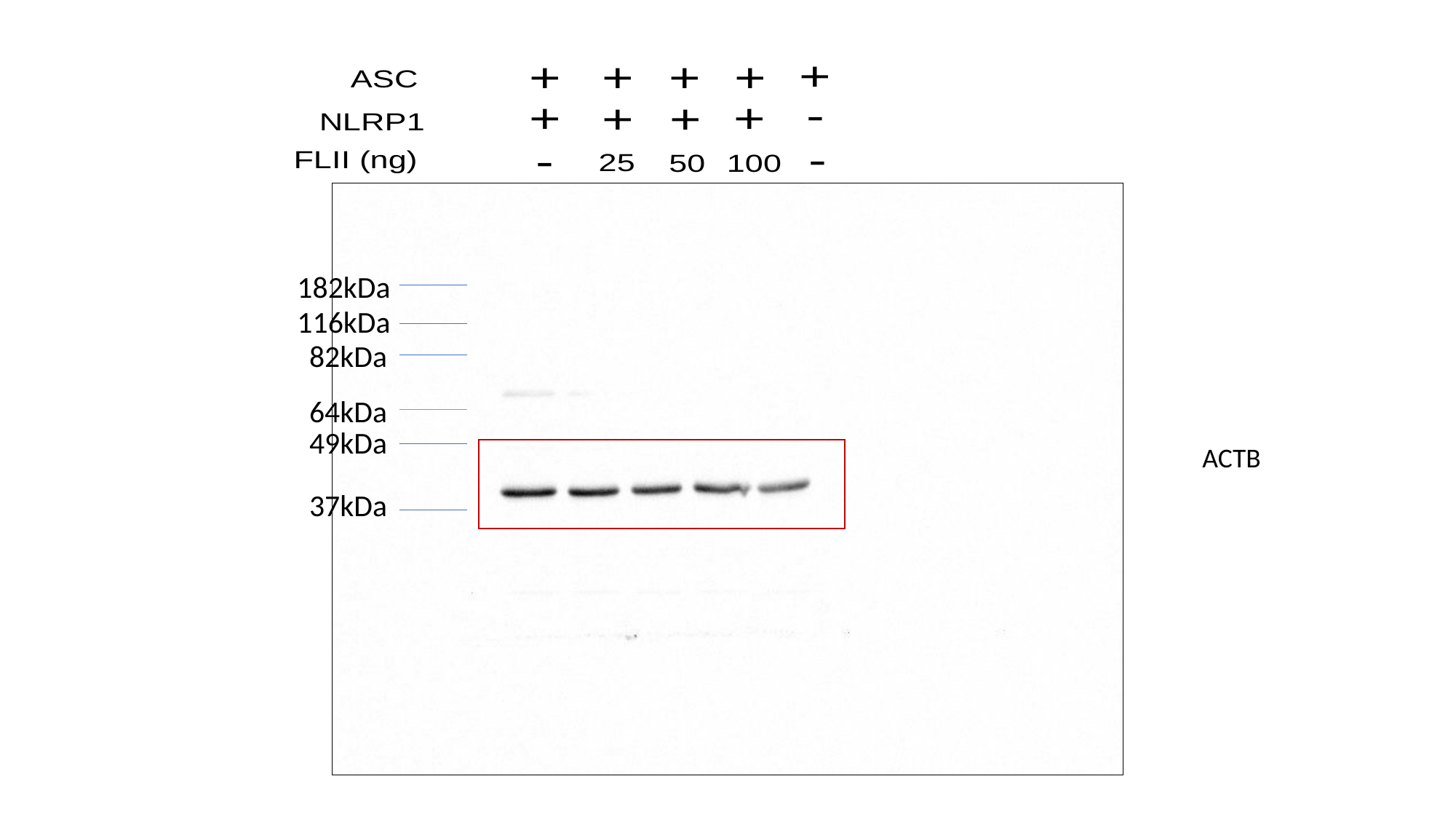

182kDa
116kDa
82kDa
64kDa
49kDa
37kDa
ACTB

Supplement: Supplementary file 4 — Source Data for Figure 3 [file EMMM-15-e18142-s004.zip › Figure_3/3E/Information.PPTX]

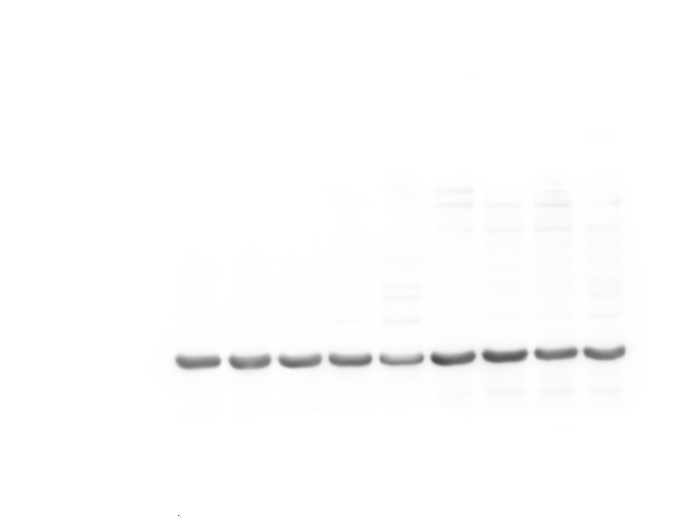

Supplement: Supplementary file 4 — Source Data for Figure 3 [file EMMM-15-e18142-s004.zip › Figure_3/3D/ACTB.tif]

## Slide 1
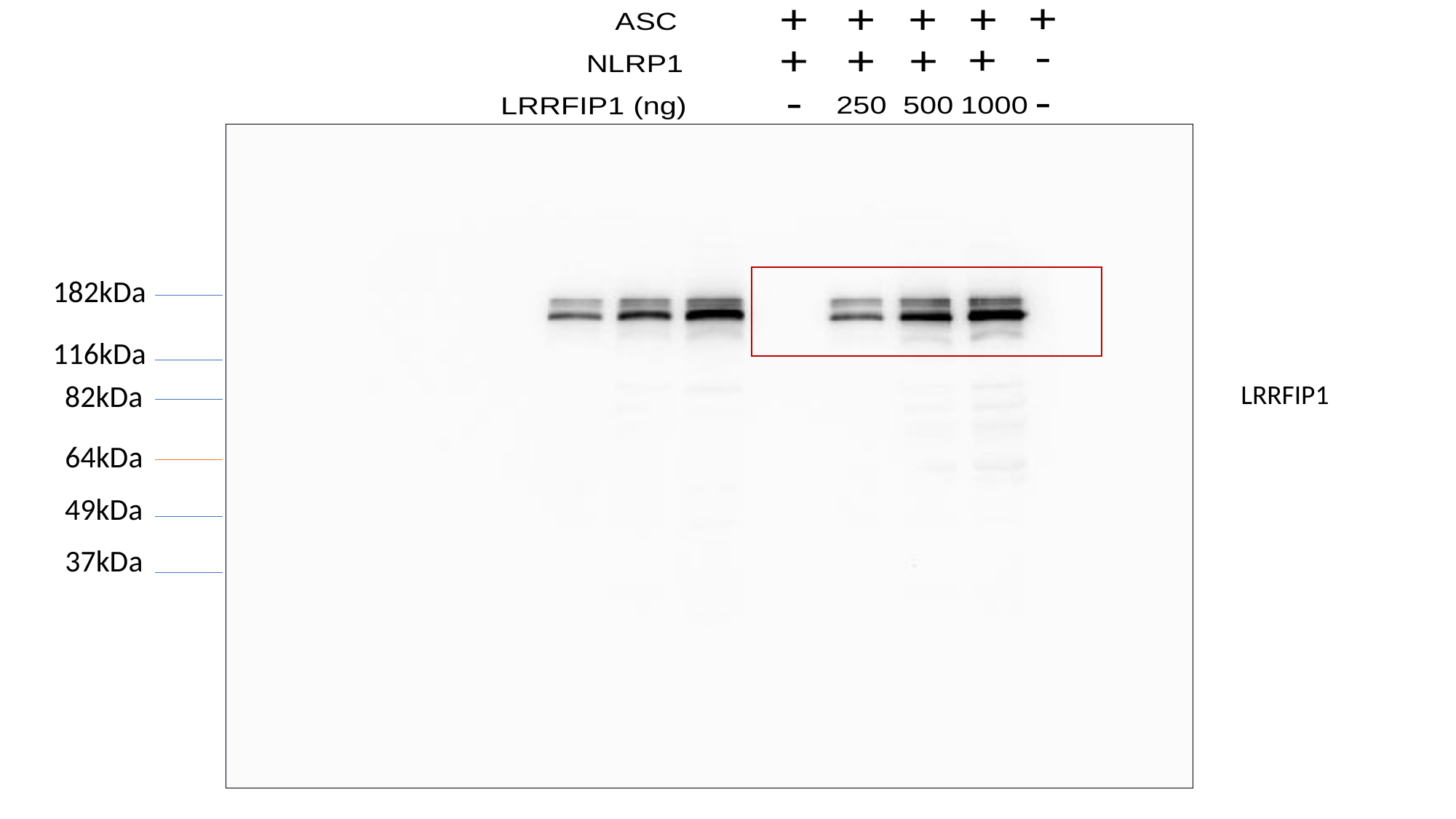

182kDa
116kDa
82kDa
64kDa
49kDa
37kDa
LRRFIP1

## Slide 2
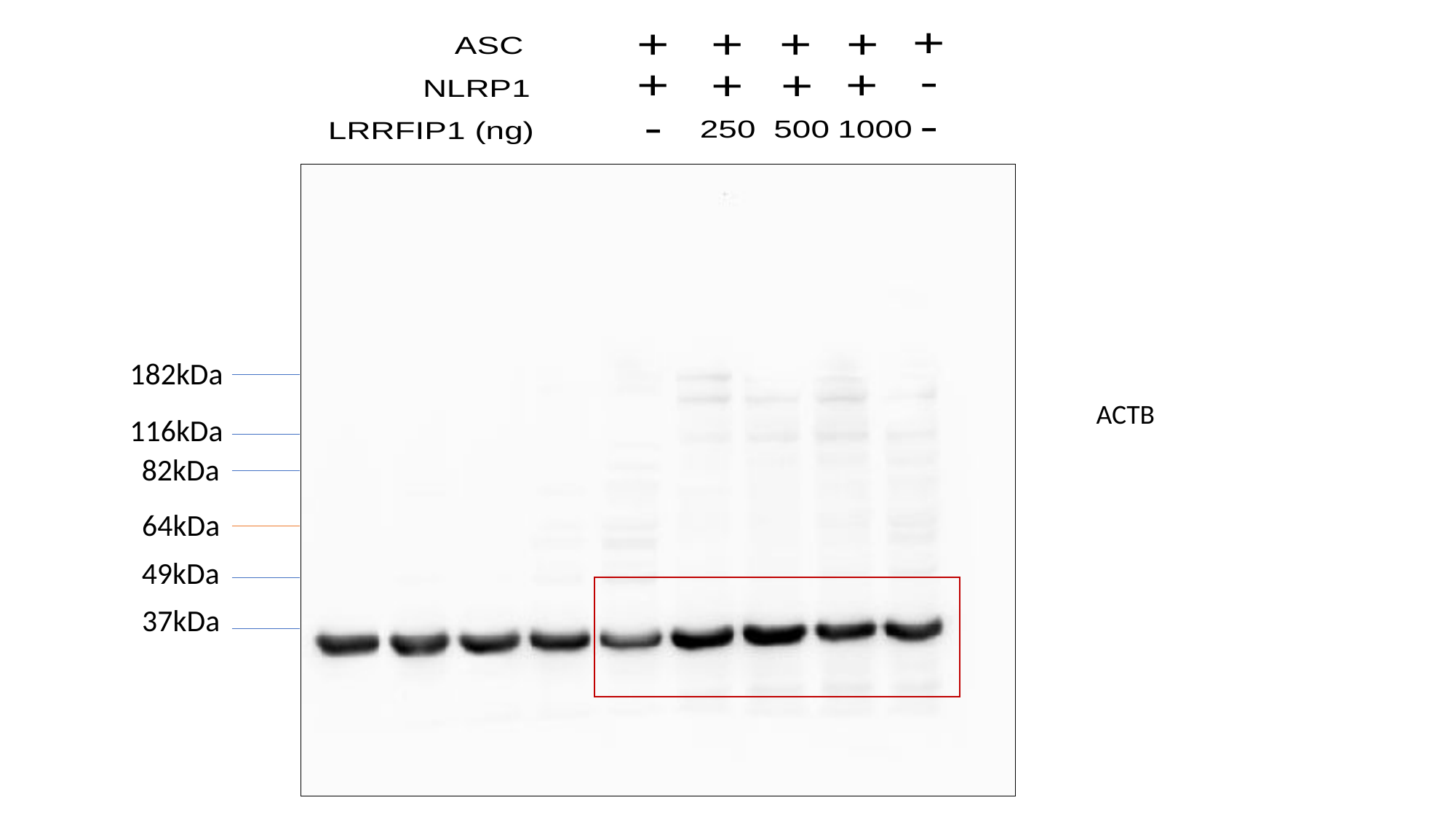

182kDa
116kDa
82kDa
64kDa
49kDa
37kDa
ACTB

Supplement: Supplementary file 4 — Source Data for Figure 3 [file EMMM-15-e18142-s004.zip › Figure_3/3D/Information.PPTX]

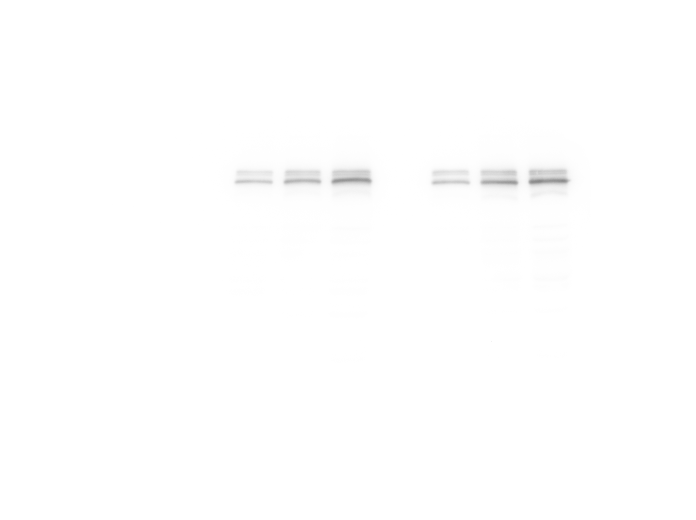

Supplement: Supplementary file 4 — Source Data for Figure 3 [file EMMM-15-e18142-s004.zip › Figure_3/3D/LRRFIP1.tif]

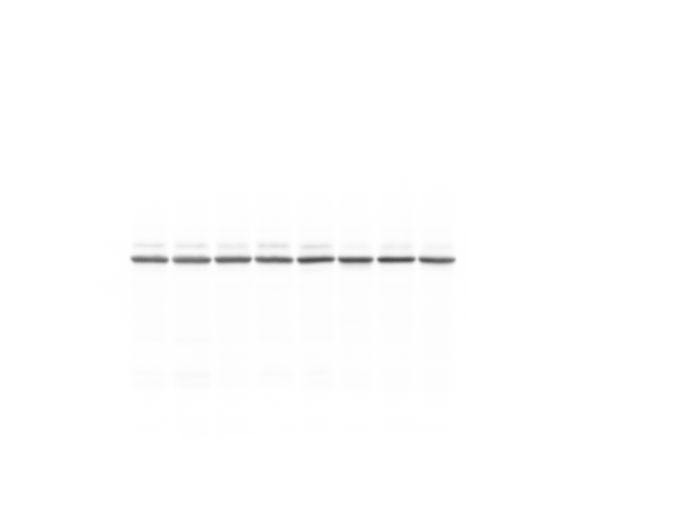

Supplement: Supplementary file 7 — Source Data for Figure 6 [file EMMM-15-e18142-s010.zip › Figure_6/6A/ACTB.tif]

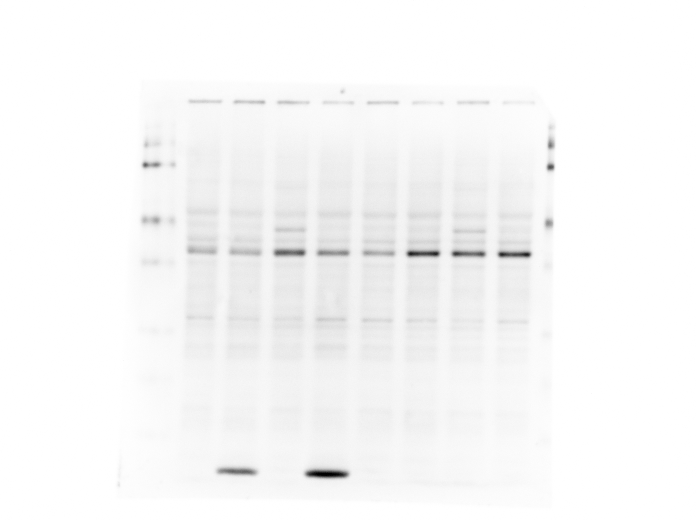

Supplement: Supplementary file 7 — Source Data for Figure 6 [file EMMM-15-e18142-s010.zip › Figure_6/6A/fosfo P38.tif]

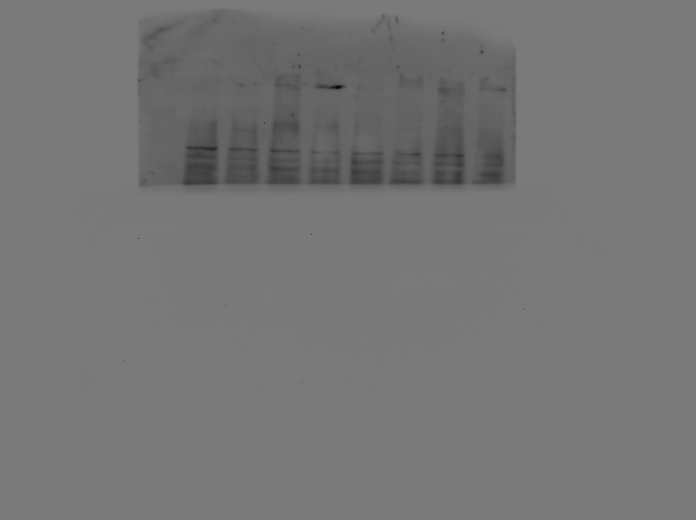

Supplement: Supplementary file 7 — Source Data for Figure 6 [file EMMM-15-e18142-s010.zip › Figure_6/6A/NLRP1.tif]

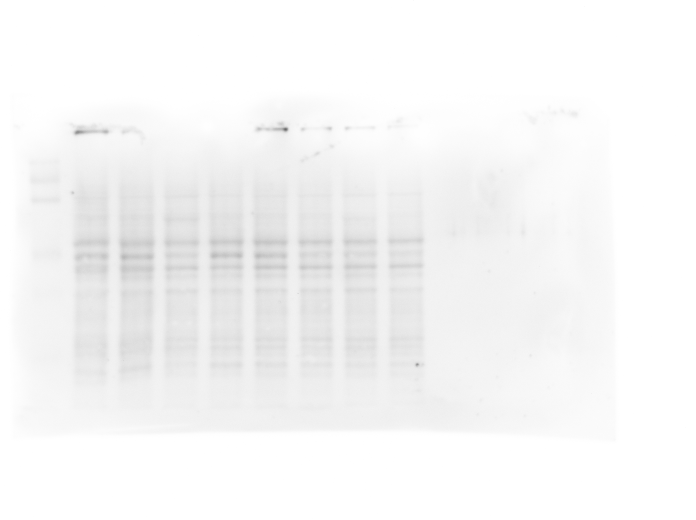

Supplement: Supplementary file 7 — Source Data for Figure 6 [file EMMM-15-e18142-s010.zip › Figure_6/6A/GATA1.tif]

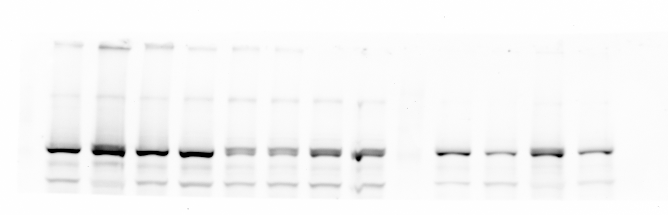

Supplement: Supplementary file 7 — Source Data for Figure 6 [file EMMM-15-e18142-s010.zip › Figure_6/6A/ZAKalfa.tif]

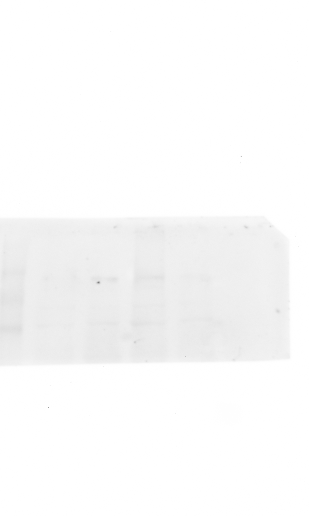

Supplement: Supplementary file 7 — Source Data for Figure 6 [file EMMM-15-e18142-s010.zip › Figure_6/6B/NLRP1.tif]

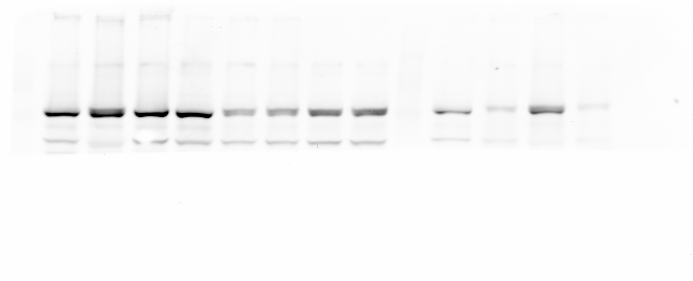

Supplement: Supplementary file 7 — Source Data for Figure 6 [file EMMM-15-e18142-s010.zip › Figure_6/6B/ZAKalfa.tif]

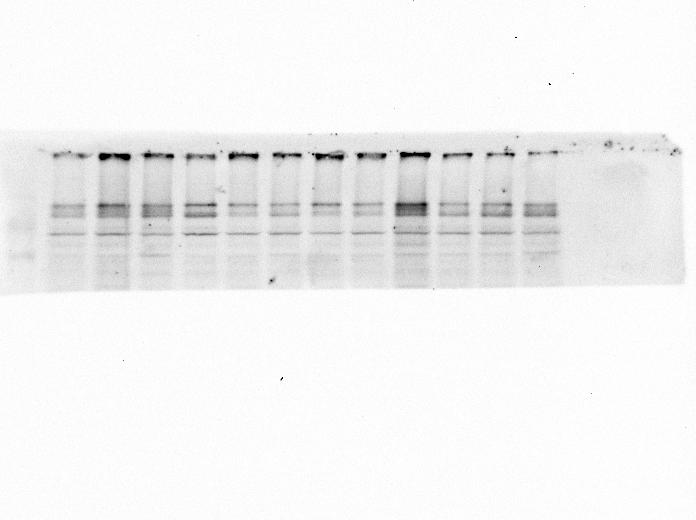

Supplement: Supplementary file 7 — Source Data for Figure 6 [file EMMM-15-e18142-s010.zip › Figure_6/6C/NLRP1.tif]

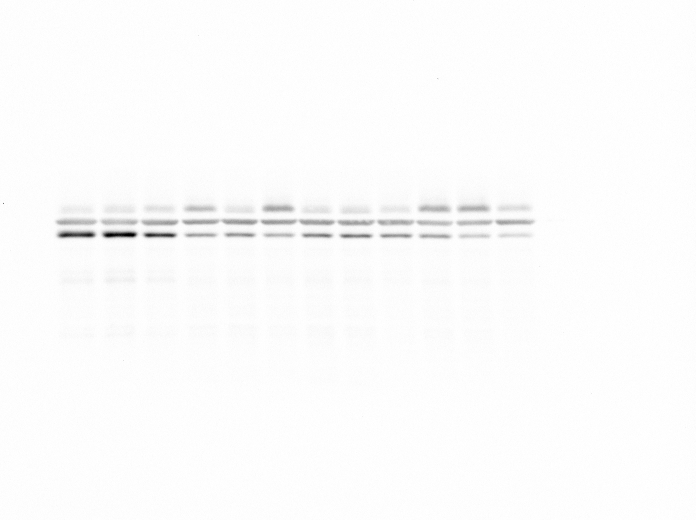

Supplement: Supplementary file 7 — Source Data for Figure 6 [file EMMM-15-e18142-s010.zip › Figure_6/6C/p38 fosfo.tif]

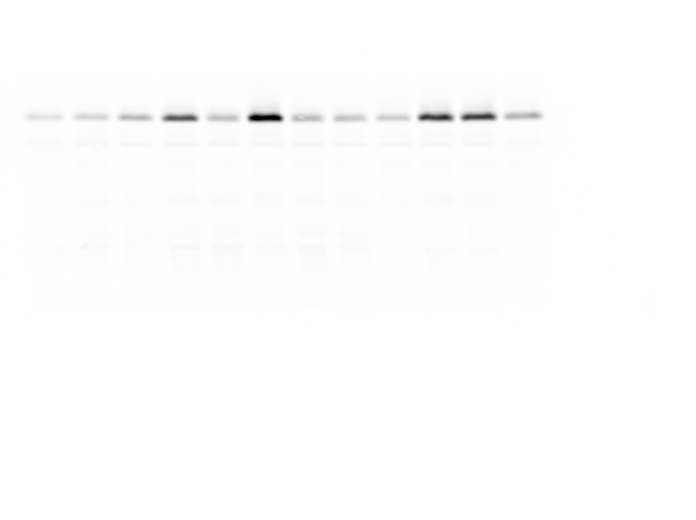

Supplement: Supplementary file 7 — Source Data for Figure 6 [file EMMM-15-e18142-s010.zip › Figure_6/6C/GATA1.tif]

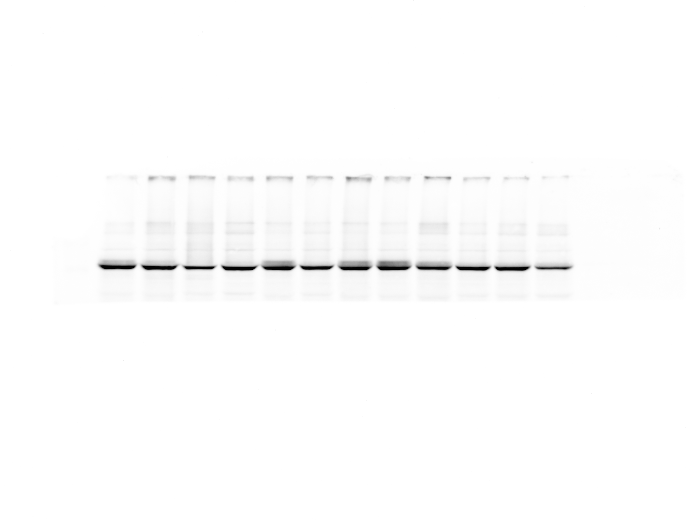

Supplement: Supplementary file 7 — Source Data for Figure 6 [file EMMM-15-e18142-s010.zip › Figure_6/6C/ZAKalfa.tif]
