# Supplementary material for: ZAKα/P38 kinase signaling pathway regulates hematopoiesis by activating the NLRP1 inflammasome
Source: EMBO Mol Med. 2023 Sep 7;15(10):e18142. doi: 10.15252/emmm.202318142 (PMC10565642; doi:10.15252/emmm.202318142)
Supplement: Supplementary file 3 — Source Data for Figure 2 [file EMMM-15-e18142-s006.zip › Figure_2/2A/Left/Information.PPTX]

## Slide 1
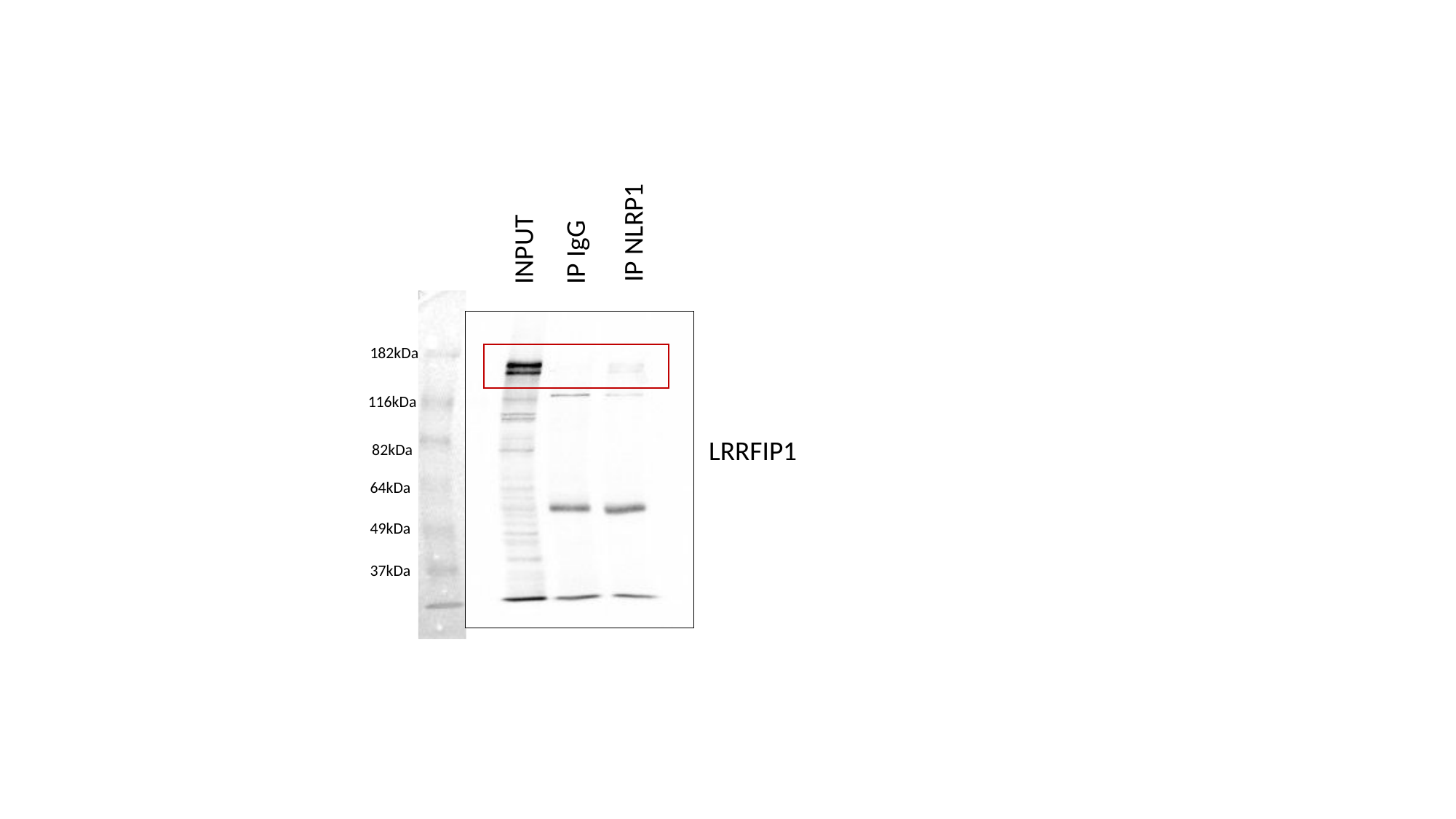

IP NLRP1
INPUT
IP IgG
182kDa
116kDa
LRRFIP1
82kDa
64kDa
49kDa
37kDa

## Slide 2
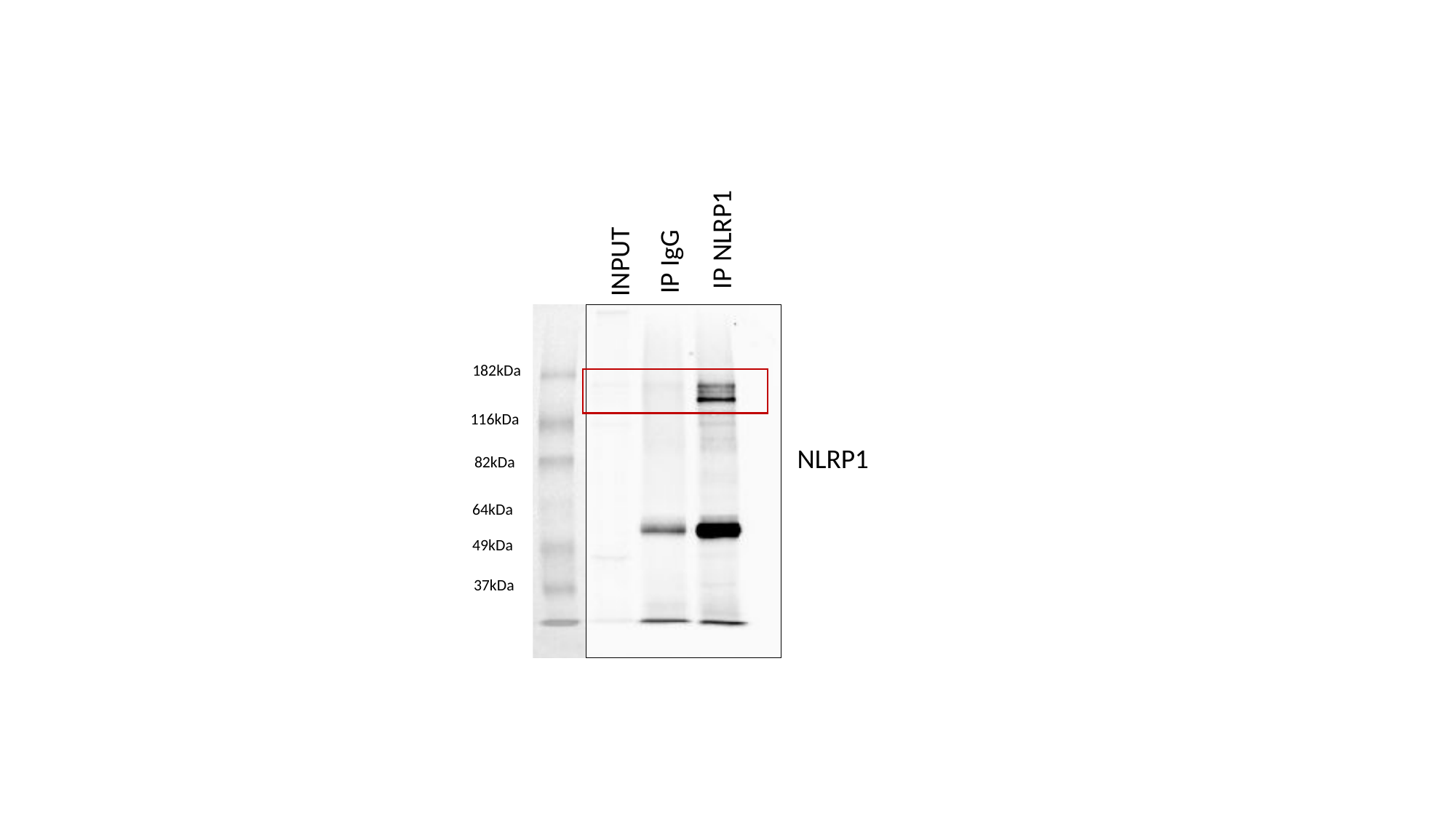

IP NLRP1
INPUT
IP IgG
182kDa
116kDa
NLRP1
82kDa
64kDa
49kDa
37kDa

## Slide 3
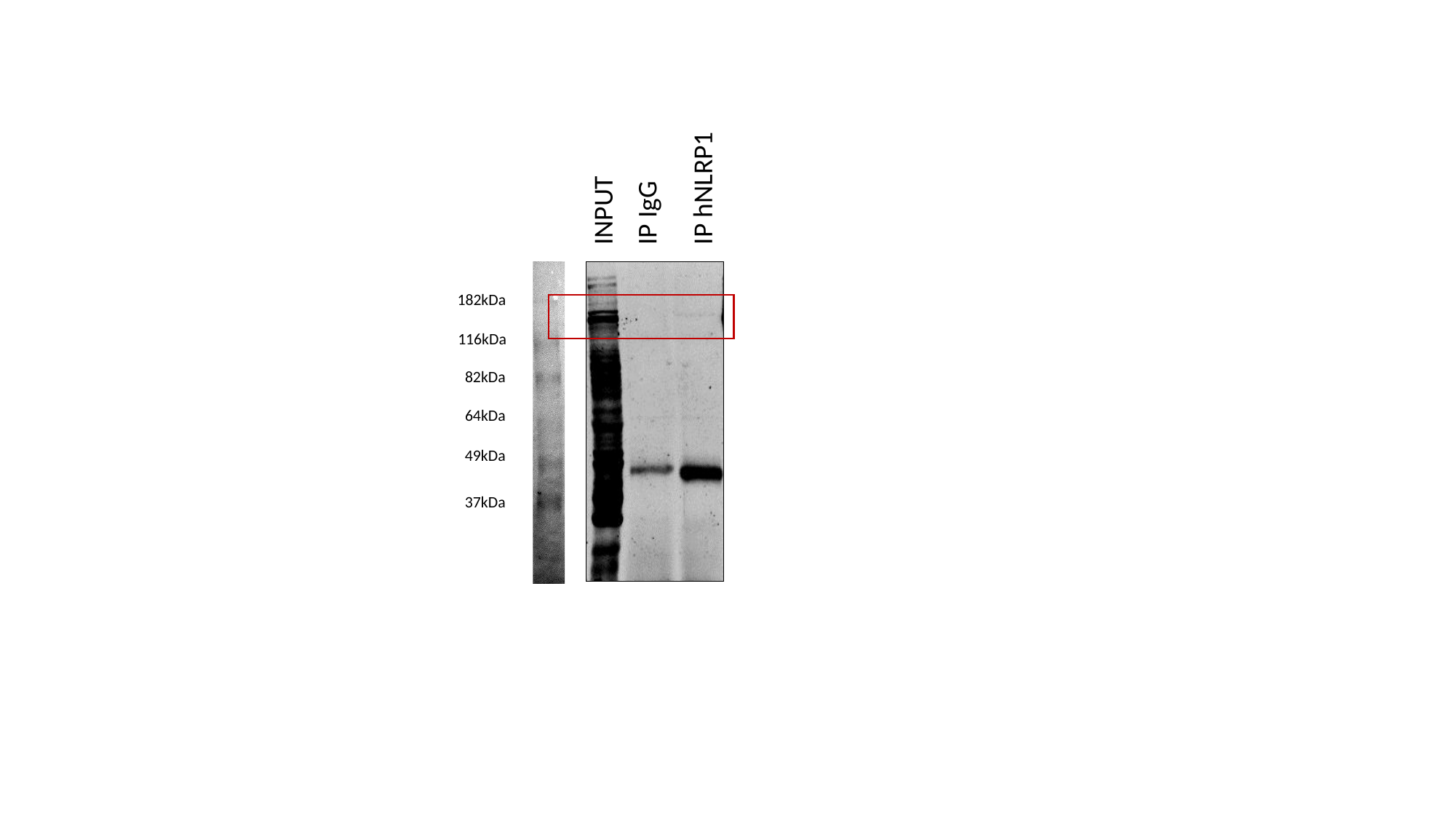

IP hNLRP1
INPUT
IP IgG
182kDa
116kDa
82kDa
64kDa
49kDa
37kDa
