# Supplementary material for: ZAKα/P38 kinase signaling pathway regulates hematopoiesis by activating the NLRP1 inflammasome
Source: EMBO Mol Med. 2023 Sep 7;15(10):e18142. doi: 10.15252/emmm.202318142 (PMC10565642; doi:10.15252/emmm.202318142)
Supplement: Supplementary file 7 — Source Data for Figure 6 [file EMMM-15-e18142-s010.zip › Figure_6/6B/Information.PPTX]

## Slide 1
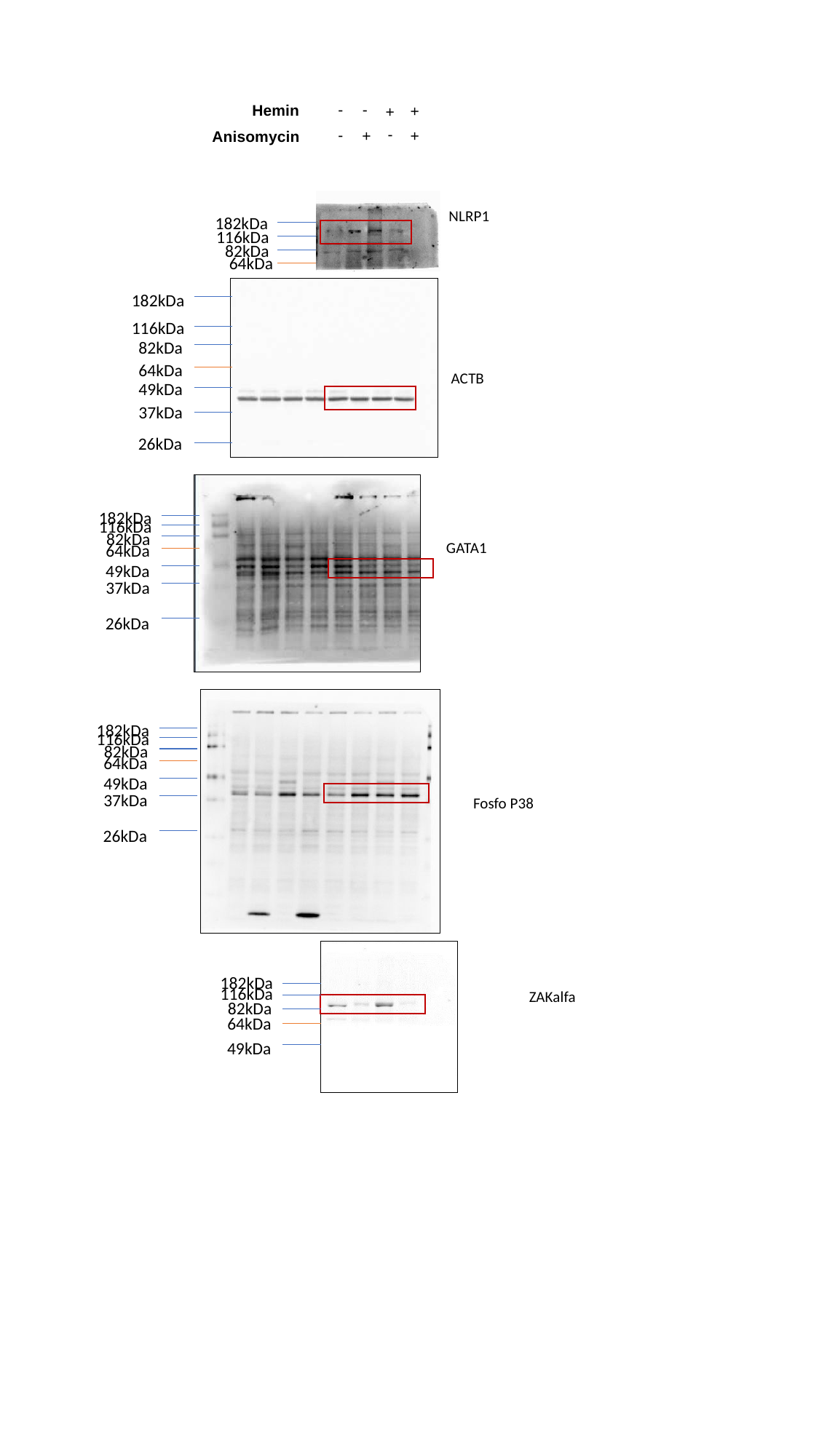

-
-
Hemin
+
+
-
-
+
+
Anisomycin
NLRP1
182kDa
116kDa
82kDa
64kDa
182kDa
116kDa
82kDa
64kDa
49kDa
37kDa
26kDa
ACTB
182kDa
116kDa
82kDa
64kDa
49kDa
37kDa
26kDa
GATA1
182kDa
116kDa
82kDa
64kDa
49kDa
37kDa
26kDa
Fosfo P38
182kDa
116kDa
82kDa
64kDa
49kDa
ZAKalfa
