# Supplementary material for: ZAKα/P38 kinase signaling pathway regulates hematopoiesis by activating the NLRP1 inflammasome
Source: EMBO Mol Med. 2023 Sep 7;15(10):e18142. doi: 10.15252/emmm.202318142 (PMC10565642; doi:10.15252/emmm.202318142)
Supplement: Supplementary file 7 — Source Data for Figure 6 [file EMMM-15-e18142-s010.zip › Figure_6/6C/Information.PPTX]

## Slide 1
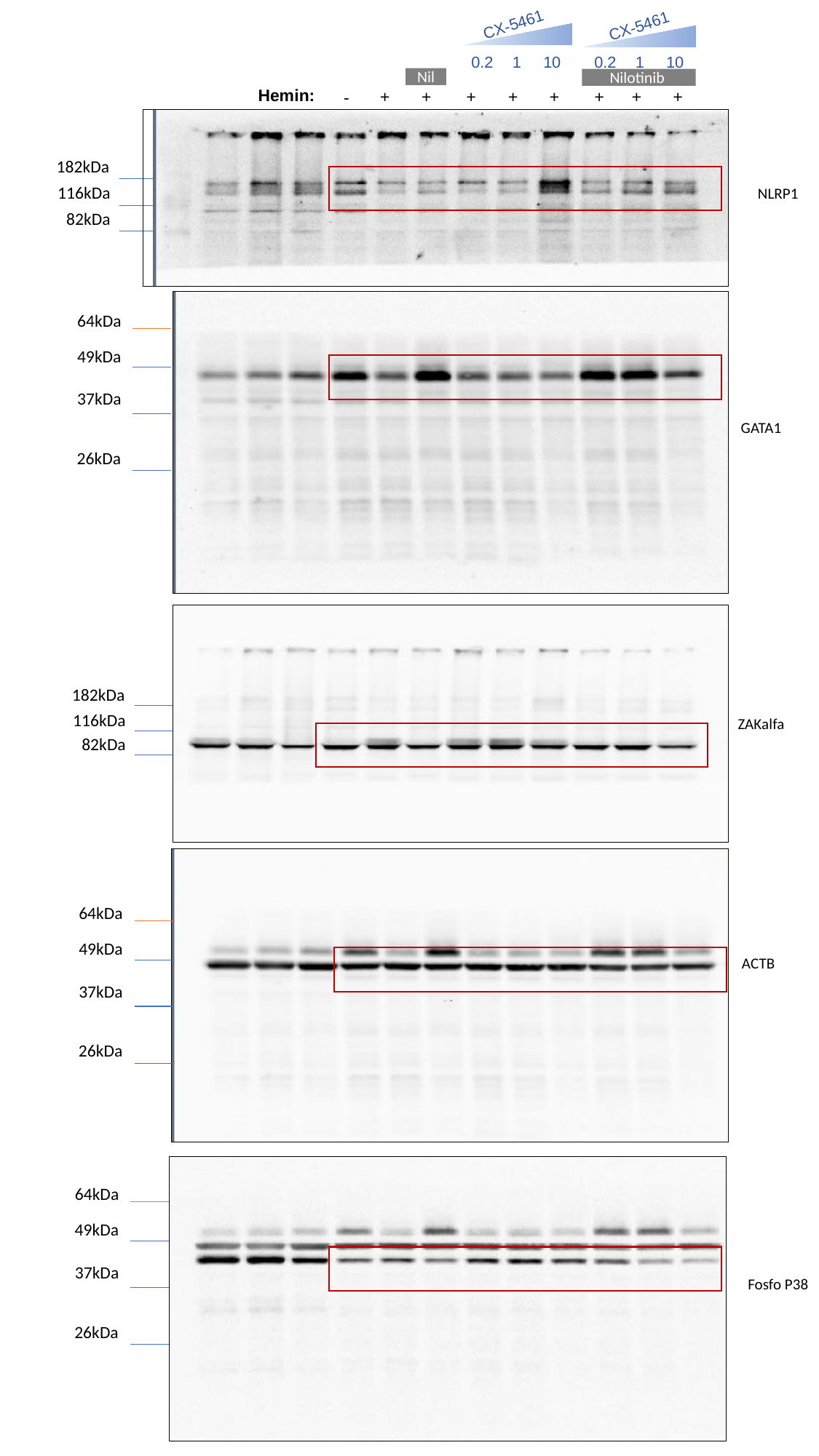

CX-5461
CX-5461
0.2
1
10
0.2
1
10
Nil
Nilotinib
Hemin:
-
+
+
+
+
+
+
+
+
182kDa
116kDa
82kDa
NLRP1
64kDa
49kDa
37kDa
26kDa
GATA1
182kDa
116kDa
82kDa
ZAKalfa
64kDa
49kDa
37kDa
26kDa
ACTB
64kDa
49kDa
37kDa
26kDa
Fosfo P38
