# Supplementary material for: ZAKα/P38 kinase signaling pathway regulates hematopoiesis by activating the NLRP1 inflammasome
Source: EMBO Mol Med. 2023 Sep 7;15(10):e18142. doi: 10.15252/emmm.202318142 (PMC10565642; doi:10.15252/emmm.202318142)
Supplement: Supplementary file 10 [file EMMM-15-e18142-s009.pdf]

EMBO Molecular Medicine

ZAKa/P38 kinase signaling pathway regulates hematopoiesis by activating the NLRP1 inflammasome

Lola Rodriguez-Ruiz, Juan Lozano-Gil, Elena Naranjo-Sánchez, Elena Martínez-Balsalobre, Alicia Martinez-Lopez, Christophe Lachaud, Miguel Blanquer, Toan Phung, Diana García-Moreno, Maria L. Cayuela, Sylwia Tyrkalska, Ana Perez-Oliva, and Victoriano Mulero
DOI: 10.15252/emmm.202318142

Corresponding author(s): Victoriano Mulero (vmulero@um.es) , Sylwia Tyrkalska (tyrkalska.sylwia@gmail.com), Ana Perez-Oliva (anab.perez@imib.es)

Review Timeline:

Submission Date:	9th Jun 23
Editorial Decision:	12th Jul 23
Revision Received:	20th Jul 23
Editorial Decision:	15th Aug 23
Revision Received:	16th Aug 23
Accepted:	22nd Aug 23

Editor: Zeljko Durdevic

Transaction Report:

(Note: With the exception of the correction of typographical or spelling errors that could be a source of ambiguity, letters and reports are not edited. Depending on transfer agreements, referee reports obtained elsewhere may or may not be included in this compilation. Referee reports are anonymous unless the Referee chooses to sign their reports.)

12th Jul 2023

Dear Prof. Mulero,

Thank you for the submission of your manuscript to EMBO Molecular Medicine. We have now received feedback from the two reviewers who agreed to evaluate your manuscript. Both referees recognize potential interest of the study but also raise important criticism that should be addressed in a major revision. If you would like to discuss further the points raised by the referees, I am available to do so via email or video. Let me know if you are interested in this option.

Further consideration of a revision that addresses reviewers' concerns in full will entail a second round of review. EMBO Molecular Medicine encourages a single round of revision only and therefore, acceptance or rejection of the manuscript will depend on the completeness of your responses included in the next, final version of the manuscript. For this reason, and to save you from any frustrations in the end, I would strongly advise against returning an incomplete revision.

We would welcome the submission of a revised version within three months for further consideration. Please let us know if you require longer to complete the revision.

I look forward to receiving your revised manuscript.

Yours sincerely,

Zeljko Durdevic

Zeljko Durdevic
Editor
EMBO Molecular Medicine

*****

When submitting your revised manuscript, please carefully review the instructions that follow below. We perform an initial quality control of all revised manuscripts before re-review; failure to include requested items will delay the evaluation of your revision.

We require:

1) A .docx formatted version of the manuscript text (including legends for main figures, EV figures and tables). Please make sure that the changes are highlighted to be clearly visible.

2) Individual production quality figure files as .eps, .tif, .jpg (one file per figure). For guidance, download the 'Figure Guide PDF': (https://www.embopress.org/page/journal/17574684/authorguide#figureformat).

3) A .docx formatted letter INCLUDING the reviewers' reports and your detailed point-by-point responses to their comments. As part of the EMBO Press transparent editorial process, the point-by-point response is part of the Review Process File (RPF), which will be published alongside your paper.

4) A complete author checklist, which you can download from our author guidelines (https://www.embopress.org/page/journal/17574684/authorguide#submissionofrevisions). Please insert information in the checklist that is also reflected in the manuscript. The completed author checklist will also be part of the RPF.

5) Please note that all corresponding authors are required to supply an ORCID ID for their name upon submission of a revised manuscript.

6) It is mandatory to include a 'Data Availability' section after the Materials and Methods. Before submitting your revision, primary datasets produced in this study need to be deposited in an appropriate public database, and the accession numbers and

database listed under 'Data Availability'. Please remember to provide a reviewer password if the datasets are not yet public (see https://www.embopress.org/page/journal/17574684/authorguide#dataavailability).

In case you have no data that requires deposition in a public database, please state so in this section. Note that the Data Availability Section is restricted to new primary data that are part of this study.

7) For data quantification: please specify the name of the statistical test used to generate error bars and P values, the number (n) of independent experiments (specify technical or biological replicates) underlying each data point and the test used to calculate p-values in each figure legend. The figure legends should contain a basic description of n, P and the test applied. Graphs must include a description of the bars and the error bars (s.d., s.e.m.). See also 'Figure Legend' guidelines: https://www.embopress.org/page/journal/17574684/authorguide#figureformat

8) At EMBO Press we ask authors to provide source data for the main manuscript figures. Our source data coordinator will contact you to discuss which figure panels we would need source data for and will also provide you with helpful tips on how to upload and organize the files.

9) Our journal encourages inclusion of *data citations in the reference list* to directly cite datasets that were re-used and obtained from public databases. Data citations in the article text are distinct from normal bibliographical citations and should directly link to the database records from which the data can be accessed. In the main text, data citations are formatted as follows: "Data ref: Smith et al, 2001" or "Data ref: NCBI Sequence Read Archive PRJNA342805, 2017". In the Reference list, data citations must be labeled with "[DATASET]". A data reference must provide the database name, accession number/identifiers and a resolvable link to the landing page from which the data can be accessed at the end of the reference. Further instructions are available at .

10) We replaced Supplementary Information with Expanded View (EV) Figures and Tables that are collapsible/expandable online. A maximum of 5 EV Figures can be typeset. EV Figures should be cited as 'Figure EV1, Figure EV2" etc... in the text and their respective legends should be included in the main text after the legends of regular figures.

- For the figures that you do NOT wish to display as Expanded View figures, they should be bundled together with their legends in a single PDF file called *Appendix*, which should start with a short Table of Content. Appendix figures should be referred to in the main text as: "Appendix Figure S1, Appendix Figure S2" etc.

- Additional Tables/Datasets should be labeled and referred to as Table EV1, Dataset EV1, etc. Legends have to be provided in a separate tab in case of .xls files. Alternatively, the legend can be supplied as a separate text file (README) and zipped together with the Table/Dataset file.

See detailed instructions here:

.

11) The paper explained: EMBO Molecular Medicine articles are accompanied by a summary of the articles to emphasize the major findings in the paper and their medical implications for the non-specialist reader. Please provide a draft summary of your article highlighting

- the medical issue you are addressing,

- the results obtained and

- their clinical impact.

This may be edited to ensure that readers understand the significance and context of the research. Please refer to any of our published articles for an example.

12) For more information: There is space at the end of each article to list relevant web links for further consultation by our readers. Could you identify some relevant ones and provide such information as well? Some examples are patient associations, relevant databases, OMIM/proteins/genes links, author's websites, etc...

13) Author contributions: You will be asked to provide CRediT (Contributor Role Taxonomy) terms in the submission system. These replace a narrative author contribution section in the manuscript.

14) A Conflict of Interest statement should be provided in the main text.

15) Every published paper now includes a 'Synopsis' to further enhance discoverability. Synopses are displayed on the journal

webpage and are freely accessible to all readers. They include a short stand first (maximum of 300 characters, including space) as well as 2-5 one-sentences bullet points that summarizes the paper. Please write the bullet points to summarize the key NEW findings. They should be designed to be complementary to the abstract - i.e. not repeat the same text. We encourage inclusion of key acronyms and quantitative information (maximum of 30 words / bullet point). Please use the passive voice. Please attach these in a separate file or send them by email, we will incorporate them accordingly.

Please also suggest a striking image or visual abstract to illustrate your article as a PNG file 550 px wide x 300-600 px high.

EMBO Molecular Medicine has a "scooping protection" policy, whereby similar findings that are published by others during review or revision are not a criterion for rejection. Should you decide to submit a revised version, I do ask that you get in touch after three months if you have not completed it, to update us on the status.

Please note: When submitting your revision you will be prompted to enter your funding and payment information. This will allow Wiley to send you a quote for the article processing charge (APC) in case of acceptance. This quote takes into account any reduction or fee waivers that you may be eligible for. Authors do not need to pay any fees before their manuscript is accepted and transferred to the publisher.

EMBO Press participates in many Publish and Read agreements that allow authors to publish Open Access with reduced/no publication charges. Check your eligibility: https://authorservices.wiley.com/author-resources/Journal-Authors/open-access/affiliation-policies-payments/index.html

***** Reviewer's comments *****

Referee #1 (Remarks for Author):

Rodriquez-Ruiz and colleagues present data regarding NLRP1 activation in zebrafish, related to LRRFIP1/FLII and ZAKa, with relevance for the system in humans. Overall there are a lot of separate parts to this investigation, and in many places additional controls could be warranted. To some extent, the LRRFIP1/FLII and ZAKa parts seem separable, but with some additional in vitro analysis may link together more clearly.

Major points

The epistasis experiment showing enforced FLII does not reduce neutrophil numbers from LRRFIP1 deficient fish is very interesting. Previously FLII was reported to inhibit Caspase-1 (PMID: 18411310), can the authors determine why that does not occur in this context? Is BCAP involved or dispensable for the effect of FLII on NLRP1 in zebrafish?

It seems somewhat surprising that agents triggering ribotoxic stress such as ansiomycin had effects consistent with NLRP1 activation in vivo when used systemically. In many cell types, this type of insult results in apoptosis, as opposed to pyroptosis, and it may have been expected to be toxic when used in vivo at concentrations that activate NLRP1.

The experiments with overexpressed NLRP1 (S107D/A) appear straightforward, however the interpretation seems challenging and somewhat indirect. As the conclusion appears to be that LRRFIP1/FLII inhibit NLRP1 by preventing phosphorylation of S107, perhaps it would be best to try and document this formally with biochemical analysis in vitro?

Referee #2 (Comments on Novelty/Model System for Author):

See my review.
Technical quality - overall it is high, but although there is biological replication within data presented (for example, there are multiple embryos), there is paucity of information about experimental replication (whether the comparison or intervention was performed more than once). This is one of major points to the authors.
Novelty - to my knowledge, the pathway assembled is new
Medical impact - there is potential medical impact. The implication for ribosomal-stress diseases is based on a single cell line + chemical treatment model. The implication for inflammatory diseases is based on an in vivo model. A repurposed drug is evaluated.
Adequacy of the model system - the use of a human cell line and zebrafish models is a strength of the paper. It has provided opportunity for biochemical studies particularly in the human cell line system, and correlative genetic studies in both systems.
Re (5) clarity and Interest for the nonspecialist - the genes involved have impossible non-intuitive names and abbreviations that provide little help for the non-specialist to understand the pathway being assembled; I have suggested a pathway diagram among the main figures (most likely it will be the last figure).

Referee #2 (Remarks for Author):

The manuscript of Rodriguez-Ruiz et al presents a comprehensive set of experiments that implicate the NLRP1 inflammasome in influencing the erythroid/myeloid balance of haematopoietic output from HSCs. Molecular mechanistic studies link LRRFIP1 and FLII, and the ZAKalpha/P38 kinase pathway.

Overall the manuscript is comprehensive, logical and well-presented. The scientific logic and the evidence for the genetic and molecular mechanisms are thorough, of high technical quality, and supported by the evidence presented.

Major comments

1. The gene editing efficacy analysis shows that the crRNAs designed for either lrrfip1a and lrrfip1b editing each actually directed editing of both lrrfip1a and lrrfip1b at approx. equal efficiencies, but overall to a different degree (20% and 20%, and 65% and 85% respectively). While the text correctly acknowledges this by referring to the larvae as deficient in both paralogs (as in lines 168-169), the x-axis labelling within the figures (e.g. as in Figure 4) uses crRNA design intent rather than the actual edited outcome. This is somewhat misleading, and will be so especially to readers who may survey the figures and not read the very fine detail of the narrative text. The author's already provide a solution for this in Fig 4F. Following that precedent, these two crRNAs would be better labelled "crRNA irrfip1a/b-1" and "crRNA irrfip1a/b-2" to reflect what they did, rather than what they were designed to do, throughout the entire paper.

2. Throughout the paper, it not clear what degree of replication was behind the experiments. While the plots indicate the number of zebrafish embryos evaluated, it is never clear how many times an experiment was independently performed from start to finish. For example, in Fig 4 (all panels) were the groups in one panel from a single parallel study? Were any panels compiled as composites from non-parallel experiments? Did all the embryos represented in one outcome result from a single run or from multiple runs of the experiment? In panel H, how many independent times was the drug treatment applied and this outcome observed? For such an experiment, the usual expectation would be n=3 independent replicates, independent of the total number of individual embryos in each iteration of the assay. Similarly, the replication of all biochemical assays such as Western blots, IPs etc is not clear. In Fig 3, the variation appears to be from technical replicates within one run of an in vitro experiment.

3. The testing of interventions based on these new mechanistic insights to intentionally alter hematopoietic outcomes in K562 cells and the zebrafish spint1a mutant model is a particularly interesting aspect of this report. This certainly adds to the significance of the report, and in the discussion the consideration if this is appropriately qualified. While these data support the claim of the final abstract sentence (lines 44-47) that they "reveal" "novel therapeutic strategies", this seemed too strong. That the insights from this work might help rare ribosomal-stress diseases (such as Diamond-Blackfan anemia) is hypothesised, suggested, or a possibility for the future - suggest the use of more qualified language.

4. I suggest that many readers' understanding of the mechanism in paper would be greatly assisted by having the overall molecular mechanism summarised in a figure within the main paper, rather than just in supplementary material.

Minor comments

1. Abstract (line 44) conclusion (NOT conclusions)

2. Line 111. From the methods sections and all the data presented, it appears that mfap4-driven reporter lines were used for studies of macrophage lineage cells, not mpeg1-driven reporter lines as stated here.

3. Figure S1B - there are 46 genes listed, not 50 as stated in the legend.

4. Almost all figures and supplementary figures with photomicrographs. Scale bars are labelled "uM" (which means micro-moles/liter) instead of "um" for micrometres (for example, it is incorrect in Fig 1D; it is correct in Figure 3B-C). In figures such as Fig 1D, it is confusing, because there is also the concentration of Hemin to consider, which was 50 microM.

5. Nomenclature. It is acknowledged that in a paper that uses a human cell line and zebrafish in vivo models it is very complex to apply an absolutely precise gene/RNA/protein capitalisation/upper-lower case punctuation nomenclature convention - some generic text applying to both species cannot be avoided. However, there is still a need for greater consistency in some places. Lines 158-175 describing the generation of the gene edited lines correctly introduce the zebrafish gene names as lower-case italicised (line 161). The multiple mentions of the target in lines 163-165 are target genes, and should be italics. The resultant lines as "Lrrfip1a-deficient" etc, a nomenclature convention indicating that they are deficient in the protein, although this has not been experimentally demonstrated (there is precise attention to what occurred at the genomic (DNA) and transcriptomic (RNA) levels, but the not protein level). Make this consistent here, and check these nomenclature conventions throughout the manuscript.

6. There are also inconsistencies though the figure labels whether the reporter line is (Lyz:dsRED) or (lyz:dsRED), (Mfap4:tomato) or (mfap4:tomato) - lower case is correct.

7. Tables S1 and S2 - despite specifically referring to use of recommended nomenclature guidelines, several gene names are inconsistently formatted between the two tables.

8. Fig7K, Fig 8H y-axis - adding "CASP1" to the label would be helpful (as in Fig 1I).

9. Figure S1A - hybridisation (NOT hibridation). Whether "labelling" and "labelled" should have -l- or -ll- is confusing, but use either one or the other consistently, not both.

10. Fig S12G. Graphics incomplete.

11. Line 855. Should be Figure S2 (NOT Figure 2).

12. Fig 3B. Bold Hemin and Anysomicine labels for consistency.

13. Line 265 "fantastic" - this is an informal use of "fantastic" to mean "very very good" - "uniquely appropriate" or similar would be better.

Referee #1 (Remarks for Author):

Rodriquez-Ruiz and colleagues present data regarding NLRP1 activation in zebrafish, related to LRRFIP1/FLII and ZAKa, with relevance for the system in humans. Overall there are a lot of separate parts to this investigation, and in many places additional controls could be warranted. To some extent, the LRRFIP1/FLII and ZAKa parts seem separable, but with some additional in vitro analysis may link together more clearly.

Major points

The epistasis experiment showing enforced FLII does not reduce neutrophil numbers from LRRFIP1 deficient fish is very interesting. Previously FLII was reported to inhibit Caspase-1 (PMID: 18411310), can the authors determine why that does not occur in this context? Is BCAP involved or dispensable for the effect of FLII on NLRP1 in zebrafish?
This is an interesting point. As we discussed in the manuscript, LRRFIP2 directly interact and inhibit the activation of macrophage NLRP3 inflammasome by recruiting FLII and facilitating the inhibition of caspase-1. However, LRRFIP1 is unable to interact with NLRP3. However, our biochemical and functional results show that a similar mechanism operates to inhibit the NLRP1 inflammasome but mediated by LRRFIP1.

Although it would be interesting to address whether BCAP is required for the negative regulation of NLRP1 inflammasome by LRRFIP1/FLII in future studies, we did not find BCAP in our MS analysis. However, we find novel proteins and recently reported to regulate NLRP1 inflammasome activity, such as thioredoxin (PMID: 36332009). However, we have discussed these points in the revised manuscript (lines 322-324 and 329-333).

It seems somewhat surprising that agents triggering ribotoxic stress such as ansiomycin had effects consistent with NLRP1 activation in vivo when used systemically. In many cell types, this type of insult results in apoptosis, as opposed to pyroptosis, and it may have been expected to be toxic when used in vivo at concentrations that activate NLRP1.
We agree on this point. The concentrations used of anisomycin activates Nlrp1 inflammasome in zebrafish larvae and alters hematopoiesis. However, we did not find any toxicity or effect on development. This suggests that erythropoiesis is highly affected by translation efficiency of ribosomes, and this is consistent with ribosomopathies, such as DBA, where *GATA1* translation is impaired.

The experiments with overexpressed NLRP1 (S107D/A) appear straightforward, however the interpretation seems challenging and somewhat indirect. As the conclusion appears to be that LRRFIP1/FLII inhibit NLRP1 by preventing phosphorylation of S107, perhaps it would be best to try and document this formally with biochemical analysis in vitro?
Our functional studies in zebrafish demonstrate that the phosphorylation of human NLRP1 at S107 by the Zaka/P38 kinase axis is essential to regulate hematopoiesis and that the mechanism is conserved between zebrafish and human. We did not conclude

that LRRFIP1/FLII regulate NLRP1 activation by preventing phosphorylation of S107.It is presumably that LRRFIP1/FLII inhibit caspase-1 downstream NLRP1 inflammasome activation, since FLII is a caspase-1 pseudosubstrate, although other mechanisms may also operate since LRRFIP1 and FLII block NLRP1-induced ASC speck formation in HEK293 cells. Anyway, demonstrating whether LRRFIP1/FLII inhibit the phosphorylation of NLRP1 is technically challenging, as it would require the production of recombinant NLRP1, P38, LRRFIP1 and FLII for performing radiometric in vitro kinase assays (PMID: 36315050). Therefore, we think this study requires a great effort and the proposed mechanism is not supported by experimental evidence. However, we have discussed this point in the revised manuscript (lines 333-337).

Referee #2 (Remarks for Author):

The manuscript of Rodriguez-Ruiz et al presents a comprehensive set of experiments that implicate the NLRP1 inflammasome in influencing the erythroid/myeloid balance of haematopoietic output from HSCs. Molecular mechanistic studies link LRRFIP1 and FLII, and the ZAKalpha/P38 kinase pathway.

Overall the manuscript is comprehensive, logical and well-presented. The scientific logic and the evidence for the genetic and molecular mechanisms are thorough, of high technical quality, and supported by the evidence presented.
We are pleased with the reviewer's comments on our manuscript.

Major comments

1. The gene editing efficacy analysis shows that the crRNAs designed for either lrrfip1a and lrrfip1b editing each actually directed editing of both lrrfip1a and lrrfip1b at approx. equal efficiencies, but overall to a different degree (20% and 20%, and 65% and 85% respectively). While the text correctly acknowledges this by referring to the larvae as deficient in both paralogs (as in lines 168-169), the x-axis labelling within the figures (e.g. as in Figure 4) uses crRNA design intent rather than the actual edited outcome. This is somewhat misleading, and will be so especially to readers who may survey the figures and not read the very fine detail of the narrative text. The author's already provide a solution for this in Fig 4F. Following that precedent, these two crRNAs would be better labelled "crRNA irrfip1a/b-1" and "crRNA irrfip1a/b-2" to reflect what they did, rather than what they were designed to do, throughout the entire paper.
Thanks for this observation. In Figs 4F and S14I, we used both gRNAs together. Therefore, to clarify this issue we have include a note in the legends of the affected figures which reads: "Note that either *lrrfip1a* or *lrrfip1b* crRNAs/Cas9 complexes target both *lrrfip1* paralogs (see Appendix Figure S10).".

2. Throughout the paper, it not clear what degree of replication was behind the experiments. While the plots indicate the number of zebrafish embryos evaluated, it is never clear how many times an experiment was independently performed from start to finish. For example, in Fig 4 (all panels) were the groups in one panel from a single parallel study? Were any panels compiled as composites from non-parallel experiments? Did all the embryos represented in one outcome result from a single run or from multiple runs of the experiment? In panel H, how many independent times was the drug treatment applied and this outcome observed? For such an experiment, the usual expectation would be n=3 independent replicates, independent of the total number of individual embryos in each iteration of the assay. Similarly, the replication of all biochemical assays such as Western blots, IPs etc is not clear. In Fig 3, the variation appears to be from technical replicates within one run of an in vitro experiment.
We have clarified this in the M&M section which now reads: " At least 3 independent experiments were performed with zebrafish larvae and biochemical studies. Three independent caspase-1 activity assays were performed in all experiments using a pool of 30 larvae and one representative experiment is shown with 3 technical replicates. All larvae from the different independent experiments were pooled for plotting and statistical analysis. The number of total larvae analyzed in each experiment is indicated in all figures". Therefore, Fig 4H shows a pool of larvae from 3 independent

experiments and Fig 3 shows the quantification of specks from 3 independent experiments.

3. The testing of interventions based on these new mechanistic insights to intentionally alter hematopoietic outcomes in K562 cells and the zebrafish spint1a mutant model is a particularly interesting aspect of this report. This certainly adds to the significance of the report, and in the discussion the consideration if this is appropriately qualified. While these data support the claim of the final abstract sentence (lines 44-47) that they "reveal" "novel therapeutic strategies", this seemed too strong. That the insights from this work might help rare ribosomal-stress diseases (such as Diamond-Blackfan anemia) is hypothesised, suggested, or a possibility for the future - suggest the use of more qualified language.
We have rewritten this sentence to dampen our conclusion. It now reads: "In conclusion, our results reveal that the NLRP1 inflammasome regulates hematopoiesis and pave the way to develop novel therapeutic strategies for the treatment of hematopoietic alterations associated with chronic inflammatory and rare diseases."

4. I suggest that many readers' understanding of the mechanism in paper would be greatly assisted by having the overall molecular mechanism summarised in a figure within the main paper, rather than just in supplementary material.
Thanks for this suggestion. We have moved Fig S19 to the main figures and it is now Fig. 9.

Minor comments

1. Abstract (line 44) conclusion (NOT conclusions)
Corrected.

2. Line 111. From the methods sections and all the data presented, it appears that mfap4-driven reporter lines were used for studies of macrophage lineage cells, not mpeg1-driven reporter lines as stated here.
You are right; we used mfap4 line in this paper. It has been corrected.

3. Figure S1B - there are 46 genes listed, not 50 as stated in the legend.
Corrected.

4. Almost all figures and supplementary figures with photomicrographs. Scale bars are labelled "uM" (which means micro-moles/liter) instead of "um" for micrometres (for example, it is incorrect in Fig 1D; it is correct in Figure 3B-C). In figures such as Fig 1D, it is confusing, because there is also the concentration of Hemin to consider, which was 50 microM.
We have corrected them.

5. Nomenclature. It is acknowledged that in a paper that uses a human cell line and zebrafish in vivo models it is very complex to apply an absolutely precise gene/RNA/protein capitalisation/upper-lower case punctuation nomenclature convention - some generic text applying to both species cannot be avoided. However, there is still a need for greater consistency in some places. Lines 158-175 describing the generation of the gene edited lines correctly introduce the zebrafish gene names as lower-case italicised (line 161). The multiple mentions of the target in lines 163-165 are target genes, and should be italics. The resultant lines as "Lrrfip1a-deficient" etc, a nomenclature convention indicating that they are deficient in the protein, although this has not been experimentally demonstrated (there is precise attention to what occurred at the genomic (DNA) and transcriptomic (RNA) levels, but the not protein level). Make this consistent here, and check these nomenclature conventions

throughout the manuscript.
We have double checked and corrected them. We have also replaced deficient by crispant and use gene moneclature, for example "*nlrp1* crispant larvae".

6. There are also inconsistencies though the figure labels whether the reporter line is (Lyz:dsRED) or (lyz:dsRED), (Mfap4:tomato) or (mfap4:tomato) - lower case is correct.
We have corrected them.

7. Tables S1 and S2 - despite specifically referring to use of recommended nomenclature guidelines, several gene names are inconsistently formatted between the two tables.
In Table S1, primers for both human and zebrafish are shown. We have now clarified this.

8. Fig7K, Fig 8H y-axis - adding "CASP1" to the label would be helpful (as in Fig 1I).
Corrected.

9. Figure S1A - hybridisation (NOT hibridation). Whether "labelling" and "labelled" should have -l- or -ll- is confusing, but use either one or the other consistently, not both.
Corrected.

10. Fig S12G. Graphics incomplete.
Corrected.

11. Line 855. Should be Figure S2 (NOT Figure 2).
Corrected.

12. Fig 3B. Bold Hemin and Anysomicine labels for consistency.
Changed.

13. Line 265 "fantastic" - this is an informal use of "fantastic" to mean "very very good" - "uniquely appropriate" or similar would be better.

Replaced as suggested.

15th Aug 2023

Dear Prof. Mulero,

Thank you for the submission of your revised manuscript to EMBO Molecular Medicine. I am pleased to inform you that we will be able to accept your manuscript pending the following final amendments:

1) Please address all the minor points raised by the referees.
2) In the main manuscript file, please do the following:
- Correct/answer the track changes suggested by our data editors by working from the attached document.
- Limit keywords to max. 5.
- Remove track changes.
- Please add callouts for Fig.6B and Fig. 9A-B.
- Please rename "Disclosure Statement" to "Disclosure Statement & Competing Interests". We updated our journal's competing interests policy in January 2022 and request authors to consider both actual and perceived competing interests. Please review the policy https://www.embopress.org/competing-interests and update your competing interests if necessary.
- Correct the reference citation in the reference list. Where there are more than 10 authors on a paper, 10 will be listed, followed by "et al.". Please check "Author Guidelines" for more information.
https://www.embopress.org/page/journal/17574684/authorguide#referencesformat
3) Funding: Please merge it with "Acknowledgments" and make sure that information about all sources of funding are complete in both our submission system and in the manuscript. Currently MCIN/AEI/10.13039/501100011033, Juan de la Cierva-Incorporación postdoctoral contract, PhD fellowship to LR-R, ISCIII (Miguel Servet CP20/00028 and CP21/00028, Consejería de Salud de la CARM are missing in our submission system.
4) Synopsis:
- Synopsis image: Please provide the visual abstract as a high-resolution jpeg file 550 px-wide x (250-400)-px high.
- Please check your synopsis text and image before submission with your revised manuscript. Please be aware that in the proof stage minor corrections only are allowed (e.g., typos).
5) For more information: This space should be used to list relevant web links for further consultation by our readers. Could you identify some relevant ones and provide such information as well? Some examples are patient associations, relevant databases, OMIM/proteins/genes links, author's websites, etc...
6) Source data: Please fill out the source data checklist and indicate all source data that are deposited in a public repository in designated field. Upload all SD for one figure as one zipped file per figure.
7) As part of the EMBO Publications transparent editorial process initiative (see our Editorial at http://embomolmed.embopress.org/content/2/9/329), EMBO Molecular Medicine will publish online a Review Process File (RPF) to accompany accepted manuscripts. This file will be published in conjunction with your paper and will include the anonymous referee reports, your point-by-point response and all pertinent correspondence relating to the manuscript. Let us know whether you agree with the publication of the RPF and as here, if you want to remove or not any figures from it prior to publication. Please note that the Authors checklist will be published at the end of the RPF.
8) Please provide a point-by-point letter INCLUDING my comments as well as the reviewer's reports and your detailed responses (as Word file).

I look forward to reading a new revised version of your manuscript as soon as possible.

Yours sincerely,

Zeljko Durdevic

Zeljko Durdevic
Editor
EMBO Molecular Medicine

*** Instructions to submit your revised manuscript ***

*** PLEASE NOTE *** As part of the EMBO Publications transparent editorial process initiative (see our Editorial at https://www.embopress.org/doi/pdf/10.1002/emmm.201000094), EMBO Molecular Medicine will publish online a Review Process File to accompany accepted manuscripts.

In the event of acceptance, this file will be published in conjunction with your paper and will include the anonymous referee reports, your point-by-point response and all pertinent correspondence relating to the manuscript. If you do NOT want this file to be published, please inform the editorial office at contact@embomolmed.org.

When submitting your revised manuscript, please include:

1) a .docx formatted version of the manuscript text (including Figure legends and tables)

2) Separate figure files*

3) supplemental information as Expanded View and/or Appendix. Please carefully check the authors guidelines for formatting Expanded view and Appendix figures and tables at https://www.embopress.org/page/journal/17574684/authorguide#expandedview

4) a letter INCLUDING the reviewer's reports and your detailed responses to their comments (as Word file).

5) The paper explained: EMBO Molecular Medicine articles are accompanied by a summary of the articles to emphasize the major findings in the paper and their medical implications for the non-specialist reader. Please provide a draft summary of your article highlighting
- the medical issue you are addressing,
- the results obtained and
- their clinical impact.
This may be edited to ensure that readers understand the significance and context of the research.
Please refer to any of our published articles for an example.

6) For more information: There is space at the end of each article to list relevant web links for further consultation by our readers. Could you identify some relevant ones and provide such information as well? Some examples are patient associations, relevant databases, OMIM/proteins/genes links, author's websites, etc...

7) Author contributions: the contribution of every author must be detailed in a separate section.

8) EMBO Molecular Medicine now requires a complete author checklist (https://www.embopress.org/page/journal/17574684/authorguide) to be submitted with all revised manuscripts. Please use the checklist as guideline for the sort of information we need WITHIN the manuscript. The checklist should only be filled with page numbers were the information can be found. This is particularly important for animal reporting, antibody dilutions (missing) and exact values and n that should be indicted instead of a range.

9) Every published paper now includes a 'Synopsis' to further enhance discoverability. Synopses are displayed on the journal webpage and are freely accessible to all readers. They include a short stand first (maximum of 300 characters, including space) as well as 2-5 one sentence bullet points that summarise the paper. Please write the bullet points to summarise the key NEW findings. They should be designed to be complementary to the abstract - i.e. not repeat the same text. We encourage inclusion of key acronyms and quantitative information (maximum of 30 words / bullet point). Please use the passive voice. Please attach these in a separate file or send them by email, we will incorporate them accordingly.

You are also welcome to suggest a striking image or visual abstract to illustrate your article. If you do please provide a jpeg file 550 px-wide x 400-px high.

10) A Conflict of Interest statement should be provided in the main text

11) Please note that we now mandate that all corresponding authors list an ORCID digital identifier. This takes <90 seconds to complete. We encourage all authors to supply an ORCID identifier, which will be linked to their name for unambiguous name identification.

Currently, our records indicate that the ORCID for your account is 0000-0001-9527-0211.

Please click the link below to modify this ORCID:
Link Not Available

12) The system will prompt you to fill in your funding and payment information. This will allow Wiley to send you a quote for the article processing charge (APC) in case of acceptance. This quote takes into account any reduction or fee waivers that you may be eligible for. Authors do not need to pay any fees before their manuscript is accepted and transferred to our publisher.

*Additional important information regarding Figures

Each figure should be given in a separate file and should have the following resolution:
Graphs 800-1,200 DPI
Photos 400-800 DPI
Colour (only CMYK) 300-400 DPI"

Figures are not edited by the production team. All lettering should be the same size and style; figure panels should be indicated by capital letters (A, B, C etc). Gridlines are not allowed except for log plots. Figures should be numbered in the order of their appearance in the text with Arabic numerals. Each Figure must have a separate legend and a caption is needed for each panel.

*Additional important information regarding figures and illustrations can be found at https://bit.ly/EMBOPressFigurePreparationGuideline. See also figure legend preparation guidelines: https://www.embopress.org/page/journal/17574684/authorguide#figureformat

The system will prompt you to fill in your funding and payment information. This will allow Wiley to send you a quote for the article processing charge (APC) in case of acceptance. This quote takes into account any reduction or fee waivers that you may be eligible for. Authors do not need to pay any fees before their manuscript is accepted and transferred to our publisher.

***** Reviewer's comments *****

Referee #1 (Comments on Novelty/Model System for Author):

Rodriquez-Ruiz and colleagues present data regarding NLRP1 activation in zebrafish, related to LRRFIP1/FLII and ZAKa, with relevance for the system in humans. Overall there are a lot of separate parts to this investigation, and I remain confused about the LRRFIP1/FLII data.

Referee #1 (Remarks for Author):

Two of my major points from the previous revisions remain only partially addressed.

1. Why enforced FLII did not reduce neutrophil numbers from LRRFIP1 deficient fish, there was no response.

3. Given that LRRFIP/FLII might inhibit either NLRP1/ASC spec formation, or Caspase-1 activity, some experiment that could distinguish between them in this context seems important. At least NLRP1 phosphorylation could be assessed in cell lysates using phos-tag gels or MS, which would not require radiometric in vitro kinase assays and helps link these parts of the manuscript together?

Referee #2 (Comments on Novelty/Model System for Author):

Technical quality has been improved in the review process.

Referee #2 (Remarks for Author):

The authors have addressed my previous major and minor comments satisfactorily, and I have noted the responses to the other reviewer's comments.

Congratulations to the authors for their interesting and well-presented work.

Remaining errors:
The authors clarified that only the Tg(mfap4:mCherry)line was used; the ump6 allele is listed in line 375. For this reason:
Figure 7, Panel I. Labelled Tg(mpeg:mCherry). I believe this should be Tg(mfap4:mCherry).
Figure S13, Panel C. Labelled Tg(mfap4:tomato). I believe this should be Tg(mfap4:mCherry).
Figure S14, Panel E. Tg(mfap4:mcherry). For consistency should be Tg(mfap4:mCherry) i.e. uppercase "C".

EDITOR

1) Please address all the minor points raised by the referees.

Please, see below.

2) In the main manuscript file, please do the following:
- Correct/answer the track changes suggested by our data editors by working from the attached document.
- Limit keywords to max. 5.
- Remove track changes.
- Please add callouts for Fig.6B and Fig. 9A-B.
- Please rename "Disclosure Statement" to "Disclosure Statement & Competing Interests". We updated our journal's competing interests policy in January 2022 and request authors to consider both actual and perceived competing interests. Please review the policy https://www.embopress.org/competing-interests and update your competing interests if necessary.
- Correct the reference citation in the reference list. Where there are more than 10 authors on a paper, 10 will be listed, followed by "et al.". Please check "Author Guidelines" for more information. https://www.embopress.org/page/journal/17574684/authorguide#referencesformat

All changes done. The callout for Figure 9 is in the Discussion section.

3) Funding: Please merge it with "Acknowledgments" and make sure that information about all sources of funding are complete in both our submission system and in the manuscript. Currently MCIN/AEI/10.13039/501100011033, Juan de la Cierva-Incorporación postdoctoral contract, PhD fellowship to LR-R, ISCIII (Miguel Servet CP20/00028 and CP21/00028, Consejería de Salud de la CARM are missing in our submission system.

Done.

4) Synopsis:
- Synopsis image: Please provide the visual abstract as a high-resolution jpeg file 550 px-wide x (250-400)-px high.
- Please check your synopsis text and image before submission with your revised manuscript. Please be aware that in the proof stage minor corrections only are allowed (e.g., typos).

Done. We have included the same synopsis image files: one following your recommendations and another one with better resolution. So, you can choose the one that better fit your guidelines.

5) For more information: This space should be used to list relevant web links for further consultation by our readers. Could you identify some relevant ones and provide such information as well? Some examples are patient associations, relevant databases, OMIM/proteins/genes links, author's websites, etc...

We think the following web links are relevant for our paper:

1. OMIM for Diamond-Blackfan anemia: https://www.omim.org/entry/105650

2. Diamond-Blackfan anemia Foundation: https://dbafoundation.org/

6) Source data: Please fill out the source data checklist and indicate all source data that are deposited in a public repository in designated field. Upload all SD for one figure as one zipped file per figure.
I am sorry that I forgot to upload it with my previous revision.

7) As part of the EMBO Publications transparent editorial process initiative (see our Editorial at http://embomolmed.embopress.org/content/2/9/329), EMBO Molecular Medicine will publish online a Review Process File (RPF) to accompany accepted manuscripts. This file will be published in conjunction with your paper and will include the anonymous referee reports, your point-by-point response and all pertinent correspondence relating to the manuscript. Let us know whether you agree with the publication of the RPF and as here, if you want to remove or not any figures from it prior to publication. Please note that the Authors checklist will be published at the end of the RPF.

Agree.

Referee #1 (Remarks for Author):

Two of my major points from the previous revisions remain only partially addressed.

1. Why enforced FLII did not reduce neutrophil numbers from LRRFIP1 deficient fish, there was no response.

As we have indicated in our manuscript, these epistasis studies suggest that the ability of Flii to inhibit the Nlrp1 inflammasome is dependent on Lrrfip1 (lines 187-188). WE have now also discussed this point in the revised version (lines 321-324).

3. Given that LRRFIP/FLII might inhibit either NLRP1/ASC spec formation, or Caspase-1 activity, some experiment that could distinguish between them in this context seems important. At least NLRP1 phosphorylation could be assessed in cell lysates using phos-tag gels or MS, which would not require radiometric in vitro kinase assays and helps link these parts of the manuscript together?

We have performed this experiment with phos-tag and, unfortunately, were unable to detect NLRP1 phosphorylation.

Referee #2 (Comments on Novelty/Model System for Author):

Technical quality has been improved in the review process.

Referee #2 (Remarks for Author):

The authors have addressed my previous major and minor comments satisfactorily, and I have noted the responses to the other reviewer's comments.

Congratulations to the authors for their interesting and well-presented work.

We thanks that our responses satisfy this reviewer.

Remaining errors:

The authors clarified that only the Tg(mfap4:mCherry)line was used; the ump6 allele is listed in line 375. For this reason:

Figure 7, Panel I. Labelled Tg(mpeg:mCherry). I believe this should be Tg(mfap4:mCherry).

Figure S13, Panel C. Labelled Tg(mfap4:tomato). I believe this should be Tg(mfap4:mCherry).

Figure S14, Panel E. Tg(mfap4:mcherry). For consistency should be Tg(mfap4:mCherry) i.e. uppercase "C".

We are sorry for this mistake. We have used the line Tg(mfap4.1:Tomato)^{xt12}. We have corrected all figures and the manuscript.

22nd Aug 2023

Dear Prof. Mulero,

We are pleased to inform you that your manuscript is accepted for publication and is now being sent to our publisher to be included in the next available issue of EMBO Molecular Medicine.

Of note, deposited microscopy images at BioStudies repository under the accession number S-BIAD823 are currently not accessible. Please be aware that all deposited data should be freely available upon publication.

Please read below for additional IMPORTANT information regarding your article, its publication and the production process.

Congratulations on your interesting work,

Zeljko Durdevic

Zeljko Durdevic
Editor
EMBO Molecular Medicine

Follow us on Twitter @EmboMolMed
Sign up for eTOCs at embopress.org/alertsfeeds

*** *** *** IMPORTANT INFORMATION *** *** ***

SPEED OF PUBLICATION
The journal aims for rapid publication of papers, using using the advance online publication "Early View" to expedite the process: A properly copy-edited and formatted version will be published as "Early View" after the proofs have been corrected. Please help the Editors and publisher avoid delays by providing e-mail address(es), telephone and fax numbers at which author(s) can be contacted.

Should you be planning a Press Release on your article, please get in contact with embomolmed@wiley.com as early as possible, in order to coordinate publication and release dates.

LICENSE AND PAYMENT:

All articles published in EMBO Molecular Medicine are fully open access: immediately and freely available to read, download and share.

EMBO Molecular Medicine charges an article processing charge (APC) to cover the publication costs. You, as the corresponding author for this manuscript, should have already received a quote with the article processing fee separately. Please let us know in case this quote has not been received.

Once your article is at Wiley for editorial production you will receive an email from Wiley's Author Services system, which will ask you to log in and will present you with the publication license form for completion. Within the same system the publication fee can be paid by credit card, an invoice, pro forma invoice or purchase order can be requested.

Payment of the publication charge and the signed Open Access Agreement form must be received before the article can be published online.

PROOFS

You will receive the proofs by e-mail approximately 2 weeks after all relevant files have been sent o our Production Office. Please return them within 48 hours and if there should be any problems, please contact the production office at

embopressproduction@wiley.com.

Please inform us if there is likely to be any difficulty in reaching you at the above address at that time. Failure to meet our deadlines may result in a delay of publication.

All further communications concerning your paper proofs should quote reference number EMM-2023-18142-V3 and be directed to the production office at embopressproduction@wiley.com.

Thank you,

Zeljko Durdevic
Editor
EMBO Molecular Medicine

EMBO Press Author Checklist

Corresponding Author Name: Victoriano Mulero	
Journal Submitted to: EMBO Molecular Medicine	
Manuscript Number: EMM-2023-18142	

USEFUL LINKS FOR COMPLETING THIS FORM
The EMBO Journal - Author Guidelines
EMBO Reports - Author Guidelines
Molecular Systems Biology - Author Guidelines
EMBO Molecular Medicine - Author Guidelines

Reporting Checklist for Life Science Articles (updated January
This checklist is adapted from Materials Design Analysis Reporting (MDAR) Checklist for Authors. MDAR establishes a minimum set of requirements in transparent reporting in the life sciences (see Statement of Task: 10.31222/osf.io/9sm4x). Please follow the journal's guidelines in preparing your manuscript.
Please note that a copy of this checklist will be published alongside your article.

Abridged guidelines for figures
1. Data
The data shown in figures should satisfy the following conditions:
- the data were obtained and processed according to the field's best practice and are presented to reflect the results of the experiments in an accurate and unbiased manner.
- ideally, figure panels should include only measurements that are directly comparable to each other and obtained with the same assay.
- plots include clearly labeled error bars for independent experiments and sample sizes. Unless justified, error bars should not be shown for technical
- if n<5, the individual data points from each experiment should be plotted. Any statistical test employed should be justified.
- Source Data should be included to report the data underlying figures according to the guidelines set out in the authorship guidelines on Data

2. Captions
Each figure caption should contain the following information, for each panel where they are relevant:
- a specification of the experimental system investigated (eg cell line, species name).
- the assay(s) and method(s) used to carry out the reported observations and measurements.
- an explicit mention of the biological and chemical entity(ies) that are being measured.
- an explicit mention of the biological and chemical entity(ies) that are altered/varied/perturbed in a controlled manner.
- the exact sample size (n) for each experimental group/condition, given as a number, not a range;
- a description of the sample collection allowing the reader to understand whether the samples represent technical or biological replicates (including how many animals, litters, cultures, etc.).
- a statement of how many times the experiment shown was independently replicated in the laboratory.
- definitions of statistical methods and measures:
  - common tests, such as t-test (please specify whether paired vs. unpaired), simple χ2 tests, Wilcoxon and Mann-Whitney tests, can be unambiguously identified by name only, but more complex techniques should be described in the methods section;
  - are tests one-sided or two-sided?
  - are there adjustments for multiple comparisons?
  - exact statistical test results, e.g., P values = x but not P values < x;
  - definition of 'center values' as median or average;
  - definition of error bars as s.d. or s.e.m.

Please complete ALL of the questions below.
Select "Not Applicable" only when the requested information is not relevant for your study.

Materials

Newly Created Materials	Information included in the manuscript?	In which section is the information available? (Reagents and Tools Table, Materials and Methods, Figures, Data Availability Section)
New materials and reagents need to be available; do any restrictions apply?	Yes	Materials & Methods

Antibodies	Information included in the manuscript?	In which section is the information available? (Reagents and Tools Table, Materials and Methods, Figures, Data Availability Section)
For **antibodies** provide the following information:<br>- Commercial antibodies: RRID (if possible) or supplier name, catalogue number and or/clone number<br>- Non-commercial: RRID or citation	Yes	Materials & Methods

DNA and RNA sequences	Information included in the manuscript?	In which section is the information available? (Reagents and Tools Table, Materials and Methods, Figures, Data Availability Section)
**Short novel DNA or RNA including primers, probes**: provide the sequences.	Yes	Appendix Tables S1 and S2

Cell materials	Information included in the manuscript?	In which section is the information available? (Reagents and Tools Table, Materials and Methods, Figures, Data Availability Section)
**Cell lines:** Provide species information, strain. Provide accession number in repository **OR** supplier name, catalog number, clone number, and/**OR** RRID.	Yes	Materials & Methods
**Primary cultures:** Provide species, strain, sex of origin, genetic modification status.	Yes	Materials & Methods
Report if the cell lines were recently **authenticated** (e.g., by STR profiling) and tested for mycoplasma contamination.	Yes	

Experimental animals	Information included in the manuscript?	In which section is the information available? (Reagents and Tools Table, Materials and Methods, Figures, Data Availability Section)
**Laboratory animals or Model organisms:** Provide species, strain, sex, age, genetic modification status. Provide accession number in repository **OR** supplier name, catalog number, clone number, **OR** RRID.	Yes	Materials & Methods
**Animal observed in or captured from the field:** Provide species, sex, and age where possible**.**	Not Applicable	
Please detail **housing and husbandry conditions**.	Yes	Materials & Methods

Plants and microbes	Information included in the manuscript?	In which section is the information available? (Reagents and Tools Table, Materials and Methods, Figures, Data Availability Section)
**Plants:** provide species and strain, ecotype and cultivar where relevant, unique accession number if available, and source (including location for collected wild specimens).	Not Applicable	
**Microbes:** provide species and strain, unique accession number if available, and source.	Not Applicable	

Human research participants	Information included in the manuscript?	In which section is the information available? (Reagents and Tools Table, Materials and Methods, Figures, Data Availability Section)
If collected and within the bounds of privacy constraints report on age, sex and gender or ethnicity for all study participants.	Yes	Materials & Methods

Core facilities	Information included in the manuscript?	In which section is the information available? (Reagents and Tools Table, Materials and Methods, Figures, Data Availability Section)
If your work benefited from core facilities, was their service mentioned in the acknowledgments section?	Yes	acknowledgement section

Design

Study protocol	Information included in the manuscript?	In which section is the information available? (Reagents and Tools Table, Materials and Methods, Figures, Data Availability Section)
If study protocol has been **pre-registered, provide DOI in the manuscript**. For clinical trials, provide the trial registration number **OR** cite DOI.	Not Applicable	
Report the **clinical trial registration number** (at ClinicalTrials.gov or equivalent), where applicable.	Not Applicable	

Laboratory protocol	Information included in the manuscript?	In which section is the information available? (Reagents and Tools Table, Materials and Methods, Figures, Data Availability Section)
Provide DOI OR other citation details if **external detailed step-by-step protocols** are available.	Not Applicable	

Experimental study design and statistics	Information included in the manuscript?	In which section is the information available? (Reagents and Tools Table, Materials and Methods, Figures, Data Availability Section)
Include a statement about **sample size** estimate even if no statistical methods were used.	Yes	Materials & Methods
Were any steps taken to minimize the effects of subjective bias when allocating animals/samples to treatment (e.g. **randomization procedure**)? If yes, have they been described?	Not Applicable	Materials & Methods
Include a statement about **blinding** even if no blinding was done.	Yes	Materials & Methods
Describe **inclusion/exclusion criteria** if samples or animals were excluded from the analysis. Were the criteria pre-established?<br><br>If sample or data points were omitted from analysis, report if this was due to attrition or intentional exclusion and provide justification.	Not Applicable	
For every figure, are **statistical tests** justified as appropriate? Do the data meet the assumptions of the tests (e.g., normal distribution)? Describe any methods used to assess it. Is there an estimate of variation within each group of data? Is the variance similar between the groups that are being statistically compared?	Yes	Materials & Methods

Sample definition and in-laboratory replication	Information included in the manuscript?	In which section is the information available? (Reagents and Tools Table, Materials and Methods, Figures, Data Availability Section)
In the figure legends: state number of times the experiment was **replicated** in laboratory.	Yes	Materials & Methods
In the figure legends: define whether data describe **technical or biological replicates**.	Yes	Materials & Methods

Ethics

Ethics	Information included in the manuscript?	In which section is the information available? (Reagents and Tools Table, Materials and Methods, Figures, Data Availability Section)
Studies involving **human participants**: State details of **authority granting ethics approval** (IRB or equivalent committee(s), provide reference number for approval.	Yes	Materials & Methods
Studies involving **human participants**: Include a statement confirming that **informed consent** was obtained from all subjects and that the experiments conformed to the principles set out in the WMA Declaration of Helsinki and the Department of Health and Human Services Belmont Report.	Yes	Materials & Methods
Studies involving **human participants:** For publication of **patient photos**, include a statement confirming that consent to publish was obtained.	Not Applicable	
Studies involving experimental **animals**: State details of **authority granting ethics approval** (IRB or equivalent committee(s), provide reference number for approval. Include a statement of compliance with ethical regulations.	Yes	Materials & Methods
Studies involving **specimen and field samples:** State if relevant **permits** obtained, provide details of authority approving study; if none were required, explain why.	Not Applicable	

Dual Use Research of Concern (DURC)	Information included in the manuscript?	In which section is the information available? (Reagents and Tools Table, Materials and Methods, Figures, Data Availability Section)
Could your study fall under dual use research restrictions? Please check biosecurity documents and list of **select agents and toxins** (CDC): https://www.selectagents.gov/sat/list.htm	Not Applicable	
If you used a select agent, is the security level of the lab appropriate and reported in the manuscript?	Not Applicable	
If a study is subject to dual use research of concern regulations, is the name of the **authority granting approval and reference number** for the regulatory approval provided in the manuscript?	Not Applicable	

Reporting

The MDAR framework recommends adoption of discipline-specific guidelines, established and endorsed through community initiatives. Journals have their own policy about requiring specific guidelines and recommendations to complement MDAR.

Adherence to community standards	Information included in the manuscript?	In which section is the information available? (Reagents and Tools Table, Materials and Methods, Figures, Data Availability Section)
State if relevant guidelines or checklists (e.g., **ICMJE, MIBBI, ARRIVE, PRISMA**) have been followed or provided.	Not Applicable	
For **tumor marker prognostic studies,** we recommend that you follow the **REMARK** reporting guidelines (see link list at top right). See author guidelines, under 'Reporting Guidelines'. Please confirm you have followed these guidelines.	Not Applicable	
For **phase II and III randomized controlled trials**, please refer to the **CONSORT** flow diagram (see link list at top right) and submit the CONSORT checklist (see link list at top right) with your submission. See author guidelines, under 'Reporting Guidelines'. Please confirm you have submitted this list.	Not Applicable	

Data Availability

Data availability	Information included in the manuscript?	In which section is the information available? (Reagents and Tools Table, Materials and Methods, Figures, Data Availability Section)
Have **primary datasets** been deposited according to the journal's guidelines (see 'Data Deposition' section) and the respective accession numbers provided in the Data Availability Section?	Yes	Data availability
Were **human clinical and genomic datasets** deposited in a public access-controlled repository in accordance to ethical obligations to the patients and to the applicable consent agreement?	Yes	Data availability
Are **computational models** that are central and integral to a study available without restrictions in a machine-readable form? Were the relevant accession numbers or links provided?	Not Applicable	
If publicly available data were reused, provide the respective **data citations in the reference list**.	Not Applicable	
